# Excitonic and Optical Transduction Mechanisms in Quantum Dot Sensors for Environmental Pollutant Detection

**DOI:** 10.3390/s26175675

**Published:** 2026-09-07

**Authors:** Christian Ebere Enyoh

**Affiliations:** Graduate School of Science and Engineering, Saitama University, Saitama 338-8570, Japan; cenyoh@gmail.com or enyoh@mail.saitama-u.ac.jp

**Keywords:** quantum dots, exciton, fluorescence sensing, environmental pollutants, photoluminescence, defect engineering, carbon quantum dots, PFAS, microplastics, heavy metals, FRET, machine learning

## Abstract

The accelerating contamination of global ecosystems by heavy metal ions, per- and polyfluoroalkyl substances (PFASs), microplastics and nanoplastics (MNPs), and emerging contaminants demands sensing technologies that are rapid, sensitive, selective, and field-deployable. Quantum dots (QDs) have emerged as leading candidates for environmental sensing; however, their performance is often interpreted empirically rather than through a unified understanding of the underlying excitonic physics. This narrative review presents a mechanistically integrated framework for QD-based environmental sensing, establishing the exciton, the spatially confined electron–hole quasiparticle, as the primary signal carrier in the most analytically powerful QD sensing modalities. A critical distinction is drawn between three categories of signal-generating processes: genuine excitonic transduction (photoinduced electron transfer, trap-state modulation, FRET, charge-transfer exciton formation, and binding energy modulation); non-excitonic optical phenomena, including the inner filter effect and light scattering, which are frequently misattributed as excitonic responses; and partially excitonic processes such as certain electrochemiluminescence pathways. Exciton fundamentals, confinement effects, and the influence of defects, dopants, and surface states are examined across carbon, chalcogenide, perovskite, and III–V QD families. A Defect–Exciton Energy Map is introduced as a rational design tool linking defect characteristics to excitonic response regime and sensing modality. Application of the mechanistic framework to heavy metal ions, PFASs, microplastics, and emerging contaminants demonstrates that sensing performance differences are mechanistically predictable from excitonic parameters rather than being arbitrary outcomes of materials choice. Benchmarking against competing platforms identifies conditions under which QD sensors offer genuine advantages. The roles of density functional theory, molecular dynamics, and machine learning in enabling rational sensor design are assessed. Key challenges, including stability, real-sample validation, standardisation, and toxicity, and future directions, including QD/two-dimensional material heterostructures and circular economy carbon QD platforms, are identified.

## 1. Introduction

The accelerating contamination of global ecosystems by synthetic chemical pollutants represents one of the defining environmental challenges of the twenty-first century. Heavy metal ions, per- and polyfluoroalkyl substances (PFASs), microplastics and nanoplastics, pesticides, nitroaromatics, and pharmaceutical residues have been detected across virtually every environmental compartment from deep ocean sediments to Arctic ice cores, and increasingly within human tissues and organs [1,2,3,4]. Microplastics alone number an estimated 5.25 trillion particles floating in the world’s oceans, releasing substantial dissolved organic carbon annually and disrupting microbial dynamics [5]. PFAS compounds, colloquially designated “forever chemicals” owing to their extraordinary environmental persistence, are now ubiquitous in freshwater systems, soils, and biological matrices, with their co-occurrence alongside microplastics creating synergistic exposure pathways that substantially amplify ecological and human health risks [6]. In a landmark clinical study, microplastics embedded in carotid artery plaques were associated with a fourfold increased risk of myocardial infarction, stroke, or death over a three-year follow-up period [7], a finding that underscores the urgent translational imperative of developing rapid, sensitive, and field-deployable detection platforms capable of tracking these contaminants in complex real-world matrices.

Conventional analytical methods for environmental pollutant detection, including inductively coupled plasma mass spectrometry (ICP-MS), high-performance liquid chromatography (HPLC), gas chromatography-mass spectrometry (GC-MS), and atomic absorption spectroscopy (AAS), deliver high sensitivity and chemical specificity but remain fundamentally constrained by requirements for centralised laboratory infrastructure, extensive sample pre-treatment protocols, trained technical personnel, and high per-analysis costs [8,9]. These limitations create a critical surveillance gap: the very environments most in need of continuous monitoring remote watersheds, agricultural soils, coastal fisheries, and low-resource settings are precisely those least accessible to laboratory-centric workflows. There is therefore an urgent need for sensing platforms that combine high analytical performance with operational simplicity, miniaturisation potential, and the capacity for real-time or near-real-time signal transduction [10].

Quantum dots (QDs) have emerged over the past two decades as among the most promising nanoscale platforms for addressing this challenge. These semiconductor nanoparticles, typically smaller than 10 nm, exhibit strong, relatively stable, and tunable luminescent properties that arise from quantum confinement of charge carriers within the particle volume [11]. When an electron is photoexcited across the bandgap, it leaves behind a positively charged vacancy in the valence band; these two opposite charges are bound by Coulombic attraction in a quasiparticle known as an exciton, whose spatial extent is defined by the exciton Bohr radius [12]. In a nanostructure whose physical dimensions approach this Bohr radius, the exciton is spatially confined, which discretises the energy levels and widens the effective bandgap relative to the bulk semiconductor. This quantum confinement phenomenon is the physical origin of the size-dependent optical tunability that distinguishes QDs from conventional molecular fluorophores: by controlling particle size during synthesis, emission wavelengths can be systematically shifted from the ultraviolet through the visible and into the near-infrared spectral regions within a single material composition [13]. Unlike bulk materials with a continuous bandgap, the QD bandgap is inversely related to particle size, so that smaller QDs exhibit stronger quantum confinement and higher-energy optical transitions [14].

Beyond size-tunability, QDs offer a constellation of photophysical properties that collectively render them superior to organic fluorophores for analytical sensing. It has been estimated that QDs are approximately 20 times brighter and 100 times more stable against photobleaching than traditional fluorescent reporters [15], a property that translates directly into lower limits of detection and extended operational lifetimes in sensing devices. Their broad absorption spectra permit excitation by a single light source even when multiple QD populations are used simultaneously, enabling multiplexed detection of several analytes within a single measurement. High surface-to-volume ratios facilitate the attachment of diverse recognition elements antibodies, aptamers, molecularly imprinted polymers, and functional ligands through well-established surface chemistry, conferring analyte selectivity upon an inherently non-selective optical core [16]. These properties have driven rapid growth in QD-based sensing research: the recent literature reflects a multidimensional and interdisciplinary perspective, combining materials science, environmental chemistry, nanotechnology, and sustainability analysis to develop QD sensors targeting analytes ranging from heavy metal ions and pesticides to biological toxins and gaseous pollutants [17].

The QD family is itself remarkably diverse. Traditional semiconductor QDs most notably cadmium chalcogenides (CdSe, CdS, CdTe) and zinc chalcogenides (ZnS, ZnSe) established the field’s photophysical foundations and remain benchmark materials for high quantum yield and narrow emission linewidths [18]. Core–shell architectures such as CdSe/ZnS introduced the concept of shell-mediated exciton passivation, dramatically improving photoluminescence quantum yield by suppressing non-radiative recombination at surface defect sites [19]. More recently, perovskite quantum dots (PQDs), particularly the all-inorganic CsPbX_3_ series (X = Cl, Br, I), have attracted intense interest owing to their exceptionally high photoluminescence quantum yields, narrow emission linewidths, and compositionally tunable bandgaps spanning the entire visible spectrum [20]. The high exciton binding energy in PQDs, reaching up to 100 meV in some compositions, enhances radiative recombination efficiency and makes them highly sensitive to subtle changes in surface chemistry induced by analyte interactions [21]. Carbon quantum dots (CQDs), synthesised from diverse carbon precursors, including renewable biomass and polymer waste streams, have emerged as a non-toxic, earth-abundant alternative to heavy metal-based QDs [22]. CQDs offer tunable fluorescence, high biocompatibility, and sustainable synthesis routes, with the underlying photophysical mechanisms, including quantum confinement, surface functionalisation, and heteroatom doping, governing their fluorescence modulation properties [23]. The quantum confinement effect in CQDs restricts excitons within nanoscale carbon cores typically below 10 nm, producing discrete energy levels and size-tunable fluorescence emission whose radiative efficiency and spectral position respond sensitively to changes in the local chemical environment [24].

Despite the substantial and growing literature on QD-based environmental sensing, a fundamental conceptual gap persists. Existing reviews overwhelmingly treat sensing performance expressed as limits of detection (LOD) and linear dynamic ranges as an empirical outcome of materials synthesis choices and surface functionalisation strategies, without providing a mechanistic account of why specific QD families or defect configurations produce superior responses toward particular analyte classes. Analytical assessments drawing on over one hundred peer-reviewed studies have focused primarily on figures of merit such as LOD, excitation and emission wavelengths, and particle size, without establishing a unifying mechanistic framework linking excitonic parameters to sensing performance [8,25,26]. The result is a literature rich in empirical demonstrations but deficient in the mechanistic insight needed to guide rational materials design.

The exciton is the natural organising principle for such a framework. However, it is important to distinguish at the outset between three categories of signal-generating processes in QD-based sensors. The first genuine excitonic transduction encompasses processes in which analyte interaction directly modifies the excitonic state: its binding energy, radiative lifetime, oscillator strength, or spatial localisation [12], including photoinduced electron transfer, trap-state creation and passivation, FRET, charge-transfer exciton formation, and dielectric environment modulation. The second, non-excitonic optical phenomena include the inner filter effect (IFE) and light scattering by particulate analytes, which produce apparent fluorescence changes without perturbing the excitonic state and represent a major source of mechanistic misattribution in the sensing literature [27]. The third, partially excitonic processes include certain electrochemiluminescence pathways where the excitonic character of the emitting state depends on the specific coreactant mechanism. The mechanistic framework developed in this narrative review applies rigorously to the first category, explicitly identifies the second as requiring experimental correction rather than mechanistic interpretation, and addresses the third with appropriate qualification throughout.

Against this background, this narrative review makes four contributions that, to our knowledge, have not been made simultaneously or in the form presented here by any existing QD sensing review. First, it introduces a tripartite mechanistic classification of genuine excitonic transduction, non-excitonic optical artefacts, and partially excitonic processes, applied consistently across all mechanisms, QD families, and pollutant classes, correcting a systematic conflation that pervades the primary literature. Second, it introduces the Defect–Exciton Energy Map: a two-dimensional predictive design space that maps defect density and depth to excitonic response regime and sensing modality, enabling a priori specification of the QD architecture, surface chemistry, and dominant transduction mechanism required for a target sensing challenge demonstrated through a worked predictive example. Third, it provides a critical evaluation of analytical techniques for mechanism discrimination, establishing minimum evidence standards for mechanistic claims in QD sensing publications. Fourth, it integrates first-principles DFT/TD-DFT defect characterisation, molecular dynamics simulation of QD-analyte binding, and machine learning property prediction within a unified excitonic design framework connected explicitly to inverse design objectives.

This narrative review draws on the literature spanning carbon, chalcogenide, perovskite, and III–V QD families and four major pollutant classes, with particular emphasis on studies published from 2018 to 2026. The literature was selected to represent mechanistically significant findings and recent advances rather than to catalogue materials or compile detection limits. The review is structured as follows: Section 2 establishes exciton fundamentals; Section 3 surveys excitonic properties across QD families; Section 4 examines defect, dopant, and surface-state modulation, including the Defect–Exciton Energy Map; Section 5 develops the mechanistic transduction taxonomy; Section 6 critically evaluates analytical probing techniques; Section 7 applies the framework to four pollutant classes; Section 8 benchmarks QD sensors against competing platforms; Section 9 examines computational design approaches; and Section 10 and Section 11 address open challenges and future directions, respectively.

## 2. Fundamentals of Excitons in Quantum Dots

**Figure 1:**
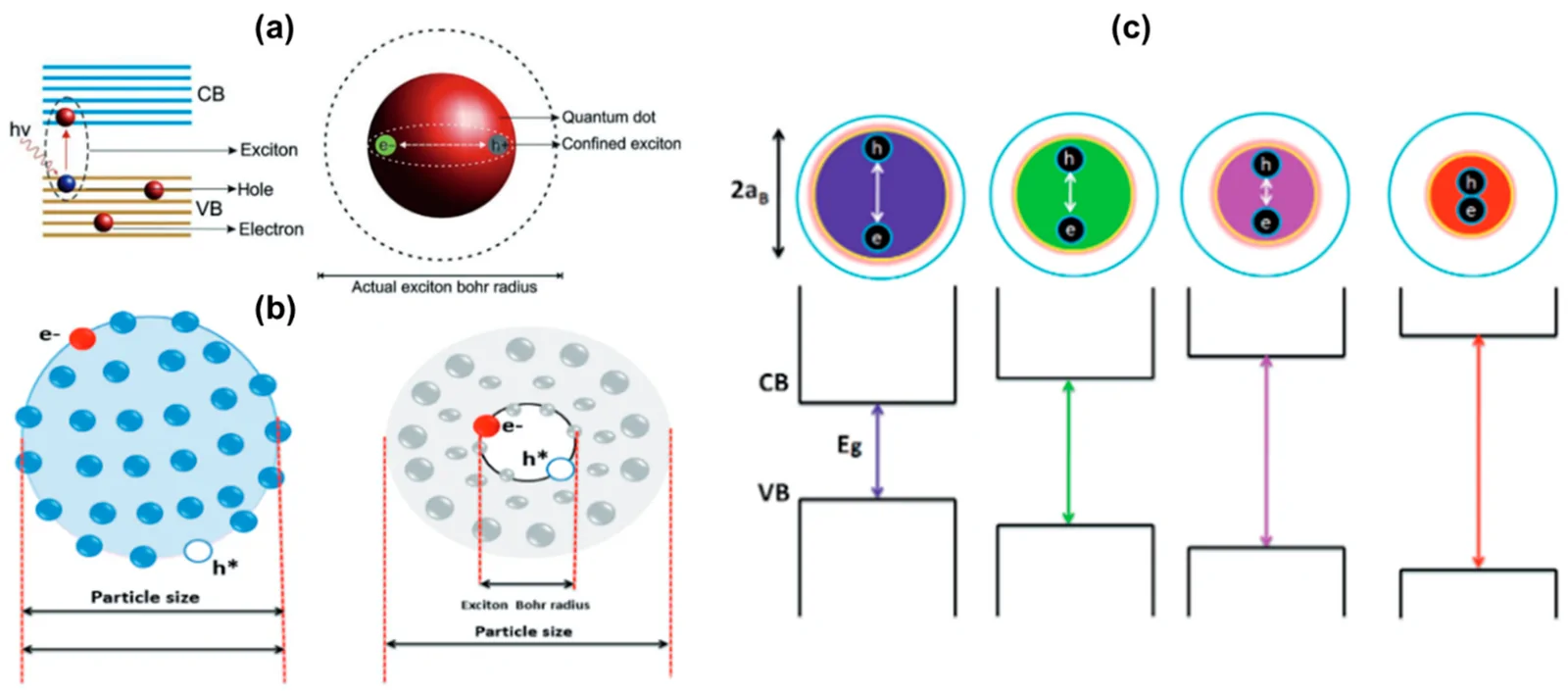
Fundamental excitonic processes in quantum dots (QDs). (**a**) Formation of an exciton through photoexcitation, showing electron promotion from the valence band (VB) to the conduction band (CB) and the subsequent Coulomb-bound electron–hole pair. (**b**) Comparison between exciton Bohr radius and QD particle size, illustrating the transition from bulk-like behaviour to quantum-confined excitonic states when QD dimensions approach or fall below the exciton radius. (**c**) Quantum confinement effect in semiconductor QDs, showing size-dependent modulation of the bandgap energy and corresponding changes in electron–hole energy levels and optical transitions (adapted from [29]).

The exciton, a bound quasiparticle consisting of a photoexcited electron and the positively charged vacancy (hole) generated in the valence band (Figure 1a), represents the fundamental excited-state entity governing the optical behaviour of quantum dots (QDs). The concept was first introduced by Frenkel [28] to describe electronic excitations in ionic insulators, where electrons and holes remain bound through Coulombic interactions while the excitation can migrate through the lattice without net charge transport. In semiconductor QDs, exciton formation is the primary photophysical event underlying light absorption, emission, and ultimately optical sensing responses. Upon absorption of a photon with energy exceeding the bandgap, an electron is promoted from the valence band (VB) to the conduction band (CB), leaving behind a hole. The electron and hole are not independent carriers but remain coupled through electrostatic attraction, forming a hydrogen-like bound state characterised by the exciton Bohr radius (aB) (Figure 1b,c). Similar to the hydrogen atom, the exciton Bohr radius represents the spatial extent of the electron–hole pair but is determined by the semiconductor dielectric constant and the effective masses of the charge carriers. This parameter defines the critical length scale at which quantum confinement begins to modify the electronic structure and optical properties of QDs [29].

The exciton Bohr radius varies substantially among QD materials, ranging from approximately ~2.5 nm in CdS to ~5.6 nm in CdSe and ~46 nm in PbSe, resulting in significant differences in confinement behaviour and size-dependent optical responses [30]. When QD dimensions become comparable to or smaller than the exciton Bohr radius, quantum confinement alters the continuous electronic states of bulk semiconductors into discrete energy levels. Three confinement regimes, including weak, intermediate, and strong, are therefore distinguished based on the relationship between QD size and exciton dimensions [14,31]. In the strong confinement regime, where the QD radius is smaller than both the electron and hole Bohr radii, electron and hole motion becomes spatially restricted, producing pronounced energy quantisation and an enlarged bandgap relative to the bulk material (Figure 1c) [29]. This regime dominates many sensing-relevant QDs, where excitonic properties become highly sensitive to particle size, composition, surface defects, and environmental interactions, making exciton engineering central to the design of QD-based optical sensors.

### 2.1. Quantum Confinement and the Brus Equation

The quantitative relationship between particle size, material composition, and bandgap energy in the strong confinement regime is most commonly described by the Brus equation, which expresses the total excitonic transition energy as a sum of the bulk bandgap energy, a positive confinement energy term scaling as *R*^−2^, and a negative Coulombic correction term representing the electron-hole attraction energy [13]:

(1) $$E_{QD}=E_{g,bulk}+\frac{{\bar{h}}^{2}\pi^{2}}{{2\mu R}^{2}}-\;\frac{{1.8e}^{2}}{{4\pi \varepsilon }_{0}\varepsilon_{r}R}$$

where *R* is the QD radius, *μ* is the reduced mass of the electron-hole pair, $\bar{h}$ is the reduced Planck constant (h/2), *ε_r_* is the dielectric constant of the semiconductor, and *E_{g,bulk}_* is the bulk bandgap. The confinement energy, i.e., the dominant term for small QDs, is inversely proportional to the square of the particle radius, meaning that small changes in particle size produce large shifts in absorption and emission wavelength [32]. This relationship underpins the synthetic tunability that makes QDs uniquely valuable as sensing platforms: a single material composition, CdSe, for example, can be tuned continuously from green emission (~2.3 nm diameter) to deep red (~7 nm diameter) simply by controlling particle size during synthesis [33]. Unlike bulk materials with a constant bandgap, the QD bandgap is inversely related to particle size, such that smaller QDs exhibit stronger quantum confinement and higher-energy optical transitions [14]. For sensing applications, this tunability enables the rational matching of QD emission to the absorption band of a target analyte, a prerequisite for efficient FRET-based detection and the design of ratiometric sensors operating across widely separated spectral windows.

### 2.2. Exciton Binding Energy and Its Sensing Significance

The exciton binding energy (*E*_b_) is the energy required to dissociate a bound electron-hole pair into free carriers [24]. It is an important parameter in the photophysics of QDs, but it is one of several coupled quantities alongside oscillator strength, wavefunction overlap integral, non-radiative recombination rates, and surface trap density that together determine photoluminescence quantum yield (PLQY) and radiative rate. These quantities are correlated but not equivalent, and conflating *E*_b_ with PLQY or radiative rate introduces physical inconsistency that must be avoided in a mechanistically rigorous treatment. In bulk semiconductors, *E*_b_ values are typically modest approximately 4 meV in silicon and 4.2 meV in gallium arsenide and thermal energy at room temperature (*k*_BT_ ≈ 26 meV at 300 K) is sufficient to ionise most excitons, rendering them transient [34]. In QDs, spatial confinement reduces the average electron-hole separation, dramatically enhancing the Coulombic overlap between the electron and hole wavefunctions and increasing *E*_b_ substantially relative to the bulk value. In perovskite QDs, for example, *E*_b_ values reaching up to 100 meV in some compositions have been reported, ensuring that excitons remain bound at room temperature and are the dominant excited-state species for optical transduction [21]. This thermal stability of the exciton is a necessary, though not sufficient, condition for efficient radiative emission: a bound exciton that remains intact at room temperature can recombine radiatively, but whether it does so efficiently depends additionally on the magnitude of the oscillator strength and the density of competing non-radiative pathways, particularly surface trap states.

It is important to clarify the relationship between *E_b_*, radiative rate, and PLQY, which is more nuanced than a simple monotonic dependence. The radiative recombination rate (*k*_r_) is determined primarily by the oscillator strength of the excitonic transition, which scales with the square of the electron-hole wavefunction overlap integral, a quantity that is enhanced by quantum confinement but is not directly equivalent to *E*_b_. The PLQY is then given by:

(2) $$PLQY=\;\frac{k_{r}}{k_{r}+k_{nr}}$$

where *k*_nr_ is the total non-radiative recombination rate, dominated in most QD systems by surface trap-mediated processes. A higher *E*_b_ is associated with stronger spatial confinement and greater wavefunction overlap, which tends to increase *k*_r_. However, PLQY is ultimately governed by the competition between *k*_r_ and *k*_nr_, meaning that a QD with high *E*_b_ but a large surface trap density will still exhibit low PLQY despite its high binding energy. Conversely, a well-passivated QD with moderate *E*_b_ can achieve near-unity PLQY if non-radiative channels are effectively suppressed [35]. The statement that “a higher *E*_b_ generally favours higher PLQY” is therefore a tendency rather than a rule, and it holds only when non-radiative pathways are held approximately constant across the comparison, a condition that must be stated explicitly.

For sensing, *E*_b_ has dual analytical significance. First, it determines how readily an analyte interaction can perturb the excitonic state: analytes that introduce competing Coulombic interactions, alter the local dielectric environment, or coordinate to surface sites can modulate *E*_b_ and thereby shift emission energy or alter the balance between radiative and non-radiative recombination. Second, a higher *E*_b_ ensures that the exciton remains bound under the thermal and chemical conditions of the sensing measurement, a prerequisite for any intensity-based or spectral sensing modality that relies on excitonic emission as the analytical signal [36]. The sensing relevance of *E*_b_ is therefore best understood not as a direct predictor of PLQY or brightness, but as a parameter that governs excitonic thermal stability and susceptibility to analyte-induced perturbation, two properties that jointly determine the operating window and sensitivity of the sensor [24].

### 2.3. Bright and Dark Excitons: Fine Structure and Sensing Consequences

A bright-dark exciton refers to whether an electron and a hole bound together inside a quantum dot can emit light (bright) or are temporarily trapped, unable to release their energy as a photon (dark). In QDs, the lowest excitonic state is not a single level but rather a multiplet of states split by electron-hole exchange interactions, crystal field asymmetries, and shape anisotropy [37,38]. This fine structure partitions the exciton population into optically active “bright” states, which couple to electromagnetic radiation and contribute to PL, and optically inactive “dark” states, which do not directly emit. Dark exciton states exhibit substantially reduced decay rates, resulting in lifetimes that can be orders of magnitude longer than their optically bright counterparts [39]. This makes them attractive candidates for quantum information processing [40]. The energy separation between bright and dark states is typically on the millielectronvolt scale in chalcogenide QDs, meaning that at room temperature both populations are thermally accessible, and the measured PL reflects a thermally weighted average over the fine structure manifold [11].

For sensing applications, the bright-dark exciton balance has several important implications. The temperature dependence of emission intensity in QD sensors is partly governed by thermal redistribution between bright and dark states, meaning that a sensing measurement conducted at different temperatures can yield different apparent analyte responses unless the temperature is controlled [41,42,43,44]. Supporting this, Brodu [42] investigated the exciton fine structure of InP/ZnSe core/shell QDs and demonstrated that the lowest excitonic state is a dark exciton. Temperature-dependent PL measurements revealed that radiative recombination from this state is phonon-assisted, involving confined acoustic phonons with energies of 4–7 meV, while fluorescence line-narrowing spectroscopy showed additional coupling to optical phonons. These findings highlight the important role of exciton fine structure and exciton–phonon interactions in governing emission dynamics and temperature-dependent optical responses. This mechanism, while underappreciated in the sensing literature, represents a form of excitonic transduction that is distinct from classical quenching and merits explicit consideration when interpreting intensity-based sensing data.

### 2.4. Exciton–Phonon Coupling

The interaction between confined excitons and lattice vibrations, i.e., exciton-phonon coupling, is a critical determinant of emission linewidth, spectral stability, and the temperature dependence of photoluminescence in QD-based sensors. Two principal coupling channels exist: coupling to acoustic phonons, which produces linear temperature-dependent linewidth broadening and accounts for dephasing at low temperatures [42]; and coupling to longitudinal optical (LO) phonons, which dominates at elevated temperatures and drives thermally activated non-radiative recombination. Quantum confinement enhances coupling to acoustic phonons substantially relative to bulk values, while the coupling constant with LO phonons tends to decrease as QD size decreases [45]. This contrasting size dependence of the two coupling channels means that the temperature response of a QD sensor is inherently size-dependent and must be characterised for each specific material and size regime.

As temperature increases, interaction between carriers and acoustic phonons intensifies, contributing to emission linewidth broadening described by the Boson model, while thermally activated redistribution of holes between quantised levels leads to PL intensity quenching that can be described by an Arrhenius model [45]. For environmental sensing applications where field measurements may be conducted across a range of ambient temperatures, these thermal effects represent a significant source of measurement uncertainty if not corrected for. They also suggest a design principle: QDs with strong exciton binding energy and weak exciton-phonon coupling will exhibit more thermally stable emission signals, making them preferable for quantitative field sensing over those with highly temperature-sensitive luminescence. In ternary QD systems such as InGaP, both composition and size can be used to tune not only the bandgap but also exciton-phonon coupling and relaxation dynamics, a degree of compositional control not available in binary systems, and one that opens pathways for engineering temperature-robust sensing materials [11].

### 2.5. Multiexciton States: Biexcitons, Trions, and Auger Recombination

Under high excitation intensity conditions relevant to laser-excited sensing platforms and high-power analytical instrumentation, multiple electron-hole pairs can be generated within a single QD on timescales shorter than their recombination lifetime, producing multiexciton states. The biexciton (two electron-hole pairs) and charged exciton or trion (one electron-hole pair plus an additional carrier) are the most experimentally significant of these states [46]. Their photophysical properties differ markedly from those of the single exciton and introduce important considerations for high-sensitivity sensing design.

The dominant recombination pathway for multiexcitons in QDs is Auger recombination, a non-radiative process in which the energy released by the recombination of one electron-hole pair is transferred to a third carrier (electron or hole), which is then excited to a higher energy level rather than emitting a photon [47]. Auger non-radiative recombination of multi-carrier states leads to efficiency roll-off in QD-based devices and underpins the phenomenon of PL blinking (the random switching of emission between a bright neutral exciton state and a dim charged state with efficient Auger quenching) [47]. For sensing, Auger recombination introduces two complications. First, blinking at the single-dot level introduces stochastic fluctuations in the analytical signal that must be averaged over many particles or long acquisition times to obtain reproducible intensity readings. Second, at high excitation densities used to maximise signal, multiexciton Auger quenching reduces effective quantum yield and can cause apparent analyte-independent intensity decreases that mimic quenching-based sensor responses [48]. These considerations motivate the use of core–shell architectures, particularly thick-shell “giant” QDs, which spatially separate the electron and hole wavefunctions from the surface and effectively suppress Auger rates by reducing wavefunction overlap [47]. They also motivate the adoption of lifetime-based sensing modalities, where the analytical signal is encoded in the exciton lifetime rather than emission intensity, rendering the measurement insensitive to fluctuations in excitation power or QD concentration that differentially affect multiexciton contributions.

### 2.6. The Exciton as the Unifying Sensing Parameter

**Figure 2:**
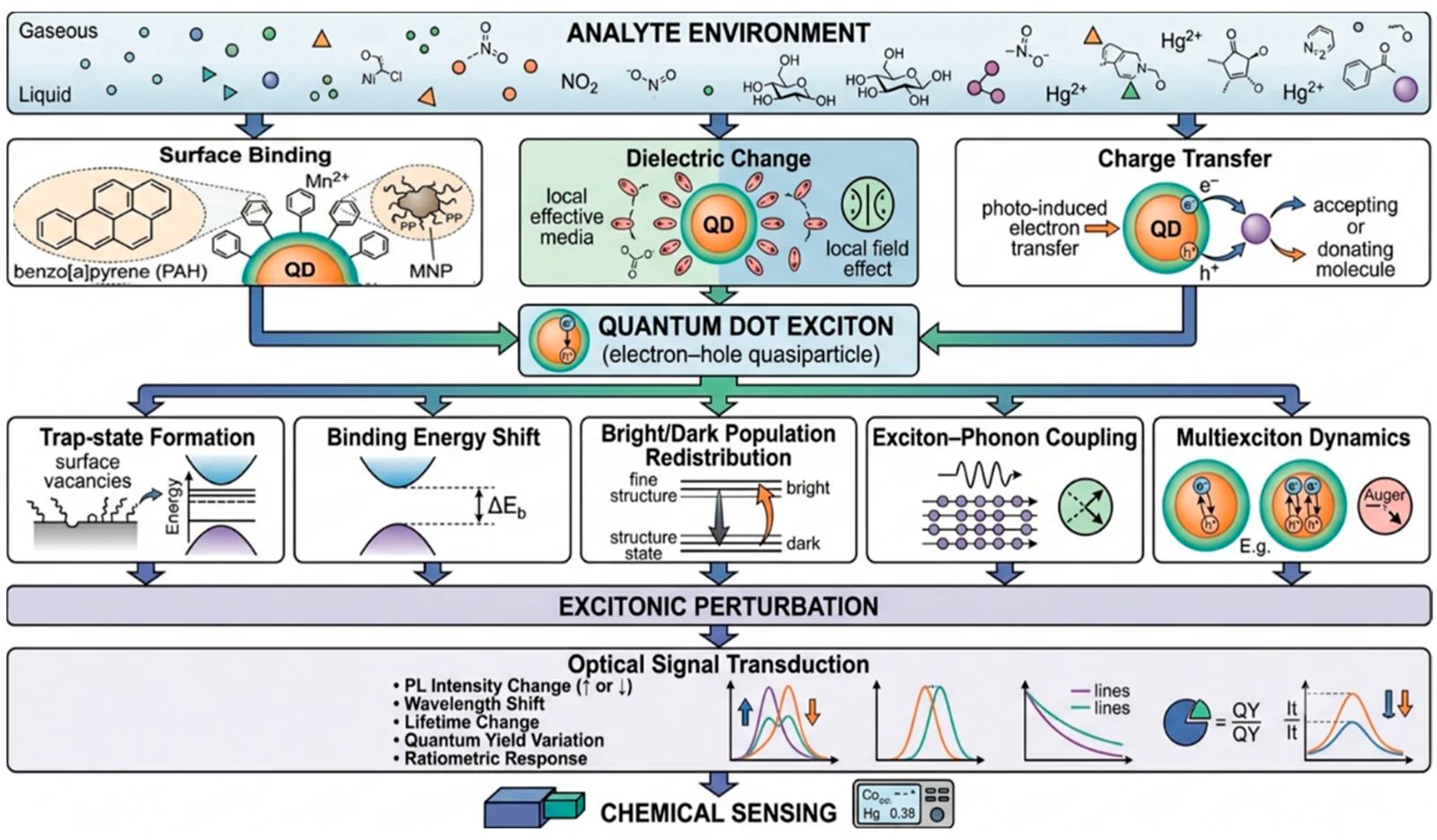
Exciton-centred framework for quantum-dot sensing. Analyte interactions modify the local physicochemical environment of a quantum dot through processes such as surface adsorption, dielectric screening, and charge transfer. These interactions perturb key excitonic parameters, including trap-state formation, exciton binding energy, fine-structure populations, exciton–phonon coupling, and multiexciton dynamics. The resulting excitonic perturbations manifest as measurable changes in PL intensity, spectral position, lifetime, and quantum yield, establishing the exciton as the central transduction element governing QD-based sensing.

The preceding subsections demonstrate that the exciton in a quantum dot (QD) is not a static emitter but a dynamically responsive quasiparticle whose energy, spatial extent, binding strength, fine structure, phonon coupling, and multiexciton behaviour are all sensitive to perturbations from the local chemical environment (Figure 2). Rather than representing independent phenomena, these properties constitute interconnected manifestations of excitonic behaviour that collectively determine the optical response of a QD. Consequently, the exciton serves as the central physical entity through which external chemical stimuli are translated into measurable signals.

This environmental sensitivity, which in conventional fluorophores often contributes to measurement uncertainty, becomes the analytical foundation of QD-based sensing. As illustrated in Figure 2, analyte interactions perturb the excitonic landscape through multiple pathways. Surface adsorption may create or passivate trap states, thereby altering the balance between radiative and non-radiative recombination. Variations in the dielectric environment modify exciton binding energy and emission energy, resulting in spectral shifts. Charge-transfer processes can dissociate excitons, introduce interfacial electronic states, or modify carrier populations, leading to fluorescence quenching or enhancement. Similarly, changes in bright–dark state populations, exciton–phonon coupling, or multiexciton dynamics can influence emission intensity, lifetime, quantum yield, and other measurable optical parameters.

Viewed from this perspective, the diverse sensing mechanisms reported across the QD literature are unified by a common principle: the analyte does not directly generate the optical signal but instead perturbs one or more excitonic parameters, which subsequently manifest as changes in PL properties. Understanding sensing responses at the excitonic level rather than treating them as empirical fluorescence changes enables the rational prediction, optimisation, and engineering of sensor performance [12,35].

This exciton-centred framework provides the conceptual foundation for the remainder of this review. The wide variability in sensing behaviour observed across different QD families, analyte classes, and detection modalities can be interpreted through the extent to which a given interaction modifies excitonic properties. Consequently, excitonic parameters represent a unifying set of descriptors that not only explain observed sensing responses but also provide experimentally and computationally accessible targets for the design of next-generation QD sensors.

## 3. QD Families: Structures, Excitonic Signatures, and Tuning Handles

**Figure 3:**
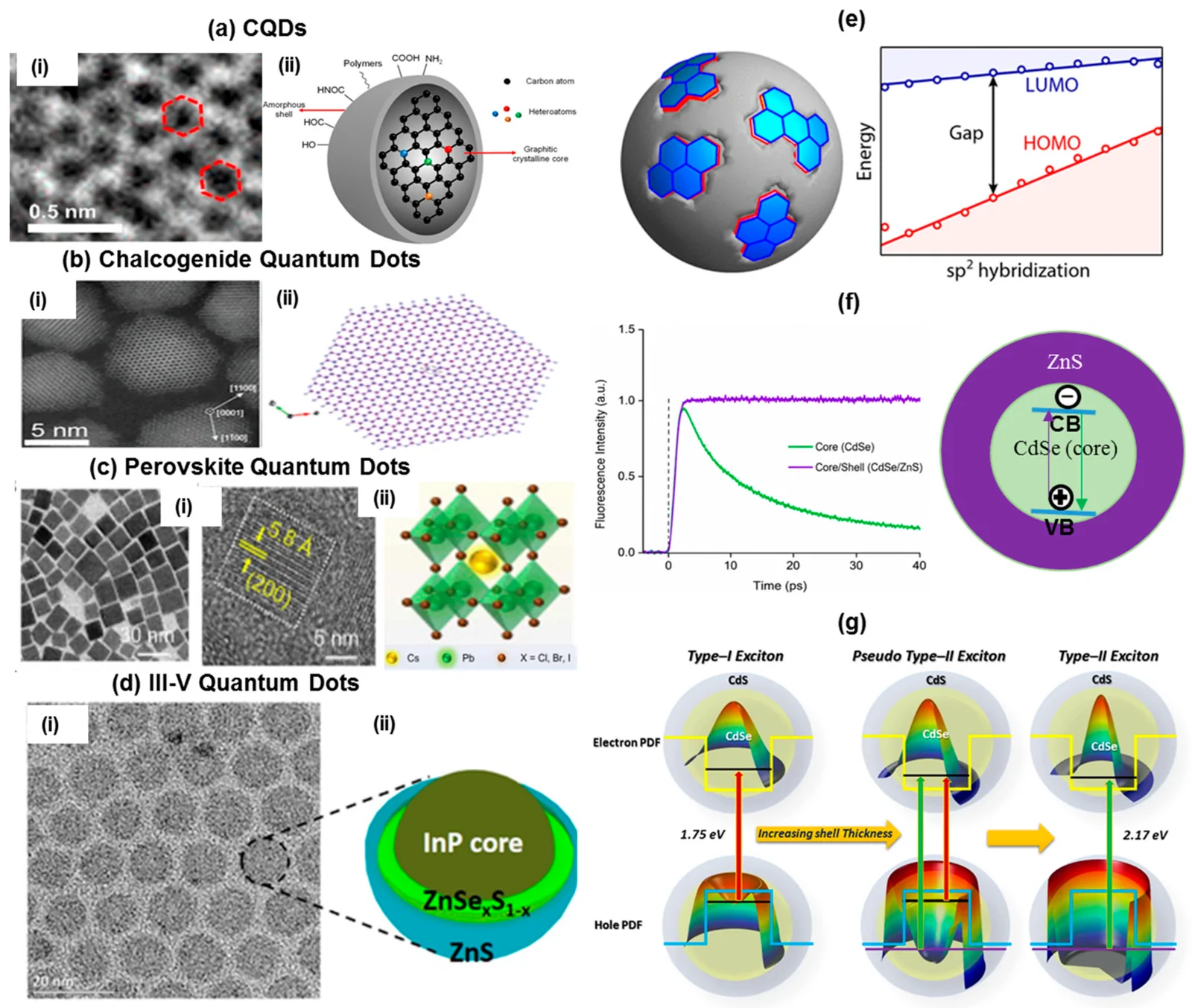
Representative quantum dot (QD) families, structures, and excitonic characteristics. Showing (**i**) TEM images and (**ii**) corresponding structural schematics of (**a**) carbon quantum dots (CQDs), comprising a graphitic carbon core with surface functional groups (adapted from [49]); (**b**) chalcogenide quantum dots, represented by CdSe/CdS QDs (adapted from [50]); (**c**) perovskite quantum dots, represented by CsPbX_3_ (X = Cl, Br, I) nanocrystals (adapted from [51]); and (**d**) III–V quantum dots, represented by InP-based InP/ZnSe_x_S_1−x_/ZnS core/shell nanostructures (adapted from [51]). The figure highlights the compositional and structural diversity of major QD classes that underpin their distinct optical, electronic, and excitonic properties relevant to sensing applications. (**e**) Influence of sp^2^-hybridised graphitic domains on the electronic structure and energy gap of CQDs (adapted from [52]). (**f**) Effect of core-only and core/shell architectures on exciton confinement and fluorescence behaviour, illustrating enhanced surface passivation and carrier confinement in CdSe/ZnS nanocrystals. (**g**) Schematic representation of type-I, quasi-type-II, and type-II excitons, showing the evolution of electron and hole spatial distributions with increasing shell thickness and the resulting changes in exciton localisation and emission energy (adapted from [53]).

The QD landscape available to the environmental sensing community encompasses four principal material families (Figure 3), each with a distinct photophysical identity, excitonic character, and set of synthetic tuning handles. Understanding these family-level differences is essential because the choice of QD material determines not only the accessible spectral window and PLQY but also, and more critically for sensing, the nature, energy, and environmental sensitivity of the excitonic states that transduce the analytical signal. This section surveys each family through the lens of its excitonic properties, drawing explicit connections to sensing utility.

### 3.1. Carbon Quantum Dots (CQDs)

Carbon quantum dots are zero-dimensional (0D) carbon-based nanomaterials, typically 1–10 nm in diameter, that have attracted extraordinary research interest as sustainable, low-toxicity alternatives to heavy metal-based semiconductor QDs [54]. Their photophysics, however, is considerably more complex than that of traditional chalcogenide QDs, because their emission arises from the coexistence and competition of multiple excited-state origins rather than a single band-edge exciton. The structural architecture of CQDs consists of an sp^3^-hybridised amorphous carbon core interspersed with, or decorated by, sp^2^-hybridised graphitic domains (Figure 3a(i,ii)). Tepliakov [52] showed that all the distinctive optical properties of CQDs, including their giant Stokes shift and strong dependence of emission colour on excitation wavelength can be explained by the linear optical response of the partially sp^2^-hybridised carbon domains located on the surface of the sp^3^-hybridised amorphous cores (Figure 3e). The domain hybridisation factor governs the localisation of excitons and the electronic bandgap within each domain, and the distribution of domain sizes and hybridisation states across a polydisperse CQDs population produces the characteristic excitation-dependent emission [55]. Carbon-core emission arising from π–π* transitions within the sp^2^ network tends to appear at shorter wavelengths; surface-state emission from functional groups, edge defects, and sp^3^ carbon irregularities typically dominates at longer wavelengths and is more responsive to the local chemical environment. The ground state shifts into multiple discrete states due to perturbation driven by surface functional groups, generating ground state heterogeneity that causes the emission spectrum to redshift as the excitation wavelength increases [56]. Both sp^3^- and sp^2^-hybridised carbons and other surface defects of CQDs can serve as capture centres for excitons and thus result in surface state-related fluorescence, with the surface state representing a synergistic hybridisation of carbon backbones and connected functional groups whose energy gap correlates with the extent of the π-electron system and surface chemistry [57]. This surface state-mediated excitonic capture is, from a sensing perspective, simultaneously the most valuable and most challenging aspect of CQDs photophysics. It is valuable because surface-state excitons are directly accessible to analyte interactions at the QD surface, meaning that binding events can perturb the excitonic landscape with high sensitivity. It is challenging because multiple coexisting emission centres complicate signal interpretation, lower apparent quantum yields through competition between radiative channels, and produce batch-to-batch variability if surface chemistry is not rigorously controlled.

Heteroatom doping principally with nitrogen, boron, sulphur, and phosphorus is one of the most powerful synthetic handle for engineering CQD excitonic properties [58]. Nitrogen doping, in particular, is well established as a route to enhanced quantum yields through n–π* transition activation and improved radiative recombination efficiency at surface trap sites, with higher quantum yields translating directly to stronger and more analytically useful fluorescence signals [24,59]. Surface defects arising from the unsymmetrical arrangement of sp^2^ and sp^3^ carbon atoms and the presence of heteroatoms like N, B, P, or S create environments that produce various colour emissions from UV absorption [10,24]. CQDs absorb UV–visible–NIR light primarily through π–π* transitions of sp^2^-hybridised carbon networks and n–π* transitions when heteroatoms such as nitrogen or sulphur are incorporated, with their steady, non-blinking photoluminescence and enhanced photostability surpassing those of organic fluorophores [55].

A particularly important excitonic phenomenon in CQDs for sensing applications is aggregation-induced emission (AIE) [60,61]. Inter-dots/molecular excitonic coupling and intra-dots/molecular electron-vibration couplings are the two key mechanisms governing both aggregation-caused quenching and AIE in CQDs [60], a mechanistic understanding with direct relevance to designing CQD sensors that operate in high-ionic-strength environmental matrices where aggregation is thermodynamically favourable [62].

### 3.2. Chalcogenide QDs (CdSe, CdS, CdTe, ZnS, and Core–Shell Architectures)

Chalcogenide QDs, principally cadmium and zinc chalcogenides, established the foundational framework of QD photophysics and remain the most extensively characterised QD family for sensing applications [25]. Their excitonic properties are better understood than those of any other QD family, which makes them uniquely valuable as reference materials against which newer QD families are benchmarked. Core-only CdSe QDs in the 2–8 nm diameter range exhibit band-edge excitons with binding energies of approximately 10–25 meV in the bulk [63], increased substantially due to confinement in nanocrystalline form [64]. Their PLQYs in the core-only configuration are modest due to surface trap states arising from unsatisfied surface bonds (dangling bonds) that compete efficiently with radiative recombination. The introduction of a wider-bandgap shell, most commonly ZnS or CdS, produces core–shell architectures whose excitonic character depends critically on the band alignment between core and shell materials (Figure 3f) [64]. In the type-I core–shell structure, the conduction band and valence band energies of the shell are higher and lower, respectively, than those of the core, confining both charge carriers within the core and resulting in strong, spectrally pure luminescence [53]. This configuration, exemplified by CdSe/ZnS, maximises the exciton wavefunction overlap within the core, producing high radiative recombination rates, near-unity quantum yields in optimised samples, and narrow emission linewidths. Upon increasing shell thickness in CdSe/CdS core–shell QDs, the radial distribution function of the hole shifts towards the shell, causing the confinement regime to switch from type-I to type-II excitons, with an intermediate pseudo type-II state appearing where holes are co-localised in the shell as well as the core while electrons are confined to the core only (Figure 3g) [53]. In a sensing context, the type-II spatial separation of the exciton is more susceptible to perturbation by analytes that selectively interact with the core or shell material, making type-II structures promising for charge-transfer-based sensing mechanisms [11]. CdTe QDs offer a narrower bulk bandgap than CdSe (1.44 eV vs. 1.74 eV) [65], extending accessible emission into the red and near-infrared and making them valuable for sensing in turbid environmental matrices where shorter-wavelength emission suffers from scattering and autofluorescence interference. Their larger exciton binding energies in the nanocrystalline form, arising from stronger quantum confinement at equivalent sizes, produce enhanced sensitivity to surface chemical perturbations and make them particularly effective in heavy metal ion sensing through trap-state quenching mechanisms.

The critical limitation of cadmium chalcogenide QDs for environmental sensing is toxicological: cadmium has been classified as a carcinogen since 2012 and is regulated under the European directive RoHS 2002/95/EC, creating an inherent contradiction in the deployment of Cd-based QDs for environmental monitoring [66]. This has driven sustained interest in zinc chalcogenide (ZnS, ZnSe) QDs as Cd-free alternatives, though these materials exhibit smaller exciton Bohr radii, wider bandgaps confined to the UV–blue spectral region, and generally lower quantum yields than their cadmium counterparts, limiting their application range. The toxicological constraint ultimately motivates the exploration of the less toxic QD families discussed in the following subsections.

### 3.3. Perovskite QDs (PQDs) (CsPbX_3_ and Related Systems)

All-inorganic caesium lead halide perovskite quantum dots (CsPbX_3_, X = Cl, Br, I, Figure 3c) have emerged as one of the most exciting QD families in the past decade, distinguished by a combination of excitonic properties that are genuinely exceptional compared to other QD families. CsPbX_3_ nanocrystals exhibit PLQYs of up to 90%, narrow emission linewidths, and tunable bandgap energies spanning the entire visible spectrum [20]. These properties are a direct consequence of PQDs’ excitonic character, which differs fundamentally from that of chalcogenide QDs.

Quantum confinement in sub-10 nm CsPbX_3_ nanocrystals induces pronounced blue shifts in optical spectra, modifies carrier effective masses, and enhances excitonic binding, while the soft ionic lattice and strong spin–orbit coupling inherent to Pb and Sn atoms impart unique electronic flexibility [67]. Unlike conventional covalent semiconductors, PQDs exhibit remarkable defect tolerance. Defect states tend to remain shallow due to delocalised electronic wavefunctions and high dielectric screening, and DFT simulations have elucidated how the nature and spatial distribution of these defects dictate radiative versus non-radiative recombination pathways, providing a microscopic rationale for the high PLQY observed experimentally even in imperfect nanocrystals [67]. Exciton binding energies in PQDs (100–150 meV) greatly exceed thermal energies at room temperature, ensuring dominant excitonic emission [67], which is consistent with independent measurements in the literature placing the binding energy of CsPbBr_3_ above 40 meV [68].

Compositional tunability through halide substitution provides a uniquely powerful handle on excitonic properties in PQDs. The confinement energy for CsPbX_3_ nanocrystals provides an estimate for the blue shift of the emission peak and absorption edge, with full compositional tunability from approximately 420 nm (CsPbCl_3_) to 700 nm (CsPbI_3_) achieved through halide alloying [20]. For sensing applications, this enables spectral matching of QD emission to the absorption band of a target analyte for FRET-based detection with a degree of control not achievable in other QD families. Molecular organic framework (MOF) encapsulation of CsPbBr_3_ QDs has demonstrated luminescence maintenance for over 30 months under ambient conditions and several hours underwater, a stability performance that substantially exceeds what isolated PQDs typically achieve and that is prerequisite for environmental deployment [69].

The principal limitation of CsPbX_3_ PQDs for environmental sensing is chemical instability toward water, humidity, and polar analytes. The ionic perovskite lattice is susceptible to dissolution and phase transformation under aqueous conditions, and halide ion exchange with environmental anions can alter optical properties in ways indistinguishable from analyte-induced signals. Lead toxicity also presents an environmental deployment challenge analogous to that faced by cadmium-based QDs, driving research into lead-free perovskite alternatives such as Cs_2_AgBiX_6_ double perovskites, bismuth halide systems, and tin perovskites, though these currently exhibit inferior optical properties [21].

### 3.4. III–V QDs (InP, InAs, GaP)

III–V quantum dots, most prominently indium phosphide (InP), have emerged as the leading non-toxic alternative to cadmium-based QDs for applications where regulatory compliance and environmental safety are paramount. InP QDs possess an exciton Bohr radius of approximately 10–11.3 nm and a narrow bulk bandgap of 1.35 eV, offering tunability throughout the visible to NIR spectrum [66,70]. Compared to CdSe QDs with a bulk bandgap of 1.74 eV, the smaller bandgap of InP extends the emission range to NIR wavelengths, a significant advantage for sensing in turbid environmental matrices where visible emission is scattered and autofluorescence is problematic [71]. InP-based QDs have the advantages of a large exciton Bohr radius, high absorption coefficient, wide wavelength tunability, and low toxicity, properties that position them as the most promising type of non-toxic QD [70]. Emission wavelengths from 480 to 845 nm have been achieved by varying core size and shell thickness in InP/ZnSe/ZnS core/shell/shell structures, with FWHM values consistent with those of analogous cadmium chalcogenide systems [72]. For sensing applications, this multishell architecture provides both the photostability and emission linewidth needed for quantitative analytical measurements while avoiding the regulatory constraints that limit cadmium- and lead-based systems.

The radiative exciton decay lifetime in InP QDs and its dependence on shell architecture differ from those of CdSe QDs due to the higher degree of quantum confinement in InP systems of equivalent physical size [73]. A key synthetic challenge for InP QDs is the management of surface chemistry: the covalent character of the InP lattice makes surface defect passivation more chemically demanding than for the more ionically bonded chalcogenide QDs, and bare InP QDs are prone to oxidation and surface etching that introduces broad deep trap emission competing with band-edge fluorescence. The development of multishell architectures, particularly InP/ZnSe/ZnS, has substantially mitigated these issues [72]. The near-blinking-free behaviour observed in InP/ZnSe QDs, attributed to an Auger blockade mechanism, is a significant practical advantage for sensing: stable, non-intermittent emission simplifies signal averaging and improves detection reliability in single-particle and low-concentration sensing regimes.

### 3.5. Comparative Excitonic Properties: A Cross-Family Perspective

The four QD families surveyed above differ not merely in their emission wavelengths and quantum yields, but in the fundamental character of their excitonic states, the very states through which all sensing transduction occurs. Table 1 summarises key excitonic parameters across families, which collectively determine their sensing performance. Two overarching trends emerge from this comparison. First, QD sensing involves a fundamental balance between excitonic stability and excitonic accessibility. Features that enhance emitter performance, including strong exciton binding, high quantum yield, photostability, and effective shell passivation, also reduce the extent to which excitons can be perturbed by surface chemical interactions. Therefore, rational sensor design requires careful optimisation of this balance rather than simply maximising brightness or stability. Second, no single QD family is universally optimal for all pollutant classes; instead, the most suitable platform depends on the specific interplay between excitonic properties, surface chemistry, analyte interactions, and the required sensing mechanism.

## 4. Defects, Dopants, and Surface States as Excitonic Modulators

If the exciton is the analytical signal carrier in a QD-based sensor, then defects, dopants, and surface states are the primary modulators of that carrier, governing its lifetime, spatial extent, binding energy, and ultimately whether it recombines radiatively to produce a useful optical signal or decays non-radiatively into heat. Understanding how these structural imperfections and intentional chemical modifications reshape the excitonic landscape is essential for two reasons: it explains why QD sensors behave as they do, and it provides the rational design vocabulary needed to engineer sensors with superior performance. This section examines each class of excitonic modulator in turn, tracing their effects from the atomic scale to the measurable optical and sensing properties that define analytical utility.

### 4.1. Intrinsic Defects

**Figure 4:**
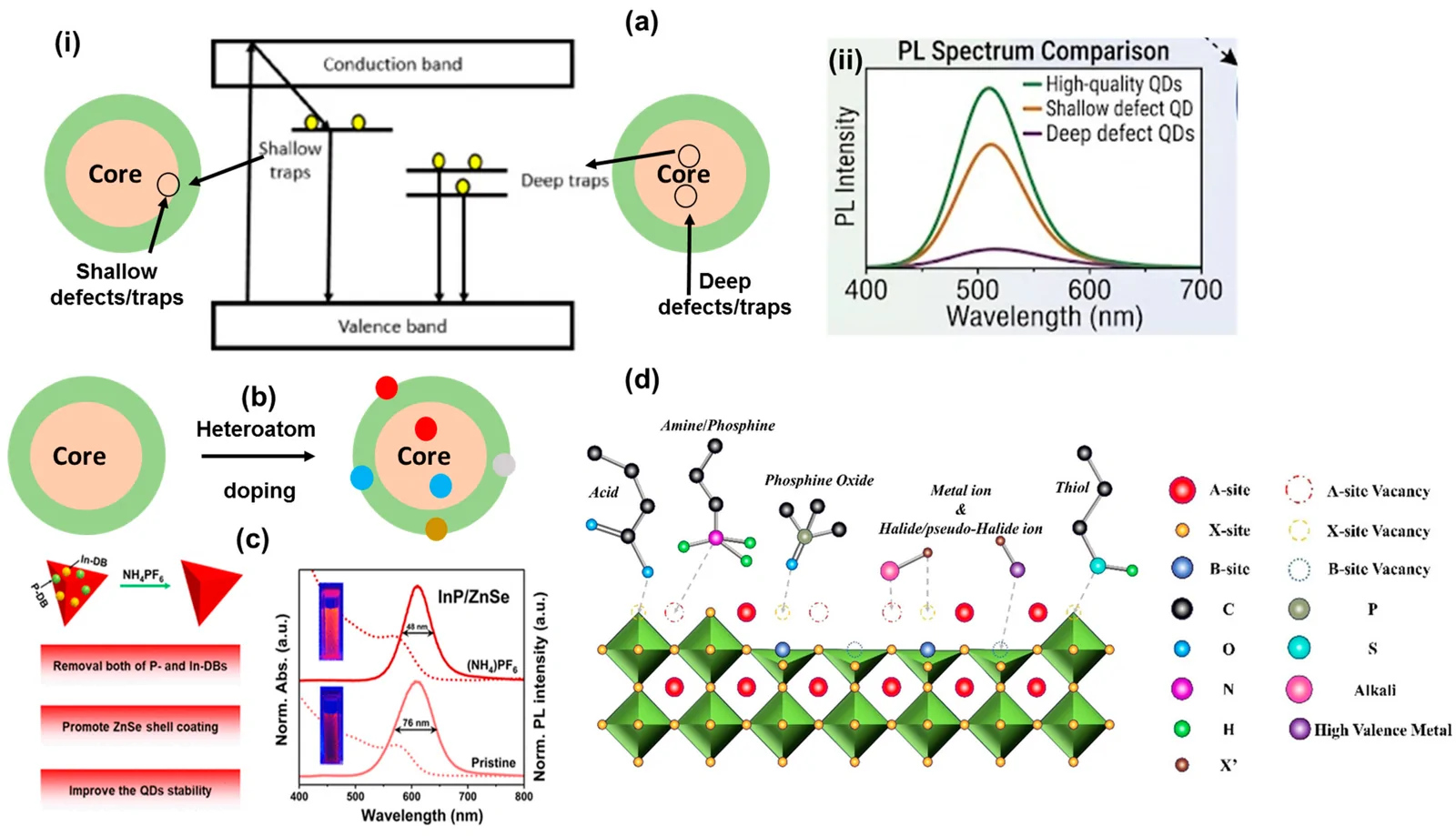
Defect states and surface engineering in quantum dots (QDs). (**a**) Schematic illustration of shallow and deep defect/trap states in QDs, showing defect energy levels located near the band edges (shallow traps) or within the bandgap (deep traps), together with their effects on exciton recombination pathways; (**i**) band-edge representation of shallow and deep traps and (**ii**) corresponding photoluminescence (PL) spectra, illustrating the moderate impact of shallow defects and the strong quenching effect of deep defects on radiative emission. (**b**) Representative heteroatom-doped CQD structure highlighting the role of dopant-induced defect states in tailoring optical properties. (**c**) Surface passivation strategy for InP-based QDs, showing simultaneous passivation of In and P dangling bonds and promotion of ZnSe shell growth, resulting in enhanced PL efficiency and stability (adapted from [81]). (**d**) Schematic representation of common surface ligands, defect sites, and passivation mechanisms in perovskite quantum dots (adapted from [82]). Together, these examples illustrate how defect depth, density, and surface chemistry govern excitonic dynamics and optical performance in QD systems.

All real QDs contain defects arising from thermodynamic limitations during synthesis, surface reconstruction, and environmental exposure. These intrinsic defects are broadly classified as shallow defects (shallow traps), whose energy levels lie near the conduction or valence band edges [76], and deep defects (deep traps), whose states are located within the bandgap and act as efficient non-radiative recombination centres [77] (Figure 4a(i)). Shallow defects only moderately perturb exciton dynamics because trapped excitons can be thermally released and subsequently recombine radiatively. Consequently, quantum yield remains close to unity and decreases only slightly with increasing defect reorganisation energy [78]. In contrast, deep defects strongly promote non-radiative decay, causing rapid exciton relaxation through in-gap states and reducing quantum yield toward zero [78] (Figure 4a(ii)). Defect-induced polaron shifts and exciton–phonon coupling further influence the competition between radiative emission and non-radiative recombination [78]. The nature and abundance of intrinsic defects depend strongly on QD composition. In CdSe QDs, Cd-dimer and Se defects create shallow and deep in-gap states that govern photoluminescence decay dynamics, localise excitonic orbitals, and generate large dipoles responsible for multiexponential PL decay [79]. Perovskite QDs such as CsPbBr_3_ are generally defect-tolerant, possessing largely benign defect states; however, deep traps beyond the conduction band minimum still exist and can influence sensing performance [80]. For CQDs, the distinction between defects and emissive surface states is less clear. Defects associated with sp^2^/sp^3^ carbon domains and heteroatom dopants (N, B, P, and S) create electronic states that contribute directly to fluorescence emission [56]. Unlike chalcogenide QDs, where defects are typically detrimental and require passivation, many CQD sensing mechanisms rely on these surface states. Consequently, controlling their depth, density, and chemical nature is a central strategy in CQD sensor design.

### 4.2. Heteroatom Doping

The deliberate introduction of foreign atoms into the QD lattice (Figure 4b), i.e., doping, represents the most powerful synthetic handle for expanding the excitonic properties of QDs beyond what is achievable through size and composition control alone. Dopants modify the excitonic landscape through multiple mechanisms: they alter the local dielectric environment, introduce new intra-gap energy levels, shift the positions of the HOMO and LUMO, change the effective mass of charge carriers, and, in some cases, generate entirely new radiative channels [24].

In CQDs, nitrogen doping is the most extensively studied and impactful form of heteroatom modification. The HOMO–LUMO gaps of N-doped graphene QDs differ markedly depending on nitrogen configuration: graphitic N yields a gap of 0.77 eV, pyrrolic N 0.25 eV, and pyridinic N 2.69 eV, with boron, phosphorus, and sulphur co-doping causing additional absorption peaks in the visible range [83]. This configuration specificity means that the spectral position of the excitonic emission is directly controllable through the nitrogen doping geometry, providing a synthetic pathway to emissions spanning the UV through visible range from a single carbon-based material platform. Nitrogen-doped CQDs exhibit the highest absorption and emission intensities among heteroatom-doped systems, with doping significantly altering the absorption and emission properties in ways that enhance ion selectivity for sensing applications [84]. The combination of nitrogen and boron co-doping creates donor-acceptor pairs within the carbon matrix that can dramatically increase quantum yields through intramolecular charge transfer excitation pathways not available in single-element-doped systems, with B,N-CQDs demonstrating high sensitivity as fluorescence sensors for Cr^3+^, Cu^2+^, and Fe^2+^ metal ion detection [85].

For chalcogenide QDs, transition metal doping, particularly with manganese (Mn^2+^), copper (Cu^+^/Cu^2+^), and silver (Ag^+^), introduces dopant-centred emission channels that are spectrally distinct from band-edge excitonic emission. The Mn^2+ 4^T_1_→^6^A_1_ transition produces a characteristic orange emission around 585–600 nm that is size-independent and therefore provides a built-in internal reference wavelength, a property directly exploited in ratiometric sensing architectures [86,87]. For perovskite QDs, B-site doping with Mn^2+^ has been demonstrated to deliver dual-function sensing probes for copper detection and temperature sensing through a combination of excitonic and dopant emission channels, with the Mn^2+ 4^T_1_(^4^G)→^6^A_1_(^6^S) transition producing a slight red shift in emission that provides a spectrally resolved second channel [88]. Nitrogen-doped CQDs derived from sustainable precursors have shown red-shifted absorption due to the incorporation of pyrrolic and graphitic nitrogen, which enhances electronic conjugation and introduces new electronic transitions that extend the sensing window toward longer wavelengths [89].

### 4.3. Surface States

The surface of a QD is not merely a geometric boundary; it is an excitonic interface. Because QD dimensions are comparable to the exciton Bohr radius, a substantial fraction of the exciton wavefunction probability density is located at or near the particle surface, where it is accessible to perturbation by adsorbed molecules, surface functional groups, and the surrounding medium. The density and character of surface states therefore directly determine how sensitively, and through what mechanism, an analyte interaction is transduced into a change in PL [35].

Surface traps are ubiquitous in nanoscopic semiconductor materials. In these conditions, the formation of shallow or deep mid-gap states as surface traps that provide pathways for nonradiative exciton recombination is highly likely, which is detrimental for QD-based optoelectronic and sensing applications [35]. A critical insight for sensing design is that surface states create a bidirectional excitonic modulation opportunity (i.e., eversible, two-way control (e.g., redshift/blueshift or enhancement/quenching) of excitons) [90]. Analytes that introduce new surface traps, most commonly metal ions that coordinate to surface anion sites and create additional non-radiative pathways, quench emission and produce turn-off sensors [91]. Analytes that passivate existing surface traps by coordinating to dangling bond sites and removing them from the non-radiative recombination network enhance emission and produce turn-on sensors [81,92]. The importance of surface passivation is exemplified by Zhe [81], who developed a one-step surface peeling and passivation strategy for InP QDs that simultaneously passivated In and P dangling bonds and facilitated ZnSe shell growth (Figure 3c). The resulting InP/ZnSe QDs exhibited enhanced PLQY (~70%), narrower emission linewidths, and improved stability, highlighting how targeted surface-state engineering can suppress non-radiative recombination and strengthen excitonic emission. This bidirectionality, quantified through atomistic simulations revealing how defect-induced polaron shifts and exciton-phonon couplings govern the balance between efficient radiative emission and rapid nonradiative decay [78], makes surface-state engineering one of the most versatile tools in the sensor designer’s toolkit.

In CQDs, surface state emission and its modulation by analytes are particularly rich. The surface state represents a synergistic hybridisation of carbon backbones and connected functional groups within a CQD, and the energy gap of surface states correlates with the extent of the π-electron system and surface chemistry [57]. Those surface defect sites serve as capture centres for excitons, giving rise to surface defect state-related fluorescence whose spectral position and intensity are directly responsive to the chemical identity of species adsorbed at the surface. The gap between the HOMO and the LUMO narrows with increasing surface oxidisation, allowing a decrease in PL energy to obtain emission at higher wavelengths [93]. This mechanism is directly relevant to sensing polar analytes that modify the surface oxidation state of CQDs upon binding.

### 4.4. Ligand Chemistry

The organic or inorganic ligands that cap the surface of colloidal QDs play a dual role: they govern both the photophysical quality of the QD (by passivating or creating surface states) and the accessibility of the excitonic core to analyte interactions (by controlling what can reach the surface and how). Ligands can act as efficient moderators of exciton relaxation by affecting phonon-assisted intraband relaxation, Auger recombination, and energy transfer to ligand vibrations; the PLQY of QDs is greatly determined by the competition between radiative recombination of band-edge excitons and electron or hole-trapping processes [94]. The donor-acceptor character of a ligand determines which type of surface trap it passivates. The use of σ-donating ligands such as alkylamines can enhance the PLQY of CdSe QDs by up to 50% through effective passivation of electron traps, thus resulting in weakened non-radiative recombination of excitons; thiolates are both σ- and π-donating ligands that can effectively passivate electron traps but simultaneously introduce hole traps [94]. This selectivity means that ligand choice is not a neutral decision; the same ligand that improves quantum yield by passivating one trap type may inadvertently create a new trap of the opposite character, shifting the dominant quenching mechanism and altering the sensor’s response profile.

For perovskite QDs specifically (Figure 4d), the polarity, conductivity, stability, and interaction effects of ligands with QD surfaces create complicated ligand–QD relationships that greatly influence the successful synthesis of QDs and the performance of QD-based devices [82]. The labile binding of oleic acid and oleylamine to the ionic perovskite surface means that ligands are continuously exchanging between bound and free states in solution, creating dynamic fluctuations in surface trap density. This passivation mechanism is critically important for sensing: understanding ligands based on their functional groups provides design principles for PQD surface ligands applicable to most sensing scenarios [82]. For sensing applications, this complexity implies that the choice of capping ligand should be informed not only by colloidal stability and biocompatibility requirements but by an explicit analysis of which surface trap types the ligand will create or passivate, and how analyte coordination at residual surface sites will shift the trap balance.

### 4.5. The Defect–Exciton Energy Map as a Sensing Design Tool

**Figure 5:**
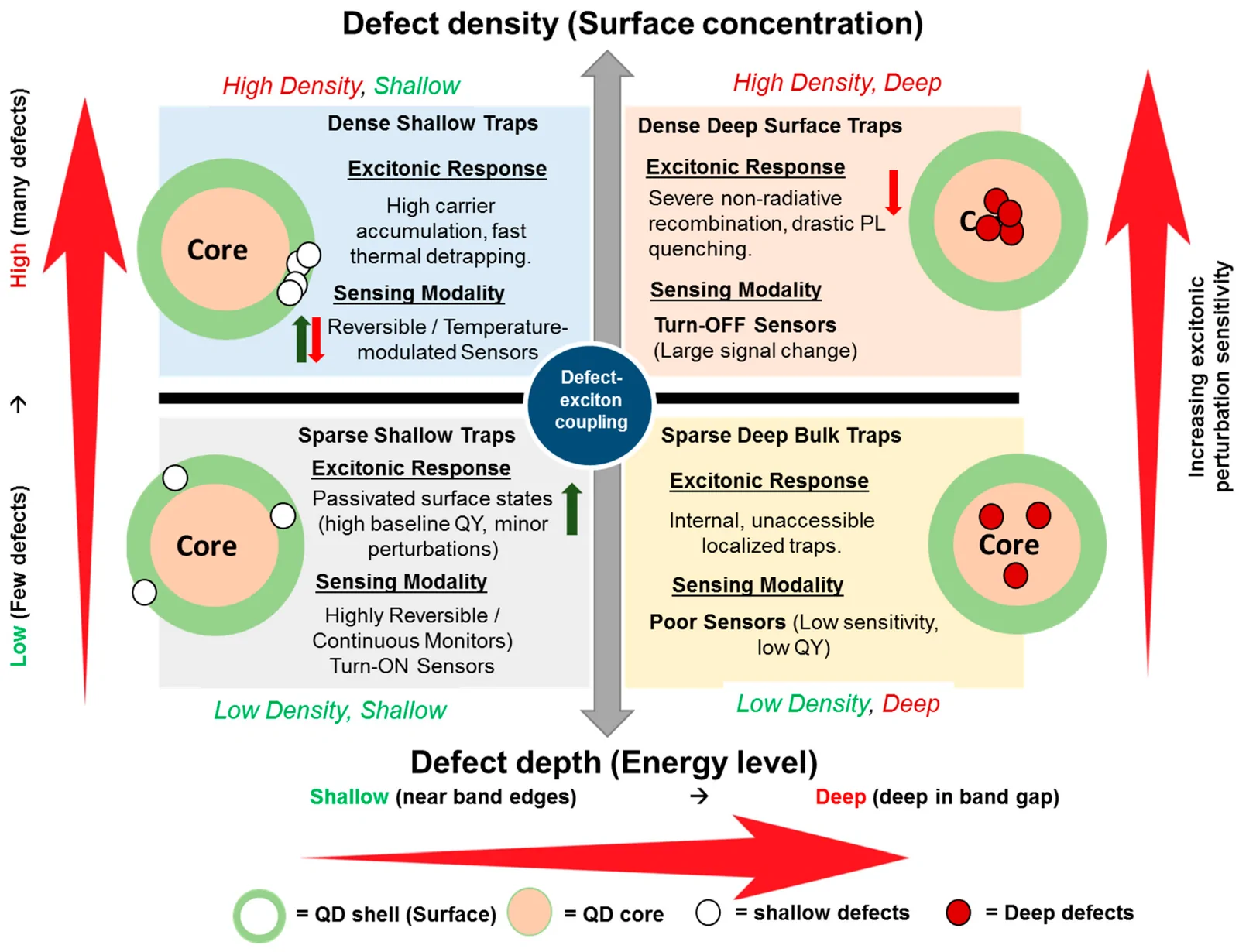
Defect–Exciton Energy Map illustrating how defect density, defect depth, and spatial accessibility determine excitonic behaviour and sensing response in QDs. Shallow defects near the band edges can enable reversible exciton modulation, while deep surface traps promote non-radiative recombination and fluorescence quenching. Sparse accessible defects favour high quantum yield and turn-on sensing through analyte-induced passivation, whereas dense deep traps produce strong excitonic perturbation and turn-off responses. In contrast, deep bulk defects remain largely inaccessible and contribute limited sensing functionality. The framework highlights defect engineering as a key strategy for controlling exciton dynamics and designing pollutant-responsive QD sensors.

The relationships established throughout this section can be integrated into a conceptual framework, the Defect–Exciton Energy Map (Figure 5) which links defect characteristics to excitonic and optical behaviour and, ultimately, to sensing performance. Rather than treating defects as uniformly detrimental or uniformly beneficial, this framework illustrates how the density, depth, and spatial accessibility of defect states collectively determine whether excitons undergo productive modulation or irreversible deactivation. The key design parameters are the energetic position of trap states relative to the band edges (shallow versus deep), their location within the QD (surface versus bulk), their chemical nature (electron- or hole-trapping character), and their population density. Different combinations of these parameters generate four distinct excitonic response regimes, each of which corresponds directly to one or more of the transduction mechanisms described in Section 5. In the sparse shallow trap regime (lower left quadrant, Figure 4), defect states lying close to the band edges at low surface concentration produce passivated surface states and high baseline quantum yield. The exciton is predominantly radiative; reversible trapping and thermally activated detrapping maintain controlled signal modulation, and the sensing modality that follows is turn-on detection, where analyte-induced further passivation of residual surface traps produces a measurable PL enhancement or highly reversible continuous monitoring. FRET operates most efficiently in this regime because competing non-radiative pathways are minimised, maximising the baseline exciton lifetime, and therefore, the window for donor-to-acceptor energy transfer. In the dense shallow trap regime (upper left quadrant), high concentrations of shallow defects produce elevated carrier accumulation, fast thermal detrapping, and strongly temperature-modulated emission. The dominant sensing mechanism in this regime is dynamic quenching, characterised by lifetime shortening detectable by time-resolved photoluminescence (TRPL) and a Stern–Volmer response whose slope increases with temperature, diagnostic signatures that distinguish this regime from static quenching operating in the deep trap quadrants. In the dense deep surface trap regime (upper-right quadrant), deep mid-gap states at high surface density introduce efficient non-radiative pathways, resulting in severe photoluminescence quenching and large analyte-induced signal changes. This is the optimal regime for turn-off sensors: for analytes such as heavy metal ions that introduce new deep trap states through cation exchange or surface coordination, the large per-binding-event signal change produces high analytical sensitivity. For electron-deficient analytes with appropriate redox potentials, photoinduced electron transfer (PET) operates alongside trap-state quenching, further amplifying the response. ECL-based sensing also benefits from this regime, since high-density surface-accessible defects facilitate charge injection at the electrode-QD interface and generate electrically driven excitons with high efficiency. In the sparse deep bulk trap regime (lower right quadrant), deep defects located within the QD core rather than at the accessible surface produce internal, inaccessible localised traps. These defects quench emission without interacting productively with surface analytes, resulting simultaneously in low quantum yield and low sensing sensitivity, the least useful combination for analytical applications, and the primary target of synthetic defect suppression strategies, including multishell passivation and halide surface treatment.

This map provides a rational basis for designing QD sensors by balancing excitonic stability with chemical responsiveness: optimal sensing requires defects that are sufficiently accessible to interact with analytes while avoiding excessive non-radiative losses that suppress optical output. Critically, the map does not replace the individual mechanism descriptions established in Section 5; FRET, PET, trap-state quenching, static and dynamic quenching, charge transfer, and lifetime sensing each retain their mechanistic identity, but the map integrates them into a single, navigable design space. Given a target analyte and desired sensing modality, the map specifies which defect regime the QD must occupy, guiding both material selection and surface chemistry design before any experimental work is undertaken. This shift from post hoc mechanism identification to a priori defect-guided design is the framework’s primary contribution to the field.

#### Worked Example: Designing a CQD Sensor for Hg^2+^ Detection

The predictive value of the Defect–Exciton Energy Map is illustrated through a design exercise targeting a CQD-based turn-off sensor for Hg^2+^ in environmental water.

***Target quadrant identification.*** Hg^2+^ detection through trap-state creation requires coordination to accessible surface anion sites and the introduction of deep mid-gap states that capture photoexcited carriers corresponding to the Dense Deep Surface Trap regime (upper right quadrant, Figure 5). The starting defect landscape must be sparse-to-moderate in deep surface trap density: sufficient baseline quantum yield to support quantitative measurement, yet surface-accessible enough to respond strongly to Hg^2+^ coordination.

***Material and surface chemistry selection.*** CQDs are the appropriate family because their carbon surface framework provides abundant thiol- and amine-functionalised sites with high affinity for Hg^2+^ through soft-acid/soft-base pairing [10]. The map then predicts the transduction mechanism: Hg^2+^ coordination at thiol sites creates a Hg–S mid-gap state that captures the photogenerated hole before radiative recombination, producing static PL quenching with linear Stern–Volmer behaviour at low concentrations and upward curvature where static and dynamic contributions co-operate [95]. TRPL lifetime shortening proportional to trap-state density provides the definitive experimental signature distinguishing genuine excitonic quenching from an IFE artefact.

***Selectivity prediction.*** The map simultaneously predicts the primary limitation: thiol sites favour Hg^2+^ by the hard-soft acid-base principle, but soft Lewis acids, notably Cu^2+^, will produce cross-sensitivity, requiring a masking agent or recognition layer in complex matrices. This design requirement is explicit before any experiment is conducted.

***Literature validation.*** All predictions are confirmed: thiol-functionalised CQD sensors for Hg^2+^ achieve LODs of 0.1–1 nM, show linear Stern–Volmer responses with TRPL lifetime shortening, and exhibit Cu^2+^ cross-sensitivity mitigated by EDTA masking [96,97], exactly the performance profile specified by the Dense Deep Surface Trap regime. This example illustrates the framework’s core value: the target analyte and desired sensing modality together specify the required defect configuration, converting sensor design from empirical screening into a hypothesis-driven process. The same logic can be extended across all four quadrants and pollutant classes in Section 7, establishing the Defect–Exciton Energy Map as a potential generalisable predictive design tool rather than a post hoc rationalisation.

This framework connects directly to the first-principles work on defect-engineered CQDs [24], where an inverse relationship between exciton binding energy and optical brightness was established across eight defect configurations, and three optical classes were identified, including UV-bright emitters, visible absorbers, and NIR-active systems, each with distinct excitonic characters arising from specific defect configurations. The Defect–Exciton Energy Map generalises this result across all QD families: it positions defect engineering not as a synthetic afterthought but as the primary rational design lever for tailoring excitonic response to specific pollutant detection challenges.

## 5. Excitonic and Optical Transduction Mechanisms for Pollutant Sensing

Having established how excitonic properties are governed by quantum confinement, fine structure, phonon interactions, and defect states, the next critical question is how these excitonic perturbations are translated into measurable sensing signals. In QD-based sensors, pollutant detection does not arise directly from analyte recognition but from analyte-induced modifications of exciton dynamics rather than from analyte recognition alone, the exciton serves as the central transduction element linking molecular interactions at the QD interface to observable optical responses. Before describing individual pathways, an important distinction must be drawn. Not all apparent fluorescence changes in QD-based sensors originate from genuine excitonic perturbation. As introduced in Section 1, three categories of signal-generating processes must be separated: genuine excitonic transduction, in which the analyte directly modifies the excitonic state; non-excitonic optical phenomena, principally the inner filter effect (IFE) and light scattering, which produce fluorescence changes without perturbing the exciton such as certain electrochemiluminescence pathways, where the excitonic character of the emitting state depends on the specific coreactant mechanism. The taxonomy developed in this section applies rigorously to the first category, explicitly identifies the second as requiring optical correction rather than mechanistic interpretation, and qualifies the third where relevant. The individual mechanisms described below include photoinduced electron transfer, FRET, static and dynamic quenching, ratiometric sensing, charge-transfer exciton formation, lifetime-based detection, and ECL.

### 5.1. Photoluminescence (PL) Quenching

**Figure 6:**
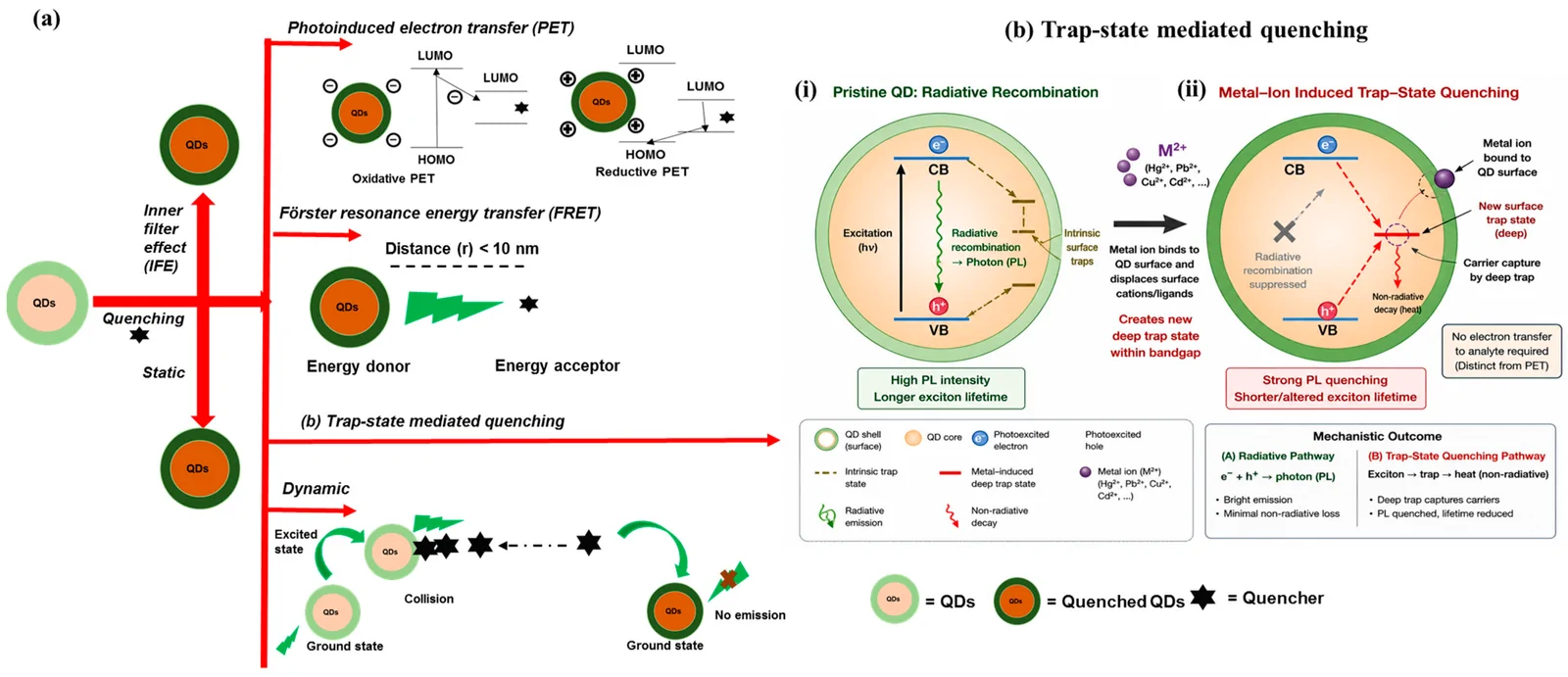
Excitonic quenching mechanisms in fluorescent quantum dots for analyte sensing. (**a**) Overview of major fluorescence quenching pathways, including static quenching, dynamic quenching, inner filter effect (IFE), Förster resonance energy transfer (FRET), photoinduced electron transfer (PET), and trap-state-mediated quenching. (**b**) Schematic illustration of trap-state-mediated quenching: (**i**) pristine QDs exhibit radiative exciton recombination with minimal non-radiative losses, while (**ii**) analyte-induced surface defect formation (e.g., metal-ion coordination or surface cation displacement) introduces deep trap states within the bandgap that capture photogenerated carriers, suppress radiative recombination, and accelerate non-radiative decay. This process results in reduced photoluminescence intensity and shortened exciton lifetime, distinguishing trap-mediated quenching from PET-based mechanisms.

PL quenching is the reduction in QD emission intensity and, upon analyte exposure, is by far the most widely reported sensing modality in the QD environmental sensing literature. Despite its apparent simplicity, PL quenching encompasses mechanistically distinct sub-processes that must be distinguished to understand why a given QD-analyte system responds as it does. Fluorescence sensing technology based on QD probes can be realised through sensing mechanisms such as fluorescence/dynamic quenching, photoinduced electron transfer (PET), trap-state-mediated quenching, and fluorescence resonance energy transfer (FRET) (Figure 6a), as well as non-excitonic optical phenomena, including the inner filter effect (IFE), which are discussed separately in Section 5.1.4. The dominant mechanism determines the LOD achievable and the susceptibility to matrix interference [98,99].

#### 5.1.1. Static vs. Dynamic Quenching

The Stern–Volmer equation (Equation (3)) quantifies the reduction in fluorescence intensity upon addition of quencher molecules, with the underlying model based on the formation of an adduct between the quencher and the excited state (dynamic quenching) or the ground state (static quenching) fluorophore at steady-state conditions [95].

(3) $$\frac{I_{0}}{I}=1+k_{SV}[Q]$$

where *I*_0_ and *I* are the fluorescence intensities of QDs in the absence and presence of the quencher *Q*, respectively, and *K_SV_* is the Stern–Volmer quenching constant.

Static quenching arises when an analyte forms a non-fluorescent ground-state complex with the QD, a complex that is already dark before photoexcitation and therefore contributes no emission even at low analyte concentrations. Dynamic (or collisional) quenching occurs when a freely diffusing analyte molecule collides with an excited QD during its excited-state lifetime, deactivating the exciton non-radiatively [91,95]. The two mechanisms are experimentally distinguished by lifetime measurement: static quenching reduces PL intensity without changing the exciton lifetime of the remaining emitters, whereas dynamic quenching shortens the lifetime of the full QD population proportionally. The IFE, by contrast, reduces apparent PL intensity without affecting the lifetime at all, making TRPL the definitive three-way discriminator among static quenching, dynamic quenching, and IFE [27,100]. Upward deviations from linearity in the Stern–Volmer plot signal the simultaneous operation of both mechanisms, a situation common in complex environmental matrices where analytes interact with QDs through both specific surface coordination and diffusional collisions.

#### 5.1.2. Photoinduced Electron Transfer (PET)

PET is a quenching mechanism specific to analytes with suitable redox potentials relative to the QD conduction or valence band. Depending on the direction of the electron transfer process, PET can be classified into oxidative electron transfer (QDs act as the electron donor, with the electron moving from the LUMO of the QDs to the LUMO of the quencher) and reductive electron transfer (electron moves from the LUMO of the quencher to the HOMO of the QDs) (Figure 6), either case usually resulting in decreased fluorescence [98,101]. Electron-deficient pollutants, particularly nitroaromatic compounds, certain heavy metal ions, and oxidising agrochemicals, are effective PET acceptors. PET efficiency depends critically on the energy offset between QD and analyte orbital levels and on the spatial separation between the exciton and the analyte, dependencies that provide inherent selectivity since only analytes whose orbital energies fall within the PET-active window produce a response [101]. PET is distinguished from trap-state-mediated quenching by its dependence on analyte redox potential and its reversibility upon analyte removal; trap-state quenching, by contrast, may be irreversible if the metal ion undergoes lattice incorporation.

#### 5.1.3. Trap-State-Mediated Quenching

Many environmental analytes, particularly heavy metal ions such as Hg^2+^, Pb^2+^, Cu^2+^, and Cd^2+^, quench QD emission through the creation of new non-radiative trap states at the QD surface, rather than through PET (Figure 6b). When a metal ion displaces a surface cation and introduces a new electronic state within the QD bandgap, deep trap states capture the photoexcited hole or electron before radiative recombination can occur, producing efficient quenching even at sub-nanomolar analyte concentrations. This mechanism is distinct from PET in that it does not require the analyte to remain in solution, and it produces measurable changes in both PL intensity and exciton lifetime [78]. The lifetime shortening produced by trap-state creation is the definitive experimental signature distinguishing this mechanism from the IFE (no lifetime change) and static quenching (no lifetime change in remaining emitters). This distinction is practically important: reporting trap-state quenching without TRPL confirmation conflates three mechanistically and analytically distinct processes.

#### 5.1.4. The Inner Filter Effect (IFE): A Critical Methodological Distinction

The IFE is classified in this review as a non-excitonic optical phenomenon rather than an excitonic transduction mechanism, because it operates without any perturbation of the QD excitonic state. The IFE is frequently misidentified as true excitonic quenching in the QD sensing literature and warrants explicit discussion. In the IFE (Figure 6), an analyte absorbs the excitation light before it reaches the QD (primary IFE) or absorbs the QD emission before it reaches the detector (secondary IFE), producing an apparent reduction in fluorescence intensity that has nothing to do with excitonic perturbation [27,102]. The IFE is not an intrinsic property of the QD-analyte interaction; it operates through trivial optical absorption and therefore carries no information about binding specificity or the excitonic state [102]. Critically, IFE contributions can be mathematically corrected using UV-Vis absorption spectra of the analyte, and modern Z-position correction methods require only two fluorescence measurements at different vertical axis positions of the optical element to achieve a linear fluorescence response over ~98% of the concentration range [27]. In perovskite sensing systems specifically, the IFE has been shown to be a boon rather than merely a confound [103]. This can be exploited as the primary sensing mechanism when the analyte’s absorption properties are sufficiently distinctive and stable, with PQD-IFE systems achieving higher sensitivity levels than systems relying solely on direct QD-analyte interaction [103]. When the IFE is the operative mechanism, this should be stated explicitly, and the response should not be described as evidence of QD-analyte excitonic interaction.

The clearest experimental test for distinguishing the IFE from genuine excitonic or energy-transfer mechanisms is the presence or absence of sensitised acceptor emission: the IFE cannot produce acceptor sensitisation, whereas FRET-based and charge-transfer-based mechanisms can, and this distinction is most clearly demonstrated in systems where donor and acceptor emissions are spectrally resolved, as in the CD–N-I FRET nanoprobe for peroxynitrite detection [104].

### 5.2. PL Enhancement

Turn-on sensing, where analyte binding results in enhanced QD emission, represents one of the most selective excitonic transduction strategies because fluorescence enhancement requires a specific positive interaction that improves radiative recombination efficiency, rather than the broad range of processes that can induce emission loss.

Surface trap passivation

**Figure 7:**
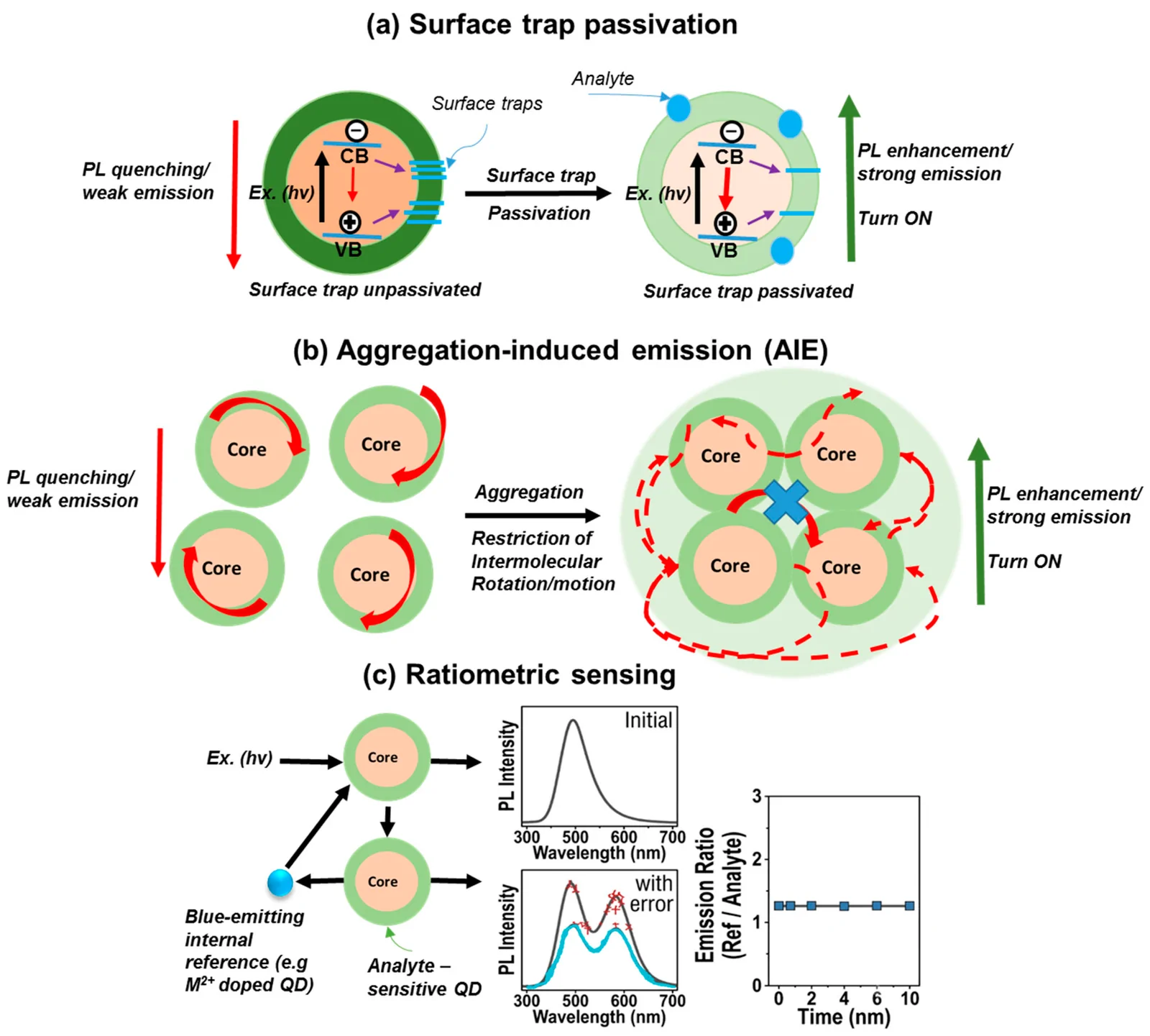
Signal amplification and ratiometric transduction pathways for QD excitonic sensors. (**a**) Turn-on sensing via analyte-mediated surface trap passivation, mitigating non-radiative carrier trapping. (**b**) Aggregation-induced emission (AIE) driven by the restriction of intermolecular rotation/motion, transforming weak emitters into strong radiative structures upon target interaction. (**c**) Self-calibrating ratiometric sensing paradigm utilising a dual-peak emission profile (internal reference vs. analyte-responsive core) to eliminate environmental, concentration, and instrumental errors.

A major mechanism underlying PL enhancement is analyte-induced surface trap passivation [35]. When an analyte coordinates with surface dangling bonds or defect sites responsible for non-radiative recombination, trapped excitons can be redirected toward radiative decay, resulting in increased quantum yield and PL intensity (Figure 7a). The enhancement is often proportional to analyte concentration until available trap sites become saturated, after which further analyte addition produces minimal additional signal. This self-limiting behaviour defines the upper boundary of the linear dynamic range and is an intrinsic feature of passivation-based turn-on sensors that should be accounted for in calibration design. This mechanism is particularly effective for analytes, such as certain metal ions, that interact with surface anion sites without generating new deep trap states.

Aggregation-induced emission (AIE)

A second enhancement pathway involves aggregation-induced emission (AIE), particularly in engineered CQDs [60,105] (Figure 7b). During aggregation, restricted molecular and surface-state motions suppress non-radiative vibrational relaxation, thereby promoting radiative recombination and increasing PL intensity [60,105]. In CQDs, the balance between aggregation-caused quenching and AIE is governed by two competing processes: inter-dot/molecular excitonic coupling and intra-dot/molecular electron–vibration coupling. The relative contribution of these mechanisms can be tuned through QD size and surface chemistry, determining whether aggregation enhances or suppresses fluorescence [60,105]. It is important to note that AIE in CQDs is mechanistically distinct from the classical AIE observed in organic rotor molecules: in CQDs, the dominant suppression of non-radiative decay arises from restricted surface-state motion and inter-dot excitonic coupling [106] rather than from restriction of phenyl ring rotation [107]. This distinction has practical implications for surface chemistry design: AIE-active CQD sensors require surface functionalisation that promotes controlled aggregation rather than colloidal stability, which may compromise matrix robustness in high-ionic-strength environmental samples [106]. The analytical potential of AIE-based systems has been demonstrated in electrochemiluminescence (ECL) sensing, where a red-emissive sulphur QD-based aptamer sensor achieved a linear detection range of 1.0 × 10^−13^ to 1.0 × 10^−8^ mol/L for malathion with an ultralow detection limit of 0.219 fM, highlighting the ability of AIE mechanisms combined with electrochemical excitation to enable trace-level pollutant detection [108]. Furthermore, ratiometric fluorescence sensing provides an additional advantage by introducing an internal reference signal, improving reliability by compensating for variations in excitation intensity, probe concentration, and measurement conditions [109].

### 5.3. Ratiometric Sensing

Single-channel intensity-based sensors are intrinsically vulnerable to systematic errors from fluctuations in excitation power, detector response, QD concentration, and photobleaching. A ratiometric sensing mechanism involves measuring the ratio of two distinct emission or absorption signals. One signal acts as an internal reference while the other responds to the target analyte (Figure 7c). This eliminates environmental and instrumental variables, yielding highly accurate, self-calibrated results [109]. The sensing mechanisms employed in ratiometric sensors span multiple excitonic pathways, including intramolecular charge transfer (ICT), FRET, chelation-enhanced fluorescence (CHEF), PET, excited-state intramolecular proton transfer (ESIPT), AIE, static quenching, dynamic quenching, and IFE [109], illustrating that ratiometric architecture is a signal readout strategy rather than a transduction mechanism in itself, it can be implemented on top of any of the genuine excitonic mechanisms described in this section, and its self-calibrating advantage applies equally to all of them. Several architectures achieve ratiometric responses. Dual-emission QDs, most commonly realised through metal-doped chalcogenide QDs that exhibit both band-edge excitonic emission and a size-independent dopant emission, provide an intrinsically ratiometric platform where the analyte selectively modulates one emission channel while the other serves as a built-in reference [110]. A critical limitation of ratiometric sensing that is frequently overlooked is that self-calibration corrects for intensity fluctuations arising from instrumental and concentration-related sources but does not inherently correct for the IFE or scattering contributions that affect both emission channels simultaneously, a situation that can arise in complex environmental matrices and must be assessed experimentally.

### 5.4. Förster Resonance Energy Transfer (FRET)

FRET is the radiationless transfer of excitation energy from a photoexcited donor to a ground-state acceptor through long-range dipole–dipole coupling [111]. Its rate depends on the inverse sixth power of the donor-acceptor separation distance (Equation (4)) and on the spectral overlap integral between donor emission and acceptor absorption two parameters that together make FRET an exquisitely sensitive and distance-selective transduction mechanism [112]. FRET is very sensitive to the distance between particles, which should be kept at 5–10 nm, and QDs’ wide absorption and tunable emission bands, which allow controllable overlap of emission and absorption spectra, as well as the large Stokes shift to suppress direct acceptor excitation, make QDs excellent FRET donors [100].

(4) $$k_{FRET}=\frac{1}{\tau_{D}}\;{\left(\frac{R_{0}}{r}\right)}^{6}$$

where τ_D_ is the donor excited-state lifetime in the absence of the acceptor, and R_0_ is the Förster radius; r is the distance between donor and acceptor [98]. In a FRET-based sensor (Figure 6), QDs act as donors, modified molecules or doped ions serve as acceptors, and the analyte acts as a quencher; upon incorporation of the analyte, the FRET between the donor and acceptor is destroyed due to quenching of the QDs [90]. FRET-based technologies have played a significant role in biosensing, used as a “molecular ruler” to measure changes in biomolecular conformations, nucleic acid hybridisation, enzyme activity, and environmental parameters [113]. Typical FRET sensors consist of three elements: a donor molecule, an acceptor molecule, and a linker for their connection, with selection of the fluorophores critically important in the design and functionality of FRET sensors [114]. For environmental sensing of pesticides, antibiotics, and hydrophobic organic pollutants, FRET provides specificity through the molecular recognition element rather than through the QD itself, enabling selective detection in complex matrices where direct QD-analyte interactions would be non-specific.

However, the distinction between genuine FRET and IFE or nonspecific quenching is not always straightforward from steady-state PL data alone, and the experimental criteria for making this distinction deserve explicit statement. Wu [104] provide a clear demonstration of how a well-designed FRET nanoprobe resolves this ambiguity: in the CD–N-I system, the appearance of sensitised acceptor emission at wavelengths distinct from both donor excitation and donor emission, combined with the quenching of donor emission, constitutes unambiguous spectroscopic evidence of through-space energy transfer rather than the IFE, since the IFE cannot produce acceptor sensitisation. Additionally, the excitation spectrum of the sensitised acceptor emission matches the donor absorption rather than the acceptor absorption, confirming that energy arrives at the acceptor via the donor excited state rather than through direct acceptor excitation. These spectroscopic signatures—sensitised acceptor emission, donor quenching, and excitation spectrum mapping—constitute the minimum experimental evidence required to claim FRET as the operative mechanism, and should be standard practice in QD-based FRET sensing studies to prevent misattribution of the IFE or collisional quenching as FRET [104,113].

### 5.5. Exciton Binding Energy and Excitonic Landscape Modulation

This mechanism, described in this section and the one most directly connected to the first-principles excitonic framework established previously in this review, encompasses changes in the energy and spatial character of the excitonic state induced by analyte interactions, rather than changes in its lifetime or transfer efficiency. Three sub-mechanisms contribute.

Dielectric environment perturbation

The E_b_ in a QD depends on the dielectric constant of the surrounding medium through the Coulomb screening term in the Brus equation [115]. When analytes adsorb to the QD surface or alter the local dielectric environment through solvation shell reorganisation, surface charging, or the introduction of polarisable molecular groups, the effective dielectric constant experienced by the confined exciton changes, shifting E_b_ and consequently shifting the absorption and emission wavelengths [116,117]. This produces spectroscopic shifts detectable as ratiometric or spectral shift signals that are insensitive to QD concentration and excitation power fluctuations.

Confinement modulation

Analytes that induce changes in QD surface layers or alter the effective potential barrier at the QD surface through coordinated binding can modify the spatial extent of the exciton wavefunction effectively changing the confinement radius experienced by the exciton and shifting optical transition energies accordingly [118,119,120].

Charge redistribution

Analytes with strong electron donor or acceptor character can redistribute charge at the QD surface without completing a full electron transfer (PET) event, creating a surface dipole that shifts band positions and alters the excitonic transition energy as a Stark-effect-like spectral modification [120]. This partial charge transfer operates at analyte concentrations below the threshold for complete PET quenching, extending the lower detection limit of charge-sensitive sensing approaches [121].

These three sub-mechanisms share an important diagnostic feature: they produce spectral shifts (changes in peak emission or absorption wavelength) as their primary analytical signal, rather than intensity changes. This spectral shift signature distinguishes them from quenching-based mechanisms and from the IFE, which produces no spectral shift in the QD emission peak. Spectral shift-based sensing is therefore inherently more resistant to IFE misattribution than intensity-based approaches.

### 5.6. Charge-Transfer Exciton Formation at the QD–Analyte Interface

When analytes with strong electron donor or acceptor character bind directly to the QD surface, particularly aromatic pollutants with extensive π systems, PFAS compounds with fluorinated electron-withdrawing groups, or strongly coordinating metal complexes, the QD-analyte interaction can generate a new type of excited state: an interfacial charge-transfer (CT) exciton. This quasiparticle, distinct from both the QD’s intrinsic Frenkel-type exciton and from a fully charge-separated state, consists of an electron and hole that are spatially separated across the QD-analyte interface but remain Coulombically bound [35]. CT excitons have longer lifetimes than intrinsic QD excitons, lower emission energies, and different polarisation response properties that make them sensitive reporters of interfacial chemistry. For PFAS detection specifically, where the fluorinated carbon chain creates a strongly electron-withdrawing surface environment, CT exciton formation at the QD-PFAS interface may contribute to the observed quenching and spectral shifts in ways not captured by conventional PET or trap-state models [9]. CT exciton formation is experimentally identified by the appearance of a new, red-shifted emission band with a longer lifetime than the intrinsic QD exciton, distinguishable from trap-state emission by its dependence on analyte concentration and its reversibility upon analyte removal.

### 5.7. Time-Resolved Photoluminescence (Lifetime-Based Sensing)

All of the mechanisms described above produce changes not only in PL intensity but also in the exciton lifetime, i.e., the average time between excitation and radiative recombination. Lifetime-based sensing encodes the analytical signal in this temporal dimension rather than in emission intensity, conferring two major advantages [122]. First, lifetime measurements are independent of QD concentration, excitation source fluctuations, and detector sensitivity drift systematic error sources that limit the accuracy of intensity-based sensors in real environmental matrices. Second, lifetime changes can be directly interpreted in terms of the rate constants of individual excitonic decay pathways, providing mechanistic information that intensity measurements alone cannot deliver. The distinction between static and dynamic quenching is definitively resolved by lifetime measurements: static quenching reduces intensity without changing the lifetime of the remaining emitters, while dynamic quenching shortens the lifetime of all QDs proportionally [95,100]. Importantly, lifetime-based sensing does not eliminate all sources of analytical error: it corrects for intensity-based artefacts but does not correct for IFE contributions to apparent spectral shifts, and it cannot resolve sub-nanosecond trap-capture events without specialised ultrafast detection. Shell architecture mediates a fundamental trade-off between photophysical quality and electrochemical accessibility that applies with equal force to lifetime-based optical sensing [123].

### 5.8. Electrochemiluminescence (ECL)

ECL represents a fundamentally different mode of optical transduction in which the exciton is generated not by photon absorption but by electrochemical reaction at an electrode surface. As discussed in Section 1, ECL pathways fall within the ‘partially excitonic’ category of the tripartite framework: in annihilation-pathway ECL, electrogenerated QD radical cations and anions recombine to form genuine electrogenerated excitons, making the process fully excitonic; in coreactant-pathway ECL, the excitonic character of the emitting state depends on the specific QD, coreactant, and applied potential combination and should not be assumed without mechanistic verification [123]. The general process of electrochemiluminescence emission involves occurrence of redox reactions at the electrode surface, chemical reactions in homogeneous solution, formation of excited state species followed by emission of light; the sensing of analytes is based on the principle that any molecule which can alter via promotion or hinderance of either of the ECL emission steps will ultimately change the output signal, resulting in quantitative detection of the analyte [124]. ECL combines the molecular specificity of excitonic emission with the background-free character of electrochemical detection because excitation is electrical rather than optical; there is no excitation light to scatter or be absorbed by the sample matrix.

Due to the large surface-to-volume ratio and high proportion of surface atoms, it is easy to introduce lattice defects and surface defects in the synthesis process of QDs, generating additional defect-state energy levels; since the bandgap of defect states is generally smaller than that of the band-edge state, luminescence arising from the defect state is sensitive to the environment [123]. This defect-state ECL sensitivity is directly relevant to environmental sensing: engineered ECL biosensors for monitoring heavy metal ions have demonstrated simplicity, utility, and remarkable sensitivity in monitoring residual heavy metal contaminants, with ECL achieving superior signal-to-noise ratios compared to fluorescence through elimination of background excitation interference [125]. An innovative approach uses arginine-modified black phosphorus QDs in which both S_1_ and T_1_ excited states contribute to ECL emission, with the arginine modification passivating surface oxidation defects, modulating excited states, and enhancing ECL efficiency, demonstrating that deliberate surface chemistry engineering of the excitonic manifold translates directly into measurable ECL performance gains [126].

## 6. Analytical Techniques for Probing Excitons and Excitonic Sensing

**Figure 8:**
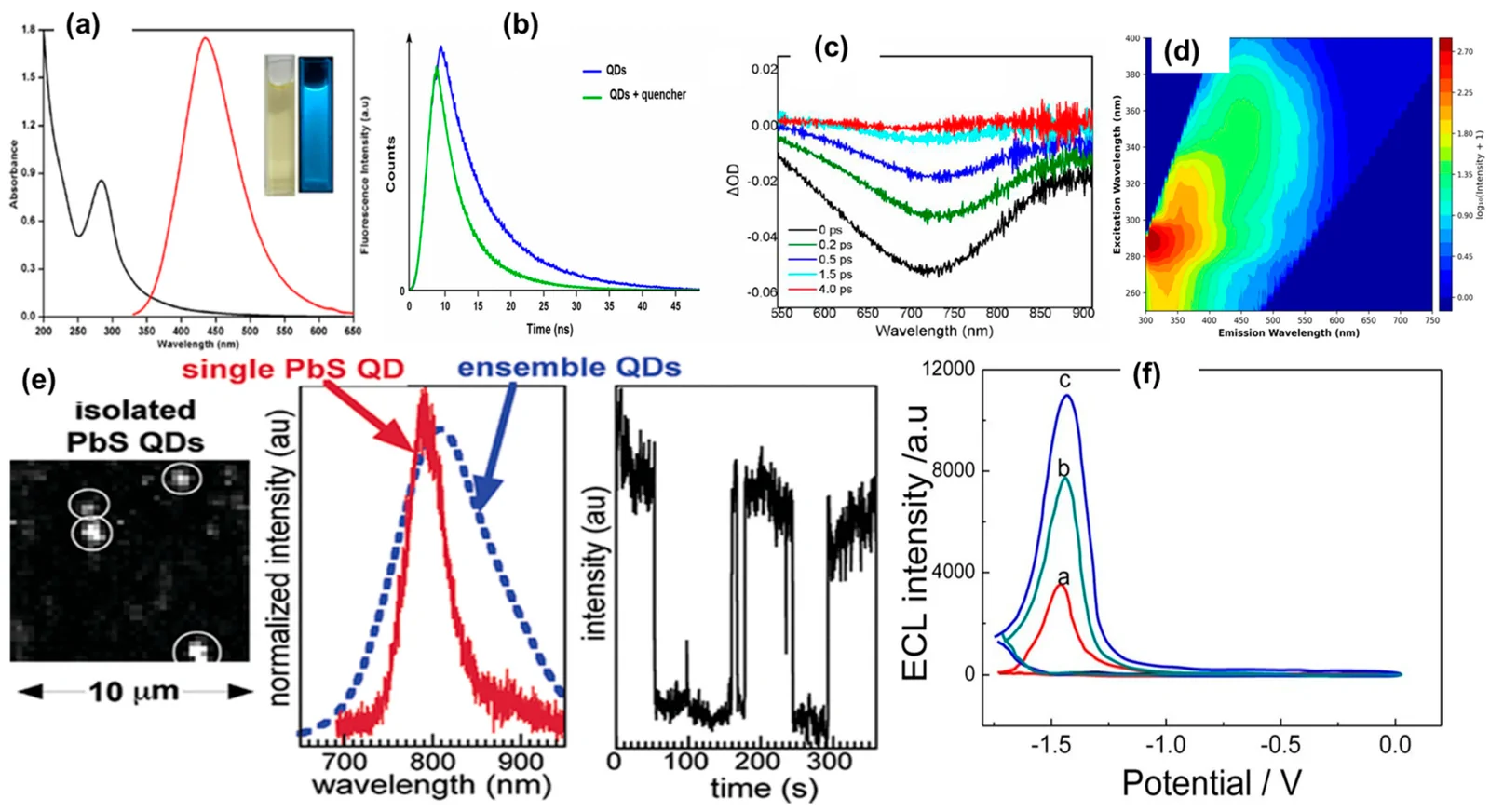
Representative analytical signatures obtained from key techniques used to probe excitonic properties and exciton-mediated sensing mechanisms in QDs. (**a**) Steady-state photoluminescence (PL) and UV–visible absorption spectroscopy revealing excitonic transitions, spectral shifts, and intensity modulation following analyte interaction (adapted from [127]). (**b**) Illustrative time-resolved photoluminescence (TRPL) showing exciton decay dynamics and lifetime variations associated with radiative and non-radiative recombination pathways. (**c**) Transient absorption spectroscopy (TAS) illustrating ultrafast carrier dynamics, including excited-state evolution and charge-transfer processes (adapted from [128]). (**d**) Excitation–emission matrix (EEM) fluorescence mapping demonstrating multidimensional spectral fingerprints for resolving overlapping emissive species and complex sensing responses (adapted from [62]). (**e**) Single-particle spectroscopy highlighting individual QD emission behaviour, blinking dynamics, and exciton heterogeneity beyond ensemble measurements (adapted from [129]). (**f**) Electrochemiluminescence (ECL) measurements demonstrating electrically generated excitonic emission and its potential for highly sensitive sensing applications. Curves a, b, and c represent progressively enhanced ECL emission intensities under increasing experimental conditions, highlighting the tunability of electrically generated luminescence. (adapted from [130]).

Understanding excitonic sensing mechanisms requires analytical approaches capable of linking the observed optical response of QDs with the underlying physicochemical processes responsible for signal generation. These techniques provide access to different dimensions of the excitonic landscape, including energy-level structure, carrier relaxation, defect-mediated recombination, charge transfer, and surface interactions. Consequently, the choice of analytical method determines not only how a sensing response is quantified but also which excitonic transduction pathway can be mechanistically identified and validated. Typical spectra of the different exciton probing techniques are presented in Figure 8.

### 6.1. Steady-State Photoluminescence (PL) and UV–Visible Absorption Spectroscopy

Steady-state photoluminescence (PL) and UV–visible absorption spectroscopy remain the primary tools for evaluating excitonic energy states, optical transitions, and analyte-induced spectral changes in QD sensors. Absorption measurements provide information on ground-state-to-exciton transitions and oscillator strength, reflecting quantum confinement effects described by the Brus model, whereas PL measurements represent the radiative output of the relaxed exciton population. UV-Vis spectroscopy also serves a structural diagnostic role: nitrogen-doped CQDs show red-shifted absorption arising from incorporation of pyrrolic and graphitic nitrogen, which enhances electronic conjugation and introduces new electronic transitions, linking absorption-band position directly to dopant configuration [89]. These approaches have been extensively applied across carbon, chalcogenide, perovskite, and III–V QDs to monitor fluorescence quenching, enhancement, and wavelength shifts caused by analyte interactions. For example, Hg^2+^ and Pd^2+^ detection using CdSe-based QDs has been investigated through PL quenching arising from cation exchange and defect formation, while CQD-based sensors frequently rely on PL enhancement or suppression associated with surface-state modulation [91,131]. In applied sensing, biomass-derived CQDs modified with a deep eutectic solvent have been used in steady-state PL quenching assays to detect Pb^2+^ directly in environmental water samples, with the steady-state response alone sufficient to establish a working calibration for field water analysis [110]. These ensemble-averaged and time-integrated techniques are highly effective for monitoring fluorescence enhancement, quenching, and spectral shifts; however, they cannot independently distinguish between static and dynamic quenching mechanisms or resolve the individual kinetic pathways responsible for the observed response. Despite their widespread use, steady-state PL and UV-Vis measurements carry critical limitations that are frequently underappreciated in the QD sensing literature. Most importantly, they cannot distinguish between static quenching, dynamic quenching, and the inner filter effect (IFE): all three produce apparent reductions in steady-state PL intensity, yet they arise from fundamentally different physical processes with different mechanistic and analytical implications [27]. A study relying solely on steady-state intensity changes cannot determine whether the analyte has genuinely perturbed the excitonic state or merely absorbed the excitation or emission light. This limitation is not merely academic; it directly affects the validity of mechanistic claims and the reliability of LOD calculations, since IFE-based apparent quenching is not specific to the QD–analyte interaction and is therefore susceptible to interference from any co-absorbing matrix component. Additionally, steady-state PL measurements are concentration-dependent, excitation-intensity-dependent, and susceptible to photobleaching-induced drift, all of which introduce systematic errors in quantitative sensing if not corrected. For these reasons, steady-state PL should be considered a screening tool rather than a definitive mechanistic probe: it is necessary but not sufficient for establishing genuine excitonic transduction, and must be complemented by lifetime measurements and IFE correction before mechanistic conclusions are drawn.

### 6.2. Time-Resolved Photoluminescence (TRPL)

Time-resolved photoluminescence (TRPL), commonly measured using time-correlated single-photon counting (TCSPC), provides direct insight into exciton decay dynamics by monitoring emission lifetimes from nanoseconds to microseconds. Unlike steady-state PL, TRPL separates radiative and non-radiative processes, enabling mechanistic interpretation of analyte-induced changes in exciton recombination pathways [42]. TRPL has been widely used to investigate defect-mediated relaxation, trap-assisted recombination, and surface-state interactions in QDs. In defect-rich QDs, multiexponential decay profiles reveal contributions from different emissive and non-emissive states, allowing identification of analyte-induced activation or suppression of specific excitonic pathways [132,133]. Recent studies further demonstrate that lifetime analysis can distinguish surface passivation effects from simple fluorescence intensity enhancement [134], providing a more reliable interpretation of turn-on sensing mechanisms [135,136]. TRPL is also the decisive tool for distinguishing charge-transfer quenching from resonance energy transfer: in graphene-QD hybrid biosensors, PL quenching and its recovery were shown to be driven by charge transfer rather than energy transfer specifically because the fluorescence lifetime remained unchanged upon quenching, a signature that combined with DFT-predicted interfacial charge redistribution enabled femtomolar detection of immunoglobulin G and establishes a template directly transferable to environmental analyte sensing [137]. The critical evaluative value of TRPL in the sensing context lies specifically in the three mechanistic distinctions it can resolve that steady-state measurements cannot. First, it definitively separates static from dynamic quenching: static quenching reduces PL intensity without changing the lifetime of the remaining emitters, whereas dynamic quenching shortens the lifetime of the full QD population proportionally [95]. Second, it distinguishes genuine excitonic quenching from the IFE: the IFE produces no lifetime change whatsoever, so an unchanged TRPL profile in the presence of significant steady-state quenching is definitive evidence of IFE rather than excitonic perturbation [27,138]. Third, it differentiates FRET from charge transfer: FRET shortens the donor lifetime, while charge transfer may or may not change the lifetime depending on whether the transferred carrier returns. These distinctions make TRPL an indispensable validation step for any mechanistic claim in QD-based sensing. Its primary practical limitation is instrumental: TCSPC systems are significantly more expensive and less portable than steady-state fluorimeters, and the nanosecond time resolution of standard TCSPC may be insufficient to resolve the fastest (sub-nanosecond) trap-capture events in high-defect-density QDs, which require streak camera or fluorescence upconversion techniques. For ensemble-level environmental sensing where the goal is quantitative analyte detection rather than mechanistic elucidation, TRPL is not always operationally necessary; but for any publication claiming a specific excitonic mechanism, TRPL data should be considered the minimum standard of mechanistic evidence.

### 6.3. Transient Absorption Spectroscopy (TAS)

Transient absorption spectroscopy (TAS) extends exciton analysis into ultrafast timescales, typically femtoseconds to picoseconds, enabling direct observation of early carrier dynamics following photoexcitation. By monitoring excited-state absorption, stimulated emission, and ground-state bleaching signals, TAS provides information on hot-carrier cooling, exciton formation, Auger recombination, and charge-transfer processes that occur before steady-state emission [133,139]. The combination of TAS and TRPL has been particularly valuable for understanding competing excitonic pathways in semiconductor QDs. For example, studies on colloidal CdSe QDs demonstrated that both techniques resolve multiple decay components associated with radiative and non-radiative relaxation, while spectral analysis across visible and near-infrared regions enables separation of electron and hole contributions [133]. In sensing systems based on charge-transfer excitons, TAS provides direct evidence of ultrafast electron transfer events rather than relying only on the resulting fluorescence changes [140]. Furthermore, a combined steady-state and TAS investigation of CdTe/CdS quantum dots interacting with MoS_2_ nanosheets demonstrated PL quenching of up to 97% at 0.35 mg/mL MoS_2_, with the appearance of a MoS_2_ ground-state bleaching feature in the transient absorption spectrum providing direct spectroscopic evidence that quenching proceeds via electron injection from the QD into the MoS_2_ band edge, rather than via simple energy transfer [141]. This result illustrates how TAS can adjudicate between competing quenching mechanisms; PET/charge transfer versus FRET that produce near-identical steady-state PL quenching but arise from fundamentally different excitonic processes, a distinction of direct relevance to interpreting QD-pollutant interactions where the dominant mechanism is otherwise ambiguous. Despite its mechanistic power, TAS carries significant practical limitations that restrict its routine application in environmental QD sensing research. The technique requires an ultrafast laser system (typically a Ti:sapphire amplifier with optical parametric amplification), making it expensive, laboratory-bound [142], and inaccessible to most environmental sensing groups. The measurements are also highly sensitive to sample concentration, pump fluence, and photodegradation, requiring careful experimental control to avoid artefacts from multi-photon excitation or sample degradation during measurement. Furthermore, TAS does not directly report on the analytical sensing signal itself; it reveals the ultrafast photophysics that precede the steady-state emission used for sensing, providing mechanistic insight rather than quantitative analytical data [143]. Its primary evaluative contribution to the sensing literature is therefore adjudicatory rather than operational: it is most valuable when two competing mechanistic interpretations (e.g., FRET versus charge transfer) produce indistinguishable steady-state and TRPL signatures and a definitive mechanistic assignment is needed to guide rational sensor optimisation. For the majority of environmental QD sensing studies, TAS is not operationally necessary but represents the gold standard for mechanistic validation of charge-transfer-based transduction pathways.

### 6.4. Excitation–Emission Matrix (EEM) Fluorescence Spectroscopy

Excitation–emission matrix (EEM) fluorescence spectroscopy coupled with chemometric analysis has emerged as an important approach for environmental sensing applications involving complex samples. Unlike conventional emission scans, EEM provides multidimensional fluorescence fingerprints that capture variations arising from QD emission, analyte-induced emissive states, and background matrix components [62,102]. Multivariate methods, including parallel factor analysis (PARAFAC) and principal component analysis (PCA), allow separation of overlapping fluorescence components and enable pollutant monitoring even in the presence of unknown interferences [144]. This approach is particularly relevant for CQD-based sensors, where emission frequently originates from multiple surface states rather than a single defined transition [62,102]. EEM-PARAFAC approaches have therefore become useful for resolving polymer-specific fluorescence responses, microplastic interactions, and complex environmental signatures that cannot be distinguished using single-wavelength measurements. Critically, EEM-PARAFAC offers a methodological advantage that is directly relevant to the tripartite mechanism framework of this review: it can separate scattering contributions, inner filter effect artefacts, and genuine QD-analyte excitonic interactions into distinct PARAFAC components, enabling mechanistic attribution that is impossible from a single-wavelength emission scan. This is particularly important for microplastic sensing, where scattering by particles can produce apparent fluorescence changes that are indistinguishable from surface-state quenching in a conventional PL measurement. However, EEM-PARAFAC has its own limitations that must be acknowledged. The technique assumes a trilinear data structure, which is violated when the excitation-dependent emission of CQDs shifts with analyte concentration, a condition that can cause model misfit or artefactual component splitting. PARAFAC model validation requires careful selection of the number of components, ideally confirmed by split-half analysis and core consistency diagnostics, and this validation step is frequently omitted in the sensing literature. Additionally, EEM measurements are significantly slower than single-wavelength PL scans, making them less suited to rapid on-site detection applications. Despite these limitations, EEM-PARAFAC currently represents the most powerful available tool for mechanistic resolution of complex QD–environmental matrix interactions and for genuine real-sample validation of sensing specificity, particularly in systems with excitation-dependent emission such as CQDs.

### 6.5. Quantum Yield (QY) Measurements

Quantum yield (QY) determination provides a direct measure of the efficiency of radiative exciton recombination and is therefore a key parameter for evaluating excitonic sensing mechanisms. Since fluorescence intensity alone can be affected by absorbance changes, concentration variation, and inner filter effects, QY measurements provide a more mechanistically meaningful assessment of whether analyte interactions enhance emission by suppressing non-radiative pathways or decrease emission through exciton trapping. Within the Defect–Exciton Energy Map framework (Figure 5), QY serves as an experimental indicator of the balance between surface defects, trap density, and exciton accessibility, allowing comparison of different QD architectures beyond simple fluorescence intensity changes [100]. Despite its conceptual importance, QY measurement is frequently performed inconsistently across the QD sensing literature, limiting cross-study comparability. The relative method comparing integrated emission of the QD sample against a fluorescent standard of known QY under matched absorption conditions is susceptible to errors from spectral mismatch between QD and standard, refractive index differences between solvents, and imprecise absorbance matching. The absolute method using an integrating sphere avoids these systematic errors but requires specialised equipment and careful correction for scattered excitation light. Neither method accounts for the excitation-dependent QY that is characteristic of many CQD systems, where different excitation wavelengths access different surface states with different radiative efficiencies, a complication that is rarely addressed in the CQD sensing literature. For sensing applications, the most mechanistically informative QY measurement is not the absolute value, but the analyte-induced QY change relative to a well-characterised baseline: this quantity directly reports on the net change in the radiative-to-non-radiative rate ratio caused by the sensing event, providing a cleaner mechanistic signal than raw intensity changes that confound QY and absorbance effects.

### 6.6. Single-Particle Spectroscopy

Single-particle spectroscopy, including single-QD PL and photon-correlation measurements, provides access to excitonic phenomena that are averaged out in ensemble measurements [129]. These techniques enable direct observation of blinking behaviour, exciton localisation, Auger recombination, fine-structure splitting, and dark exciton populations. Biexciton blinking statistics measured at the single-particle level have been used to disentangle the Auger-mediated charge-trapping pathways that underlie ensemble-level signal fluctuations in core–shell QDs [47]. Although not commonly implemented in routine environmental sensing, single-particle studies provide fundamental insight into defect formation and exciton dynamics, helping explain ensemble-level phenomena such as fluorescence intermittency, quenching, and heterogeneous sensing responses. The critical evaluative point for single-particle spectroscopy in the sensing context is that ensemble-averaged measurements, the standard in environmental sensing, systematically conceal heterogeneity that can be analytically significant. A population of QDs with a bimodal distribution of surface trap densities will produce a single averaged TRPL lifetime that correctly describes neither sub-population and may lead to incorrect mechanistic conclusions about analyte-QD interactions. Single-particle measurements reveal this heterogeneity directly, but at significant cost: the technique requires confocal or near-field microscopy with single-photon counting detectors, is sensitive to oxygen and humidity (necessitating inert atmosphere sample environments), and produces data from individual particles that may not be representative of the ensemble used in sensing. For practical environmental sensing, single-particle spectroscopy is not operationally viable; its contribution is foundational, establishing the mechanistic picture of blinking, dark exciton populations, and trap dynamics that allows correct interpretation of ensemble sensing signals rather than being directly applicable to field analysis.

### 6.7. Electrochemiluminescence (ECL)

Electrochemiluminescence (ECL) (also called electrogenerated chemiluminescence) represents a distinct excitonic sensing pathway in which excited states are generated through electrochemical charge injection rather than optical excitation [145]. ECL measurements combine electrochemical control with optical detection to investigate electrically generated excitons and their interaction with analytes [130]. This technique has enabled highly sensitive sensing platforms by reducing optical background interference and improving signal-to-noise ratios. Recent QD-based ECL sensors have achieved exceptionally low detection limits through controlled electron–hole generation and efficient exciton emission pathways, demonstrating the analytical advantages of electrically driven excitonic transduction [100,146]. An aggregation-induced-emission ECL aptasensor based on red-emissive sulphur quantum dots achieved a linear range of 1.0 × 10^−13^ to 1.0 × 10^−8^ mol/L for the organophosphate pesticide malathion, with an LOD of 0.219 fM, performance attainable specifically because ECL eliminates the excitation-light background that limits fluorescence-based detection [108]. ECL biosensors built around QD as emitters have similarly been engineered for heavy-metal ion monitoring, where the target ion’s interaction with a biorecognition element directly modulates the ECL signal generated at the electrode [125,147], and perovskite QD-based ECL platforms (CH_3_NH_3_PbBr_3_ QDs@SiO_2_) have been used for ultrasensitive detection of the mycotoxin aflatoxin B1 in food matrices demonstrating that the ECL measurement format generalises across QD families and across both inorganic and organic pollutant classes [148]. The primary analytical advantage of ECL over fluorescence-based QD sensing, the absence of excitation light, simultaneously eliminates two of the most consequential sources of analytical error: the IFE from matrix components absorbing at the excitation wavelength, and autofluorescence from biological or environmental matrix components excited by the same light source. This makes ECL inherently better suited than fluorescence to complex environmental matrices such as wastewater effluents, sediment extracts, and coloured leachates where the IFE and autofluorescence are severe. However, ECL carries its own critical limitations. The technique requires an electrochemical cell and potentiostat in addition to an optical detector, constraining portability relative to fluorescence-based sensors. QD film deposition on the electrode surface must be reproducible, a challenge given the tendency of QDs to aggregate or redistribute under applied potential and ECL efficiency is sensitive to electrode surface fouling by matrix components, which can progressively attenuate signal in continuous monitoring applications [149]. Additionally, as discussed in Section 5.8, the excitonic character of the emitting state in coreactant-pathway ECL is not always equivalent to that of the photogenerated exciton, and mechanistic claims about ECL-based sensing should specify which pathway is operative rather than treating all ECL responses as equivalent excitonic transduction.

### 6.8. Complementary Structural and Surface Characterisation

Optical techniques alone cannot fully establish the origin of excitonic responses; therefore, structural and chemical characterisation methods, including FTIR, XPS, XRD, and TEM/HRTEM, are essential for linking observed sensing behaviour to specific material features. FTIR identifies functional groups involved in ligand binding and surface passivation, XPS reveals oxidation states, defect chemistry, and heteroatom configurations, while XRD and TEM confirm crystal structure, particle size, and confinement regime. The integration of structural characterisation with time-resolved optical measurements provides the mechanistic connection between synthesis-controlled features and excitonic sensing performance. As demonstrated in QD studies, combining microscopic analysis with spectroscopic techniques is essential for identifying whether sensing responses originate from defect engineering, surface modification, charge transfer, or exciton recombination dynamics [132]. Attenuated Total Reflection Fourier-transform infrared spectroscopy (ATR-FTIR) has been used to probe interfacial rigidification processes of polyamide-derived CQDs (PACQD) for polyamide microplastics sensing. This is done specifically by tracking shifts in the Amide I (C=O) stretching (1600–1700 cm^−1^) and Amide II (N-H) bending and (C-N) stretching (1470–1570 cm^−1^), a process that induces AIE, resulting in a strong fluorescence “turn-on” response that selectively tags the microplastics [62,102]. Together, these techniques establish a hierarchy of excitonic resolution: steady-state methods determine whether a sensing response occurs; time-resolved and ultrafast techniques reveal the responsible excitonic pathways; chemometric and structural approaches identify the molecular and material origins of the response. No single technique is sufficient for complete mechanistic characterisation, and the most credible sensing studies combine, at minimum, steady-state PL, TRPL lifetime measurement for mechanism discrimination, IFE correction using UV-Vis absorption data, and structural characterisation linking synthesis-controlled features to excitonic response. This integrated framework is essential for transforming QD sensing from empirical fluorescence optimisation into genuinely predictive exciton-engineered design.

## 7. Application to Major Pollutant Classes

The mechanistic framework established already finds its most direct expression in the sensing of specific environmental pollutants, where the chemical identity of the analyte dictates which excitonic transduction pathway dominates, which QD material has been demonstrated to perform effectively, and what design choices maximise analytical performance. This section applies the excitonic lens systematically to four major pollutant classes, including heavy metal ions, PFASs and persistent organic pollutants, microplastics and nanoplastics, and emerging contaminants, demonstrating that performance differences across the literature are not random but are mechanistically predictable from the principles developed in the previous section. Table 2 summarises study-level performance data for representative QD-based sensors across each pollutant class, including primary references, matrices, linear ranges, LOD values with calculation methods where reported, recovery data, and real-sample validation status. Direct cross-row comparisons should be made with caution given the heterogeneous assay conditions, matrices, recognition chemistries, and LOD calculation methods across studies.

### 7.1. Heavy Metal Ions

**Figure 9:**
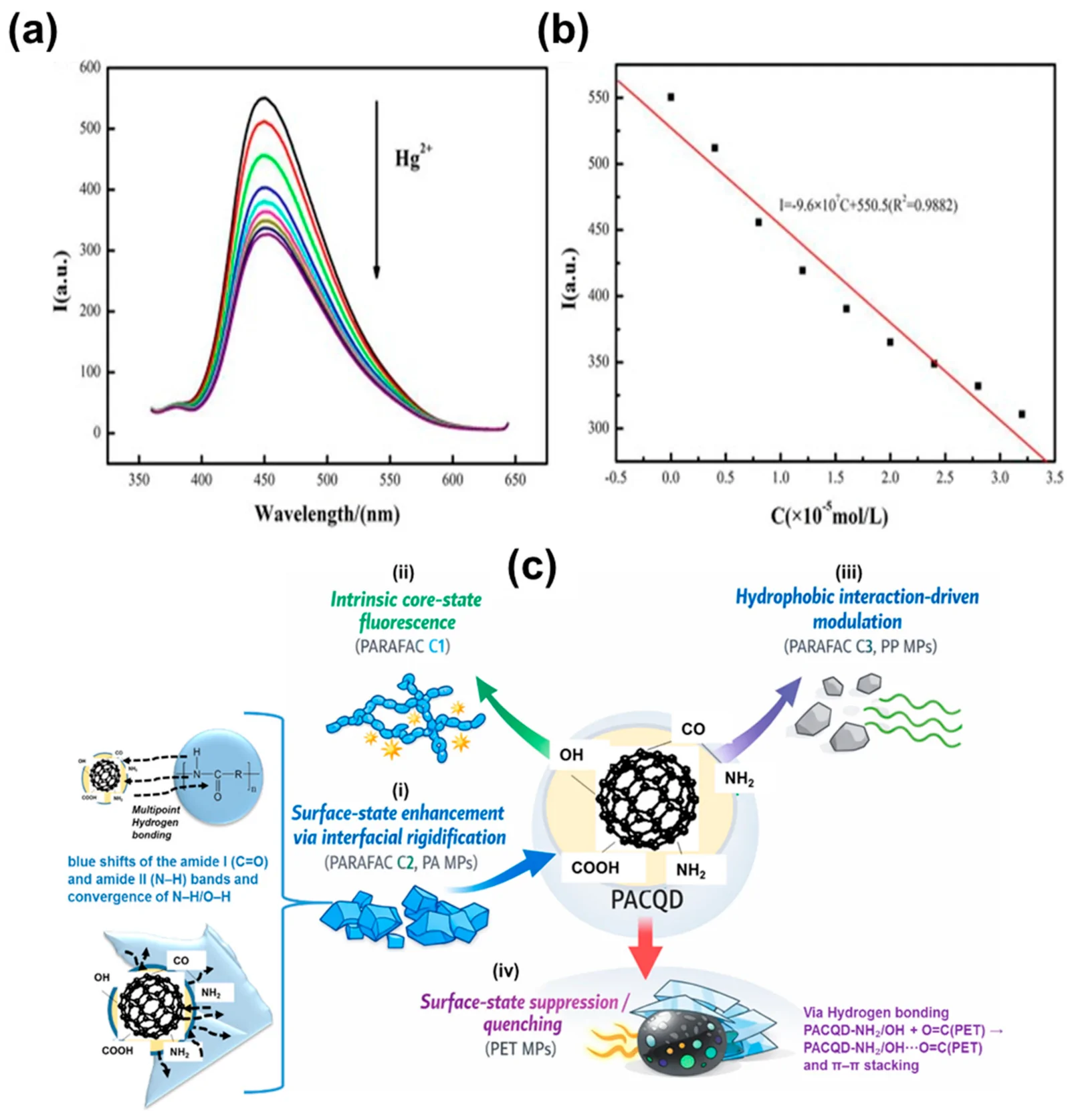
(**a**) Fluorescence response of CdSe QDs to increasing Hg^2+^ concentrations, illustrating PL quenching associated with exciton perturbation. (**b**) Calibration relationship between fluorescence intensity and Hg^2+^ concentration, demonstrating quantitative sensing capability (adapted from [131]). (**c**) Multi-pathway fluorescence transduction mechanisms at the PACQD–microplastic interface resolved via chemometric modelling. The schematic delineates microplastic-specific optical modulation channels: (**i**) surface-state amplification via hydrogen-bond-mediated interfacial rigidification when interacting with polyamide (PA) matrices; (**ii**) independent, invariant core-state fluorescence tracking (sp^2^ domains); (**iii**) localised dielectric modulation driven by hydrophobic interactions with polypropylene (PP); and (**iv**) severe non-radiative surface-state quenching induced by synergistic hydrogen bonding and π–π stacking with polyethylene terephthalate (PETMP) structures (adapted from [62]).

Heavy metal ions represent the most extensively studied pollutant class in QD-based sensing, and for good reason: they are globally distributed, bioaccumulative, non-biodegradable, and toxic at trace concentrations well below current analytical detection capabilities in resource-limited settings [93]. Mercury (Hg^2+^), lead (Pb^2+^), cadmium (Cd^2+^), copper (Cu^2+^), chromium (Cr^6+^), arsenic (As^3+^), and iron (Fe^3+^) collectively account for the majority of priority metal contaminants listed by environmental regulatory agencies worldwide. PQDs have demonstrated particular promise for ultrasensitive heavy metal ion detection, leveraging high PLQY, narrow emission spectra, tunable bandgaps, and defect-tolerant optoelectronic properties, with documented mechanisms, including cation exchange, electron/hole transfer, FRET, and surface trap-mediated quenching [96]. It should be noted, however, that direct performance comparisons between PQDs and other QD families are complicated by differences in assay format, matrix, recognition chemistry, and LOD calculation method, as detailed in Table 2. Mercury (Hg^2+^) is among the most effective fluorescence-quenching heavy metal ions for QDs across different material families, owing to its strong affinity for sulphur-containing surface ligands and its ability to undergo cation exchange reactions within QD lattices [152]. In chalcogenide QDs, such as CdSe and CdTe, Hg^2+^ can replace surface Cd^2+^ through a cation exchange process driven by the higher thermodynamic stability of HgS and HgTe compared with their cadmium counterparts [152,160]. This process strongly perturbs the excitonic structure by modifying the lattice composition and introducing electronic states that facilitate non-radiative recombination. Lee and Smith [152] demonstrated that Ag^+^ can act as a catalyst for cation interdiffusion, lowering the CdSe–HgSe alloying temperature from 250 °C to 80 °C. In conventional cation exchange (CE) and interdiffuser-enhanced cation exchange (IE-CE) processes, quasi-spherical CdSe nanocrystals (3.3 nm diameter, zinc blende structure) treated with Hg^2+^ or Hg^2+^/Ag^+^ exhibited an immediate colour change from orange-red to black, indicating rapid replacement of Cd^2+^ by Hg^2+^ within the nanocrystals. The reaction proceeded more extensively in the presence of Hg^2+^/Ag^+^, while Hg^2+^ treatment alone caused the lowest-energy 1S–1S absorption transition to redshift by 0.74 eV (from 2.24 to 1.50 eV) after 1 h [152]. These structural and electronic transformations generate deep mid-gap trap states, resulting in severe exciton quenching and enabling Hg^2+^ detection at nanomolar concentrations [131,152]. The quenching behaviour of Hg^2+^ has also been exploited in fluorescence resonance energy transfer (FRET)-based sensing platforms. Wang et al. [131] investigated a CdSe QDs/g-C_3_N_4_ nanosheet fluorescence sensor for Hg^2+^ detection and demonstrated that Hg^2+^ addition caused a progressive decrease in fluorescence intensity due to efficient quenching of the FRET system (Figure 9a). The sensor response increased with Hg^2+^ concentration, and the fluorescence intensity ratio (I/I_0_), where I_0_ and I represent the emission intensity in the absence and presence of Hg^2+^, respectively, showed a concentration-dependent relationship (Figure 9b). A ZnO/C dual-QD fluorescent sensor for Hg^2+^ demonstrated excellent linearity over 1–25 μmol/L with an LOD of 0.347 μmol/L, with spiked recovery experiments in real wastewater yielding 99.6–100.4% recovery and RSD below 5% [150]. These studies highlight Hg^2+^ as a representative analyte where exciton perturbation through lattice transformation, trap-state formation, and charge-transfer interactions provides a powerful pathway for pollutant sensing.

In perovskite QD systems, CH_3_NH_3_PbBr_3_ PQDs achieve detection of Hg^2+^ at limits as low as 0.124 nM through a combined mechanism of cation exchange and electron transfer [96]. For CQD-based sensors, fluorescent CQDs derived from diverse precursors demonstrate Stern–Volmer analysis revealing strong linear quenching for Hg^2+^ (Figure 9a,b), with the primary quenching mechanisms involving electrostatic interactions, π–π interactions, and energy transfer. It is important to note that inner filter effects (IFEs) have also been identified as contributors to the apparent quenching signal in some CQD–Hg^2+^ systems, and their contribution should be experimentally separated from genuine excitonic quenching through lifetime measurements and IFE correction before mechanistic conclusions are drawn [27]. Thiol-directed surface engineering yields sub-nanomolar LODs [97], as detailed in Table 2.

Lead (Pb^2+^) quenching of QDs operates predominantly through trap-state creation, with the Pb^2+^ ion coordinating to surface chalcogenide sites and introducing hole-trap states that intercept the photogenerated exciton before radiative recombination. Nitrogen/phosphorus co-doped CQDs (N/P-CQDs) have been applied to dual detection of Pb^2+^ and chlortetracycline, achieving recovery values of 92–98% in river water, tomato, and milk matrices [154], demonstrating real-sample applicability in both environmental and food safety contexts. Deep learning-optimised N-CQD biosensors achieve detection limits of 5–10 nM for heavy metals, including Pb^2+^, representing a 50–100-fold improvement over conventional CQD sensors through the combination of superior surface chemistry and CNN-LSTM attention-based spectral analysis [161]. The machine-learning enhancement extracts excitonic signal information from spectral features invisible to conventional threshold-based analysis.

Cadmium (Cd^2+^) presents a uniquely interesting sensing challenge because many of the most sensitive QD sensors are themselves cadmium-based, creating the paradox of using a cadmium-containing sensor to detect cadmium contamination. This has driven interest in cadmium-free sensing platforms, particularly CQDs and InP-based sensors. A ratiometric CQDs/CdTe QD sensor for Cd^2+^ achieved an LOD of 0.018 μM over a linear range of 0.1–23 μM and was successfully validated in real rice samples, with a smartphone-integrated readout for portable field application [155]. Carbon dots have been widely reported for the detection of Cd^2+^ in aqueous solutions with high sensitivity, exploiting the interaction between surface functional groups and Cd^2+^ coordination chemistry to produce selective quenching responses against panels of competing metal ions [162].

Chromium (Cr^6+^) occupies a distinct mechanistic niche among heavy metal ion targets because hexavalent chromium is itself a strong UV absorber with significant spectral overlap with many QD excitation and emission windows, making the IFE a primary rather than incidental contribution to quenching. As discussed in Section 5.1, this is a case where the IFE operates as the dominant signal-generating process rather than genuine excitonic perturbation, where the QD excitonic state is not directly modified by Cr^6+^ interaction, and TRPL lifetime measurements confirm an unchanged exciton lifetime upon Cr^6+^ addition. The IFE can nonetheless be exploited as an analytically useful mechanism when the analyte’s absorption is sufficiently distinctive and stable [103]. N-CQDs demonstrate notable selectivity and sensitivity for Fe^3+^, Cu^2+^, and Hg^2+^ ions, with the primary quenching mechanisms involving electrostatic interactions, π–π interactions, the IFE, and energy transfer in a manner that is ion-specific due to differential contributions of each mechanism per target ion [97].

Multiplexed heavy metal detection represents the next frontier beyond single-ion sensing and is where QD platforms demonstrate their most distinctive advantages over conventional analytical methods. A comprehensive analytical assessment of QD-based sensors drawing on 124 peer-reviewed articles finds the geometric mean LODs of 38 nM for QD-fluorescent-based sensors, 26 nM for QD-phosphorescent-based sensors, and an impressively low 0.109 pM for QD-chemiluminescent-based sensors, demonstrating by far the best sensitivity in QD-based detection [100]. These geometric means aggregate data across highly heterogeneous assay conditions and should be interpreted as indicative benchmarks rather than direct performance comparisons. AI-based sensing methods, including ATTBeadNet models, optimised principal component analysis, and SVM-based systems, further enhance the analytical performance of QD detection, with deep learning approaches achieving R^2^ = 0.97 ± 0.02 and RMSE below 4% across six contaminant classes simultaneously [161].

### 7.2. PFASs and Persistent Organic Pollutants

Per- and polyfluoroalkyl substances present a fundamentally different sensing challenge from heavy metal ions. Where metal ions are chemically reactive, coordinatively specific, and often electrochemically active, PFAS compounds are chemically inert, lack specific binding groups found in conventional recognition elements, and derive their environmental persistence from the extraordinary strength of the C–F bond. Their detection by QD-based sensors therefore cannot rely on the coordinative quenching or cation exchange mechanisms that are so effective for metal ions; instead, it demands surface engineering strategies that exploit the hydrophobic and electrostatic character of PFAS molecular structures, principally through surface charge-transfer perturbation and dielectric environment modulation [9,156]. The perfluoroalkyl chain, a sequence of C–F bonds with the highest known electronegativity per unit length creates a strongly electron-withdrawing surface environment when adsorbed onto a QD surface. This local electron withdrawal reduces the electron density at the QD surface anion sites, effectively increasing the local dielectric constant experienced by the confined exciton through the Coulomb screening term of the Brus equation (Section 2.2, Equation (1)). The consequence is a measurable reduction in emission and lifetime-based intensities [163]. Silicon quantum dot–molecularly imprinted polymer (MIP) hybrids integrate up-conversion fluorescent silicon QDs with molecularly imprinted polymers to achieve dual-mode PFOA detection, with the interaction between PFOA and MIPs resulting in fluorescence quenching with an LOD of 37.5 nmol/L for the low-background up-conversion fluorescence detection of PFOA and 73.9 nmol/L for the portable smartphone sensing platform successfully applied to the detection of PFOA in environmental waters and food samples [156]. In this system, the MIP provides the binding selectivity that the QD surface chemistry alone cannot achieve; the molecular imprint cavity is geometrically and chemically complementary to PFOA, excluding shorter-chain congeners and non-fluorinated carboxylates, while the QD provides the excitonic optical transduction. The sensing signal arises from dielectric modulation of the confined exciton by PFOA molecules occupying MIP cavities in proximity to the QD surface, rather than from direct PFOA-QD surface coordination. This architecture therefore operates through the excitonic landscape modulation mechanism mediated by the MIP rather than through direct surface quenching. Ratiometric detection of perfluoroalkyl carboxylic acids using dual fluorescent nanoparticles integrated with miniaturised microfluidic platforms provides self-calibrated concentration-dependent signals suitable for on-site analytical applications, directly addressing the fundamental selectivity challenge in PFAS sensing [157].

The selectivity challenge in PFAS sensing is severe: the structural similarity among PFAS congeners differing primarily in chain length and head group means that non-specific hydrophobic interactions with the QD surface cannot reliably distinguish individual compounds. This selectivity limitation arises from the intrinsically nonspecific nature of many QD–PFAS interactions. Dielectric modulation, electrostatic attraction, fluorophilic interactions, adsorption, and partial charge redistribution can produce differential excitonic responses without necessarily providing molecular-level recognition. As a result, structurally related PFAS, non-PFAS surfactants, dissolved organic matter, and other surface-active matrix components may generate overlapping responses, particularly in complex environmental matrices. Although QD surface functionalisation can improve differential selectivity through fluorophilic, electrostatic, or hydrophobic interactions, such intrinsic selectivity does not necessarily enable reliable discrimination among closely related PFAS congeners. Recent progress illustrates this transition from single-QD sensing toward more sophisticated recognition and pattern-based approaches. For example, a 2026 carbon-dot fluorescent array combining differently functionalised carbon dots with membrane enrichment and multivariate analysis successfully discriminated PFOA, PFOS, and PPVE in tap, river, and lake water, demonstrating that differential excitonic response patterns can provide congener-level discrimination when coupled with appropriate signal processing and sample enrichment [164]. Nevertheless, achieving robust molecular specificity generally benefits from an additional recognition or enrichment strategy, such as a molecularly imprinted polymer (MIP), aptamer, selective ligand, fluorinated interface, or membrane-based preconcentration, positioned upstream of or integrated with the excitonic transduction element. MIP coatings can provide target-specific binding sites, aptamer-functionalised QDs can exploit molecular recognition, and fluorinated surface ligands can enhance preferential interactions with PFASs through fluorophilic and hydrophobic effects. Importantly, each strategy alters the accessibility and electronic environment of the QD surface and may therefore influence not only selectivity but also the dominant excitonic transduction mechanism. Consequently, recognition-layer design should be evaluated together with fluorescence quenching or enhancement pathways, rather than treated as an independent component of sensor performance.

Pesticides constitute the most extensively studied sub-class of persistent organic pollutants for QD-based sensing. CQD@MIP composite materials integrate the specificity and stability of MIPs with the sensitivity and signal amplification capabilities of CQDs, making them ideal materials for the specific detection of pesticide residues with CQDs@MIPs significantly improving the insufficient selectivity of CQDs and solving the problem of identifying target molecules in complex environments through PET, FRET, and IFE mechanisms [158]. It is important to note that of these three co-operating mechanisms, only PET and FRET constitute genuine excitonic transduction; the IFE operates through optical absorption without excitonic perturbation, as established in Section 5.1.4. For organophosphate pesticides, fluorescence quenching can arise from photoinduced electron transfer (PET), in which a photoexcited electron from an emissive CQD state is transferred to an electron-accepting state of the pesticide, thereby introducing a non-radiative relaxation pathway and suppressing CQD fluorescence. Thermodynamic feasibility can be evaluated from the relative energies of the donor and acceptor states; defining the PET driving-force proxy as ΔE_PET_ = E_acceptor_ − E_donor_, a negative value indicates a downhill electron-transfer process. For electron-deficient organophosphates such as chlorpyrifos and methyl parathion, their low-lying acceptor states can facilitate PET from appropriately positioned CQD excited states. However, the magnitude of the driving force is strongly dependent on the CQD surface chemistry, emissive state, pesticide electronic structure, solvent environment, and computational/experimental energy-level reference. PET should therefore be considered a plausible quenching pathway rather than a universal mechanism for all CQD–organophosphate systems, because static/dynamic quenching, Förster resonance energy transfer, and inner-filter effects may also contribute [165,166]. Excited state intramolecular charge transfer (ICT) is particularly sensitive to changes in polarity and analyte interactions, making it effective for detecting polar molecules such as nitroaromatic compounds or specific toxins [165].

### 7.3. Microplastics and Nanoplastics (M/NPs)

Microplastic and nanoplastic sensing represents the newest and most mechanistically complex application domain for QD-based environmental sensors. Unlike dissolved chemical analytes, microplastics are heterogeneous particulate entities spanning size ranges from nanometres to millimetres, composed of multiple polymer chemistries, and present in environmental matrices at concentrations and size distributions that vary by orders of magnitude. These characteristics create optical sensing challenges with no direct analogue in ion or molecular detection: particle scattering, size-dependent optical effects, and the physical heterogeneity of MP populations all introduce non-excitonic contributions to optical signals that must be carefully separated from true QD-MP excitonic interactions.

Two principal detection modalities have emerged for QD-based MP sensing. The first, staining or labelling, involves adsorption of QDs onto MP surfaces, producing fluorescent MP-QD composites detectable by fluorescence microscopy or spectroscopy. Zhao et al. [167] reported N,Cl co-doped lignin-derived CQDs capable of detecting polystyrene MPs with a detection limit of 0.4 mg L^−1^, attributed to surface functional group interactions. Paramparambath et al. [168] achieved ultralow detection limits (0.0005 mg L^−1^) for polyethylene and polystyrene MPs using citric acid/urea-derived CQDs, highlighting the sensitivity of CQDs-based systems under optimised conditions. Jini et al. [169] developed CQDs from *Araucaria araucana* biomass for the detection of ≥6 μm MPs, demonstrating the influence of particle size on fluorescence response. Priyanto et al. [170] developed CQDs from citric acid/urea for detecting PETMPs in aqueous solutions. The second, solution-phase EEM fluorescence is mechanistically more complex but analytically more powerful. Ultra-fine polyamide-derived (PACQDs: ~1.4 nm) reveal polymer-specific photophysical responses in EEM space: PA and PP microplastics induce fluorescence enhancement of ~11.66% and ~11.43%, respectively, while polyethylene terephthalate microplastics (PETMP) cause net quenching (−4.61%) alongside a distinct red-shifted emission band; PARAFAC resolves the EEM data into three fluorescent components representing the intrinsic CQD core and two interaction-induced surface states, with PCA yielding clear separation of polymer classes where the first two principal components capture ~88% of total spectral variance, with ATR-FTIR providing molecular evidence, including amide-amide coupling and interfacial rigidification, induced AIE for PA MPs, hydrophobic interaction for PP MPs, and synergistic hydrogen bonding and π–π stacking for PET MPs (Figure 9c; [62]). Critically, these polymer-specific fluorescence fingerprints were validated in tap water, where the spectroscopic signatures were preserved despite elevated background intensity and partial contrast attenuation, providing genuine real-sample evidence of platform robustness [62,102]. This result demonstrates that excitonic transduction can encode polymer identity rather than merely particle presence, a capability with transformative implications for environmental MP monitoring where polymer-specific data is required for source apportionment and risk assessment. An important methodological note for all MP sensing studies discussed above: scattering contributions from MP particles must be explicitly separated from genuine excitonic quenching signals, since both produce apparent reductions in QD fluorescence intensity. Studies that do not report scattering correction or PARAFAC-based component separation should be interpreted with caution when mechanistic conclusions about excitonic quenching are drawn (Section 5.1; [62]). For nanoplastics, fragments below 1 μm that are analytically inaccessible to conventional optical microscopy, QD-based sensing offers particular promise because the nanoscale dimensions of the analyte are comparable to those of the QD probe, enabling molecular-scale interactions not possible with larger probes. CQDs synthesised from green precursors demonstrate high sensitivity in detecting emerging pollutants, including nanoplastics, with their excellent biocompatibility, low toxicity, and tunable surface chemistry supporting diverse sensing architectures across environmental and food safety matrices [89].

### 7.4. Emerging Contaminants: Nitroaromatics, Pesticides, Pharmaceuticals, and ROS

The category of “emerging contaminants” encompasses pollutants that have been identified in environmental matrices at concentrations of concern but for which regulatory frameworks, monitoring protocols, and analytical methods are still being developed. QD-based excitonic sensing is particularly well positioned for this category because the rapidity with which new contaminants are identified demands flexible sensing platforms that can be adapted to new targets without complete redesign.

Nitroaromatic compounds, including 2,4,6-trinitrotoluene (TNT), nitrobenzene, and nitrophenols, are potent PET acceptors whose electron-deficient aromatic cores accept photoexcited electrons from QD conduction bands with high efficiency. ICT is particularly sensitive to changes in polarity and analyte interactions, making it effective for detecting polar molecules such as nitroaromatic compounds, while a CQD@MIP composite sensor for the nitrofen herbicide demonstrates that the easily adjustable structures of CQDs allow enhancement of fluorescence properties through surface modification, with PET, FRET, and IFE operating synergistically for selective pesticide detection at ng/mL LODs [158]. CQDs have been employed to fabricate portable devices for the fluorometric detection of nitrosamine impurities in drug matrices, demonstrating the translational potential of QD excitonic sensing for pharmaceutical quality control and regulatory compliance monitoring [171].

Pharmaceutical residues, including antibiotics and emerging contaminants such as tetracycline, fluoxetine, ciprofloxacin, and chloramphenicol, interact with QDs through multiple concurrent pathways, with the dominant mechanism determined by QD surface chemistry, analyte structure, and solution conditions. Among these pathways, intramolecular charge transfer (ICT) plays an important role, where electron-donating and electron-accepting groups within the sensing system undergo excitation-induced charge redistribution, modifying the local electronic environment and fluorescence response. This mechanism is particularly effective for detecting polar pharmaceutical molecules in complex environmental and food matrices [165]. For example, Enyoh et al. [91] developed fluorescent CQDs derived from polyethylene terephthalate waste (PET-FCQDs) as sustainable sensing probes for ciprofloxacin (CIP) and fluoxetine (FLX) detection in aqueous systems through fluorescence enhancement. Stern–Volmer analysis confirmed strong analyte–QD interactions, with static quenching interactions identified for both CIP and FLX. The PET-FCQD sensor achieved linear detection ranges of 50–150 µg/L for CIP and 100–400 ng/L for FLX, with corresponding limits of detection (LOD) of 3.3 µg/L and 134 ng/L, respectively. Beyond conventional sensing approaches, deep learning-optimised N-CQD biosensors have further improved pharmaceutical detection performance, achieving detection limits of 20–80 nM and representing a 50–100-fold enhancement compared with traditional CQD-based sensors [161]. These advances demonstrate how rational control of QD surface chemistry and excitonic interactions can improve the sensitivity and selectivity of pharmaceutical pollutant detection.

Reactive oxygen species (ROS), including peroxynitrite (ONOO^−^), hydrogen peroxide (H_2_O_2_), superoxide (O_2_^−^), and hydroxyl radical (•OH) are oxidative stress markers that indicate microbial activity, industrial discharge, and environmental redox stress, and whose selective detection represents one of the most mechanistically demanding challenges in QD-based environmental sensing. Green CQDs are naturally enriched with heteroatoms that enhance their functional properties, including scavenging of reactive oxygen species, thereby demonstrating dual sensing and remediation functionality relevant to environmental monitoring of oxidative stress conditions [89]. However, the primary analytical challenge in ROS sensing is not sensitivity but selectivity: ROS species are chemically reactive, short-lived, and structurally similar, meaning that direct QD surface quenching, which lacks the chemical specificity to distinguish ONOO^−^ from H_2_O_2_ or •OH, is insufficient as a standalone sensing strategy. The most powerful approach for achieving ROS selectivity in QD-based platforms is to decouple analyte recognition from optical transduction through a receptor-FRET architecture, where a molecularly specific receptor is assembled onto the QD donor surface, and the QD provides only the reference donor emission channel. This principle is exemplified by the CD–N-I nanoprobe reported by [104], in which a naphthalimide–isatin receptor chosen for its selective chemical reactivity toward ONOO^−^ is assembled onto carbon dots through electrostatic interactions. Prior to ONOO^−^ addition, the isatin blocking group prevents intramolecular charge transfer (ICT) within the naphthalimide acceptor, keeping the FRET channel optically inactive and the system in a TICT-suppressed state. ONOO^−^ selectively cleaves the isatin receptor, unmasking the naphthalimide acceptor and switching on FRET from the CD donor, converting a non-emissive TICT state into an emissive locally excited (LE) state with a concurrent ratiometric change in donor-to-acceptor emission ratio. The resulting probe detects ONOO^−^ with high selectivity over fourteen competing ROS and reactive nitrogen species, achieving an LOD of 0.22 μM, performance unachievable through direct surface quenching alone [104]. This example establishes three design principles directly generalisable to environmental ROS monitoring. First, analyte selectivity should be encoded in the molecular receptor rather than the QD surface chemistry, using chemically well-defined reactions specific to the target ROS. Second, ratiometric FRET architectures provide self-calibrated signals immune to probe concentration and excitation intensity variations that would confound single-channel measurements in turbid or coloured environmental matrices. Third, the correct assignment of the operative excited-state mechanism-distinguishing excited-state intramolecular proton transfer (ESIPT), twisted intramolecular charge transfer (TICT), intramolecular charge transfer (ICT), direct quenching, and FRET is essential for rational probe optimisation: each produces a distinct spectroscopic signature (a large Stokes-shifted emission for ESIPT, a red-shifted charge-transfer band for TICT, a concentration-dependent spectral shift for ICT, a lifetime change for dynamic quenching, and sensitised acceptor emission for FRET) and misattribution to the IFE or nonspecific quenching leads to erroneous mechanistic conclusions and suboptimal sensor design [104,113].

## 8. Comparative Analysis: Excitonic QD Sensors vs. Other Sensing Strategies

This section provides critical benchmarking, organised not as an advocacy exercise for QD sensors but as a mechanistically informed comparison whose goal is to identify where excitonic transduction offers genuine, non-incremental advantages, where it faces fundamental limitations, and what the most productive paths for integration with competing platforms are. Honest assessment of limitations is, in the long run, more useful to the field than uncritical celebration of performance metrics. It should be noted at the outset that the performance ranges in Table 3 aggregate data from studies with heterogeneous analytes, matrices, assay formats, and LOD calculation methods; the comparisons are therefore indicative rather than definitive, and direct cross-platform conclusions should be drawn with caution.

### 8.1. Benchmarking Against Established Sensing Platforms

QD-based sensors compete with several established environmental sensing platforms, including organic fluorescent dyes, upconversion nanoparticles (UCNPs), plasmonic nanoparticles, aptamer-based biosensors, molecularly imprinted polymers (MIPs), and enzyme-based sensors. Their relative analytical performance in any specific application is determined by the combination of transduction mechanism, recognition element, matrix conditions, and assay format and no single platform is universally superior across all analyte-matrix combinations.

Organic fluorescent dyes represent the traditional benchmark for optical sensing. Compared with dyes, QDs offer narrow and tunable emission, broad excitation windows, generally high quantum yields, improved photostability, and reduced blinking, enabling multiplexed detection and long-term monitoring [179]. Under optimised laboratory conditions, QD-based systems have been reported to achieve detection limits as low as 10^−15^ M, compared with typical nanomolar (~10^−9^ M) sensitivity reported for organic dyes, a difference attributed primarily to superior excitonic brightness and stability [180]. However, this comparison should be interpreted with caution: the femtomolar LODs are typically achieved by ECL or chemiluminescence-mode QD sensors in controlled conditions, not by conventional fluorescence-mode QD sensors, and the gap narrows substantially in complex environmental matrices where the IFE, scattering, and non-specific adsorption attenuate QD performance [100]. Furthermore, the longer and tunable lifetimes of QDs (10–100 ns for chalcogenide QDs and up to microseconds for phosphorescent CQDs) enable time-gated detection, reducing interference from short-lived autofluorescence backgrounds [100,179]. UCNPs provide a unique advantage through near-infrared (NIR)-excited anti-Stokes emission, eliminating autofluorescence and enabling deep optical penetration. They exhibit high chemical stability, long lifetimes, and low photodamage compared with conventional fluorescent probes [174]. However, their low quantum yields (typically <1% under low excitation power) limit sensitivity, while the requirement for high-power NIR excitation complicates portable deployment [181]. Moreover, UCNP sensing predominantly relies on luminescence resonance energy transfer (LRET) as its primary transduction mechanism, whereas QDs support multiple distinct excitonic transduction pathways as described in Section 5, providing greater mechanistic flexibility for diverse analyte classes. Plasmonic nanoparticles, particularly AuNPs and AgNPs, detect analytes through localised surface plasmon resonance (LSPR), aggregation-induced colour changes, and surface-enhanced techniques such as SERS, SEF, and SEIRA [182]. Their label-free operation and ability to provide molecular vibrational fingerprints are major advantages [183]. However, quantitative SERS remains limited by hotspot variability, and plasmonic LSPR responses often show weaker concentration dependence than fluorescence-based QD sensing in the sub-nanomolar range. Hybrid QD–plasmonic systems overcome some limitations by exploiting metal-enhanced fluorescence, where plasmonic structures amplify QD excitation and emission [180]. It should be noted that in hybrid QD–plasmonic systems, the analytical performance improvement arises from the combination of excitonic and plasmonic transduction rather than from either component alone; attributing sensing performance to either mechanism in isolation would be misleading.

Aptamer-based biosensors use nucleic acid recognition elements selected by SELEX to achieve high affinity and selectivity toward target molecules [184]. Their advantages include chemical stability, low cost, regeneration capability, and compatibility with synthetic modification compared with antibodies [176]. The integration of aptamers with QDs represents a particularly powerful strategy, combining molecular recognition specificity with excitonic signal amplification to improve environmental sensing performance [145]. The selectivity in aptamer-QD hybrids is encoded in the aptamer recognition element rather than in the QD excitonic properties; the QD functions as an optical reporter rather than as a selective transducer, and this distinction matters for mechanistic interpretation of reported LODs.

Molecularly imprinted polymers (MIPs) provide synthetic recognition sites generated through template-directed polymerisation and offer antibody-like selectivity with improved robustness [177]. However, their sensitivity is often limited without an efficient signal transduction mechanism. QD–MIP hybrids address this limitation by coupling selective analyte binding with excitonic optical responses; for example, Si-QD/MIP systems achieved dual-mode PFOA detection with limits of detection of 37.5 and 73.9 nmol/L, with real-sample validation in environmental waters and food samples [156], demonstrating performance beyond either component alone. Enzyme-based sensors exploit biological catalytic specificity and provide excellent selectivity for enzyme-related targets. However, their practical application is limited by enzyme instability under variable temperature, pH, heavy metal exposure, and storage conditions encountered in environmental monitoring [178]. QD–enzyme hybrids improve enzyme stability while retaining excitonic signal generation, although their application remains restricted to analytes associated with specific enzymatic reactions. Additionally, enzyme inhibition by non-target matrix components, including co-occurring heavy metals, can produce false-positive responses that are difficult to distinguish from target analyte effects without additional confirmatory measurements.

The comparative analysis shows that no single sensing platform is universally superior: each offers a distinct combination of strengths and limitations determined by its underlying transduction mechanism. QDs are distinguished by mechanistic versatility and spectral tunability, but their analytical performance advantage over competing platforms is analyte- and matrix-specific and is most pronounced in multiplexed detection, ratiometric self-calibrated measurements, and applications where photostability over extended measurement periods is required. Table 3 consolidates this comparative assessment across the key analytical and operational performance metrics most relevant to environmental deployment.

### 8.2. Unique Advantages of Excitonic QD Sensors

The comparative analysis above highlights that the advantages of excitonic QD sensors are not simply incremental improvements over existing platforms but arise from fundamentally distinct physical capabilities. These advantages originate from the unique tunability of QD electronic structures, excitonic behaviour, and surface chemistry, enabling sensing strategies that are difficult to achieve using conventional platforms.

*Mechanistic versatility within a single material class.* Unlike most sensing platforms that rely on a limited number of signal-generation pathways, QDs support a broad range of excitonic transduction mechanisms, including static and dynamic PL quenching, photoinduced electron transfer (PET), Förster resonance energy transfer (FRET), ratiometric dual-emission sensing, lifetime-based detection, charge-transfer excitons, and electrochemiluminescence (ECL). These responses can be engineered through surface modification, doping, and structural design while maintaining the same QD core. Furthermore, hybridisation of QDs with other nanomaterials provides additional signal amplification by combining QD optical properties such as tunable emission, strong absorption, and photostability with complementary functionalities of other materials [185].

*Simultaneous multiplexed detection.* The broad excitation windows and narrow, tunable emission profiles of QDs enable multiplex sensing, allowing multiple analytes or weak signals to be detected simultaneously with minimal spectral overlap [179]. This capability is particularly valuable for environmental monitoring, where complex samples may contain mixtures of pollutants such as heavy metals, pesticides, and pharmaceuticals. Compared with conventional fluorescent sensors, which often require additional integration with nanomaterials or microfluidic systems to achieve multiplexing and portability, QDs inherently provide spectral flexibility for advanced sensing architectures [173].

*Defect-engineered tunability from UV to NIR.* A defining feature of excitonic QDs is the ability to rationally tune their optical response through defect engineering, including heteroatom configuration, vacancy control, and surface functionalisation. This enables adjustment of emission and absorption behaviour across the UV–visible–NIR range, allowing sensor design to be matched to specific analyte properties and sample environments. Such continuous excitonic tuning is difficult to achieve in organic dyes or UCNPs, where optical transitions are largely determined by fixed molecular structures or crystal-field effects [24].

*Ratiometric and lifetime-based self-calibrated sensing.* QDs also provide intrinsic opportunities for self-referenced detection through dual-emission systems, dopant-induced emission, and long-lived excitonic states. These ratiometric and time-resolved approaches reduce errors associated with excitation fluctuations, probe concentration, and matrix effects, which are critical limitations in quantitative environmental analysis. The exceptionally low geometric mean LOD reported for QD chemiluminescence-based sensors (0.109 pM), compared with QD fluorescence (38 nM) and phosphorescence sensors (26 nM), demonstrates the performance diversity achievable by exploiting different excitonic readout mechanisms within the same material family [100].

*Sustainable sensing potential through CQDs.* Among fluorescent nanomaterials, CQDs offer a unique combination of high optical performance, environmental sustainability, biocompatibility, and low toxicity. While inorganic QDs provide excellent photostability and broad analytical ranges from pg/mL to mg/mL, their limited biodegradability and potential toxicity remain challenges. CQDs address these limitations by combining the functional advantages of inorganic QDs with the sustainability and compatibility characteristics of carbon-based materials [173].

Overall, the key advantage of excitonic QD sensors lies in their ability to transform molecular interactions into diverse and tunable optical responses. This combination of mechanistic flexibility, spectral programmability, self-calibration capability, and sustainable material design positions QDs as one of the most adaptable platforms for next-generation environmental sensing.

### 8.3. Key Limitations and Mitigation Strategies

Fluorescence blinking. Single-particle blinking, the stochastic switching between bright and dark excitonic states driven by Auger-mediated charge trapping, introduces signal instability that complicates quantitative sensing at low QD concentrations [47]. For ensemble sensing, where signals are averaged over millions of QDs, blinking is effectively self-averaged and does not materially compromise analytical performance. Mitigation strategies include thick-shell CdSe/CdS “giant QD” architectures that suppress Auger rates by spatially separating the exciton from the surface; InP/ZnSe core–shell systems that exhibit near-blinking-free behaviour attributed to an Auger blockade mechanism; and CQD systems, which generally exhibit reduced blinking relative to chalcogenide and perovskite QDs due to the heterogeneous distribution of emissive surface states and the absence of a sharp band edge supporting Auger-mediated charge ejection [156]. However, CQDs should not be described as inherently non-blinking: single-particle studies have documented intensity fluctuations in CQDs that, while mechanistically distinct from the on/off switching observed in CdSe QDs, nonetheless introduce signal variability at low concentrations and in single-particle sensing regimes [186]. For the ensemble-level environmental sensing applications that constitute the primary focus of this review, these residual fluctuations are effectively averaged out and do not compromise analytical performance.

Toxicity of heavy metal-based QDs. Lead-based PQDs achieve LODs as low as 0.1 nM with rapid response times below 10 s, while lead-free variants such as Cs_3_Bi_2_X_9_ and CsSnX_3_ offer eco-friendly alternatives with enhanced aqueous stability, demonstrating that regulatory pressure toward Cd/Pb-free alternatives is actively driving materials development that is beginning to approach the performance of their toxic predecessors [96]. The mitigation pathway is advancing on multiple fronts: CQDs, InP/ZnS QDs, Mn-doped ZnS QDs, and lead-free perovskite variants collectively provide non-toxic alternatives with analytical performance increasingly competitive with heavy metal-based systems. It is worth noting, however, that performance comparisons between toxic and non-toxic QD platforms must account not only for LOD but also for long-term photostability, aqueous stability, and matrix robustness, parameters on which non-toxic alternatives do not yet uniformly match their heavy metal counterparts [75].

Non-specific binding and surface fouling. In real environmental matrices, QD surfaces are exposed to humic acids, natural organic matter, proteins, competing ions, and colloidal particles that can adsorb non-specifically and produce analyte-independent changes in PL intensity, lifetime, and spectral position. It is important to distinguish two categories of matrix-induced signal change here: genuine excitonic perturbation caused by surface-adsorbed matrix components that create or passivate trap states, and non-excitonic optical artefacts such as inner filter effects arising from matrix absorbance or scattering by colloidal particles. Both categories reduce analytical accuracy, but they require different corrective approaches—surface engineering addresses the former, while optical correction methods and ratiometric or lifetime-based readout modes address the latter. Matrix interference in real samples can compromise detection accuracy, requiring sample pretreatment to eliminate interferents such as fats, metal ions, and natural organic matter before QD-based sensing can achieve reliable quantification [165]. Mitigation strategies include zwitterionic surface coatings that resist non-specific adsorption, encapsulation in silica or polymer shells that physically exclude matrix components while permitting target analyte diffusion, and the use of ratiometric or lifetime-based readout modes whose self-calibrating character inherently compensates for both non-specific excitonic perturbations and optical background contributions.

### 8.4. The Real-Sample Validation Gap

Perhaps the most consequential limitation of QD-based excitonic sensors and the one most systematically underreported in the primary literature is the gap between analytically impressive laboratory LODs and the practical performance achievable in real environmental matrices. A comprehensive analytical assessment of QD-based sensors drawing on 124 peer-reviewed articles reveals that while the lowest median LODs are calculated for environmental and industrial pollutants, no significant improvement has been observed in protein detection LOD values from 2015 to 2025, indicating that performance gains in controlled conditions are not translating into advances in matrix-robust detection [100]. This stagnation in the presence of continued synthetic innovation is a diagnostic signal: materials improvements are necessary but not sufficient, and the bottleneck lies in the interface between the QD platform and the real sample. The current literature is characterised by inconsistent LOD calculation methods, variable definitions of selectivity, different operational definitions of “real sample” validation, and the near-universal practice of reporting performance under optimised laboratory conditions without quantifying degradation in field-relevant conditions. CdTe/ZnS core–shell QD sensors demonstrate that when properly validated in real biological samples, achieving recovery rates of 86.4–98.0% with RSDs under 8.13%, the platform performs reliably; but such comprehensive real-sample validation remains the exception rather than the rule in the sensing literature [172]. The adoption of IUPAC-consistent LOD and LOQ calculation methods, mandatory authentic-matrix testing, standardised method validation protocols, and interlaboratory comparison studies would transform the credibility of QD-based environmental sensing in the eyes of regulatory agencies, the audiences whose adoption is ultimately required for the field’s societal impact to be realised.

## 9. Computational Approaches to Excitonic Sensing Design

This section addresses the increasingly powerful computational dimension of the field, the use of first-principles quantum chemical methods, molecular simulation, and machine learning to understand, predict, and rationally design excitonic properties for targeted sensing applications. Computation occupies a unique strategic position in the sensing design workflow: it can screen candidate materials and configurations at a fraction of the cost of experimental synthesis, provide mechanistic interpretations of experimental observations that are inaccessible to measurement alone, and ultimately enable inverse design, the specification of desired sensing properties and the computational identification of material configurations that achieve them. As the theoretical and algorithmic tools available to the field mature, the boundary between computational prediction and experimental validation is becoming increasingly productive, and the most impactful sensing advances of the next decade are likely to emerge from tightly integrated computational-experimental programmes. The summary of computational approaches to excitonic sensing design, including methods, applications, and benefits, is presented in Table 4.

### 9.1. Density Functional Theory and the Electronic Structure of Defect-Engineered QDs

Density functional theory (DFT) has become the workhorse of first-principles QD electronic structure calculation, providing ground-state geometries, electronic density of states, HOMO-LUMO gaps, Hirshfeld charge distributions, and defect state energy positions at a computational cost accessible with modern high-performance computing resources. The use of DFT and time-dependent DFT to study the properties of fluorescent biosensors, including QD-based systems, has grown substantially, with photophysical behaviour most commonly interpreted through molecular orbital diagrams based on Kohn–Sham orbitals, while a more accurate approach is to calculate excited states explicitly through TDDFT or higher-level wavefunction-based calculations that provide greater insights into the sensing mechanisms of the fluorescent probes [187].

For CQDs, systematic DFT investigations of vacancy defects, Stone–Wales defects, and heteroatom doping at varying concentrations in graphene quantum dot models reveal that defect type and concentration quantitatively govern absorption spectra and molecular orbital energy levels, providing actionable guidance for regulating optical properties through experimental control of defect identity and density [188]. The choice of functional and basis set is critical for predictive accuracy: generalised gradient approximation (GGA) functionals such as PBE significantly underestimate bandgaps through self-interaction error, making them unreliable for quantitative prediction of optical transition energies relevant to sensing. Hybrid functionals incorporating exact exchange, HSE06, B3LYP, PBE0, provide substantially improved bandgap accuracy, and DFT-guided sensing design for PQDs using hybrid functionals (HSE06 + SOC) with spin–orbit coupling systematically maps bandgap tunability from 1.8 to 3.0 eV through halide alloying in CsPbX_3_ and related systems, while adsorption-energy modelling reveals gas-surface interactions for ultrasensitive detection with sub-0.1 eV precision [67].

A particularly consequential application of DFT to excitonic sensing design is the computational investigation of functionalised graphene QD (GQD)-heavy metal interactions. DFT simulations of GQDs functionalised with thiol (-SH) and amino (-NH_2_) groups for the selective detection of heavy metals in wastewater reveal that thiol-functionalised GQDs exhibit high affinity for Pb and Cd, with an energy gap of 0.02175 eV in the interaction with Pb, showing optimised reactivity, while amino-modified GQDs present higher energy gaps, indicating lower reactivity and efficacy in contaminant identification [189]. This DFT-guided functional group selection, predicting which surface modification will produce the strongest response to a given heavy metal, bypasses the empirical screening of QD-analyte combinations that currently consumes large experimental resources and directly implements the Defect–Exciton Energy Map design principles established in Section 4.5. The studies discussed above demonstrate that it is possible to use DFT and TDDFT calculations to characterise the properties of QD-based sensors and rationalise the photophysical processes underlying the sensing mechanism, with the ability to screen candidates computationally before synthesis representing a fundamental shift in the efficiency of sensor development cycles [187].

For defect-cascaded photodegradation directly relevant to the long-term stability of QD sensors in environmental deployment, DFT calculations of sulphur vacancy pairs in InP/ZnSe/ZnS QDs reveal the atomistic mechanism of photodegradation, demonstrating how type-II band alignments at the InP/ZnSe interface facilitate defect-cascade formation under illumination and providing design guidance for suppressing this degradation mode through interfacial engineering [190]. This computational prediction of degradation mechanisms not merely optical properties illustrates the expanding scope of DFT from property prediction to stability design, with direct consequences for the environmental deployment lifetime of QD-based sensors.

### 9.2. Time-Dependent DFT for Optical Properties and Excitonic Characterisation

While ground-state DFT characterises the electronic structure, the prediction of optical properties; absorption spectra, emission wavelengths, oscillator strengths, and excitonic character requires time-dependent DFT (TD-DFT), which extends the Kohn–Sham formalism to the time domain and provides access to excited-state properties at computational costs accessible for QD-sized systems. The versatility and efficiency of TDDFT have made it a particularly attractive approach for studying the unique characteristics of GQDs, with numerous studies utilising TDDFT to investigate optical properties, including the impact of nitrogen dopant configuration, the utilisation of enhanced light absorption, and the influence of edge modifications [191].

For CQDs, TD-DFT analysis of N-doped graphene oxide QDs reveals that only specific functional group configurations, particularly carbonyl (=O) edge modification convert the lowest excited state S_1_ to a charge-transfer character; both edge =O/–OH and doped N can make a red-shift for the absorption spectra, while edge modification and nitrogen doping alone are insufficient to generate strong S_1_ transition intensity [192]. This finding directly guides experimental functionalisation strategies: only carbonyl-edge modified CQDs will produce the charge-transfer excitonic response needed for PET-based sensing of electron-accepting analytes, while amine-functionalised or nitrogen-doped systems without carbonyl groups will not, a prediction that would require extensive experimental screening to establish empirically but emerges directly from TD-DFT calculations.

A systematic high-throughput TD-DFT analysis of 284 distinct graphene quantum dots varying in shape, size, and doping configurations with B, N, O, S, and P reveals clear design principles: the TDDFT literature usually consists of studies concerning few particular types of GQDs and these fragmented studies do not reveal the systematics of the effect of shape, size, and dopants on the emission spectrum of GQDs but high-throughput approaches overcome this fragmentation by systematically mapping the full design space [193]. Enyoh and Wang [24] applied in the first-principles work on defect-engineered CQDs, where an inverse relationship between exciton binding energy and optical brightness was established across eight defect configurations, identifying three optical classes: UV-bright emitters, visible absorbers, and NIR-active systems, each with distinct excitonic characters arising from specific defect configurations. Such computational design maps, condensing what would otherwise require hundreds of experimental syntheses and characterisation studies into a single computational campaign, are directly actionable for sensing design: for instance, identifying dopant configurations that position QD emission at wavelengths optimally separated from environmental matrix autofluorescence.

However, the prediction of exciton binding energies from first principles requires methods beyond standard TD-DFT, which systematically underestimates binding energies due to its local approximation to exchange-correlation effects. Many-body perturbation theory approaches, particularly the GW approximation for quasiparticle corrections combined with the Bethe–Salpeter equation (GW-BSE) for excitonic properties, provide quantitatively accurate binding energies at significantly higher computational cost.

### 9.3. Molecular Dynamics and QD–Analyte Binding Simulation

While DFT and TD-DFT characterise the electronic and optical properties of isolated QDs and specific QD-analyte configurations, the molecular dynamics (MD) simulation addresses the equally important questions of how analytes approach and bind to QD surfaces in realistic solution environments, and how binding geometry, which determines exciton-analyte distance and orbital overlap, depends on solvent conditions, surface ligand density, and analyte concentration.

Classical MD simulations can access nanosecond-to-microsecond timescales for QD-analyte systems in explicit solvent, capturing the full binding equilibrium and the distribution of binding geometries that governs ensemble-averaged sensing responses. For PFAS-QD interactions, the dynamics of per- and polyfluoroalkyl substances at interfaces in porous media are best approached through a computational roadmap spanning nanoscale MD simulation to macroscale modelling: molecular simulation methods, including DFT, hybrid quantum and classical MD, ab initio molecular dynamics (AIMD), and coarse-grained MD, each address different aspects of the PFAS-surface interaction, with classical MD providing nanosecond-timescale adsorption geometry prediction and AIMD providing picosecond-timescale charge transfer and electron excitation information [194]. For PFAS-QD sensor design specifically, MD simulations predict adsorption orientations of the perfluoroalkyl chain and charged head group at functionalised QD surfaces, information directly relevant to designing surface chemistries that maximise PFASs capture while minimising non-specific adsorption of competing matrix components. Molecular dynamics simulations of PFAS degradation under ultrasound treatment reveal that differences in degradation rates between PFCAs and PFSAs are primarily influenced by adsorption at the air-water interface determined by the molecular geometry of the headgroup, a result with direct implications for designing headgroup-selective QD surface ligands for PFAS discrimination [195].

The [196] computational study of GQD-heavy metal interactions established binding energies and heights for neutral and charged Cd, Hg, and Pb species on GQD surfaces, demonstrating that chemisorbed charged HM species act as typical acceptors while physisorbed neutral HM atoms are donor-like, a mechanistic distinction directly relevant to understanding why PET quenching efficiency differs between reduced and oxidised forms of the same heavy metal in QD-based sensing [196]. Ab initio MD (AIMD) provides electronic-level insight into how surface geometry reorganises upon analyte binding and how charge is redistributed across the QD-analyte interface, a microscopic picture of the CT exciton formation mechanism that is not accessible through any combination of experimental measurements.

### 9.4. Machine Learning for Property Prediction and Sensor Optimisation

The application of machine learning to QD-based sensing represents the most rapidly developing and potentially most transformative computational frontier in the field. ML addresses two distinct challenges: the prediction of QD properties from structural descriptors, enabling virtual screening of candidate materials; and the amplification of sensing signals through intelligent data processing, extracting analytical information from spectral or temporal data that conventional threshold-based analysis would miss.

For property prediction, ML models trained on DFT-calculated or experimentally measured QD property databases can predict bandgaps, quantum yields, and optical transition energies for new QD compositions and defect configurations orders of magnitude faster than DFT calculations. ML models, including Support Vector Regression (SVR), Nearest Neighbour Distance (NND), Random Forest (RF), Gradient Boosting Machine (GBM), Decision Tree (DT), and Deep Learning (DL), applied to CsPbCl_3_ PQD synthesis parameters, achieve high-accuracy prediction of QD size, absorbance, and photoluminescence properties with SVR and NND demonstrating the best accurate property prediction by achieving excellent performance on both test and training datasets, with high R^2^, low RMSE, and low MAE metric values [197]. The AI/ML-driven design and optimisation of quantum dots represents a perspective toward intelligent materials discovery that spans synthesis parameter optimisation, property prediction, and sensing performance enhancement, with ML becoming increasingly superior in its ability to understand the QD field and shape the future of nanomaterials design [198].

For sensing signal amplification, ML has demonstrated a capacity to extract analytical information from QD fluorescence data far beyond what conventional calibration curve analysis can achieve. Machine learning-enhanced evaluation of semiconductor quantum dots using data-driven approaches automates and expedites the evaluation process, proposing models that predict the applicability of a semiconductor quantum dot as a single-photon source with accuracy and efficiency inaccessible to manual characterisation, a paradigm directly translatable to environmental sensing where large QD libraries need rapid performance screening [199]. Deep learning-optimised N-CQD biosensors achieve detection limits of 5–10 nM for heavy metals—representing a 50–100-fold improvement over conventional CQD sensors through the combination of superior surface chemistry and CNN-LSTM attention-based spectral analysis with AI-based sensing methods, including ATTBeadNet models, optimised principal component analysis, and SVM-based systems achieving R^2^ = 0.97 ± 0.02 and RMSE below 4% across six contaminant classes simultaneously [161]. ML approaches coupled to low-dimensional, physically meaningful features such as ratiometric fluorescence ratios, electrochemical peak currents, or kinetic rate constants tend to generalise more reliably across samples and operating conditions than image-heavy or highly device-specific models [199], a finding with direct implications for designing ML sensing systems robust enough for real-world environmental deployment.

### 9.5. Inverse Design: From Target Sensing Properties to Optimal QD Architectures

The ultimate computational ambition in excitonic sensing design is inverse design: specifying the desired analytical performance, target analyte, required LOD, operating spectral window, and acceptable matrix tolerance and computationally identifying the QD architecture, defect configuration, and surface chemistry that achieves it. This reverses the conventional design workflow (synthesise → characterise → test → iterate) and replaces empirical iteration with targeted computation, dramatically compressing development timelines.

Inverse design for QD sensing is advancing rapidly from concept to practical application. A tandem deep neural network (DNN) structure, trained simultaneously on emission and absorption spectra as input datasets, successfully predicts the most probable layer thicknesses of InP/ZnSe/ZnS QDs that resemble on-demand target optical emission/absorption spectra, with transfer learning reducing the training data requirement for analogous CdSe/ZnSe/ZnS inverse design by up to 71.4% while maintaining an average prediction accuracy of 99.3% [200]. This result demonstrates the practical viability of DNN-based inverse design for core–shell QD architectures and establishes the transfer learning paradigm as a route to extending inverse design models across QD families without prohibitive data collection costs.

The Defect–Exciton Energy Map framework (Figure 5) provides the conceptual vocabulary for expressing inverse design targets in terms of excitonic parameters: binding energy, radiative rate, spectral position, and defect depth, which can be directly mapped to computational design objectives [24]. As training databases grow both from the published experimental literature and from systematic high-throughput computational campaigns, generative models, including variational autoencoders and generative adversarial networks trained on QD structure-property datasets, are beginning to propose novel QD architectures not represented in the existing experimental literature, expanding the accessible design space beyond what human intuition would explore [198]. The integration of DFT-computed Defect–Exciton Energy Maps with ML-driven sensor optimisation creates a uniquely powerful computational-experimental feedback loop: DFT identifies which defect configurations produce which excitonic parameters; ML predicts which excitonic parameters produce which sensing performances; and inverse design runs this combined pipeline in reverse to identify the synthesis targets most likely to achieve a specified analytical objective for a previously undetectable environmental contaminant.

## 10. Challenges, Gaps, and Future Directions

**Figure 10:**
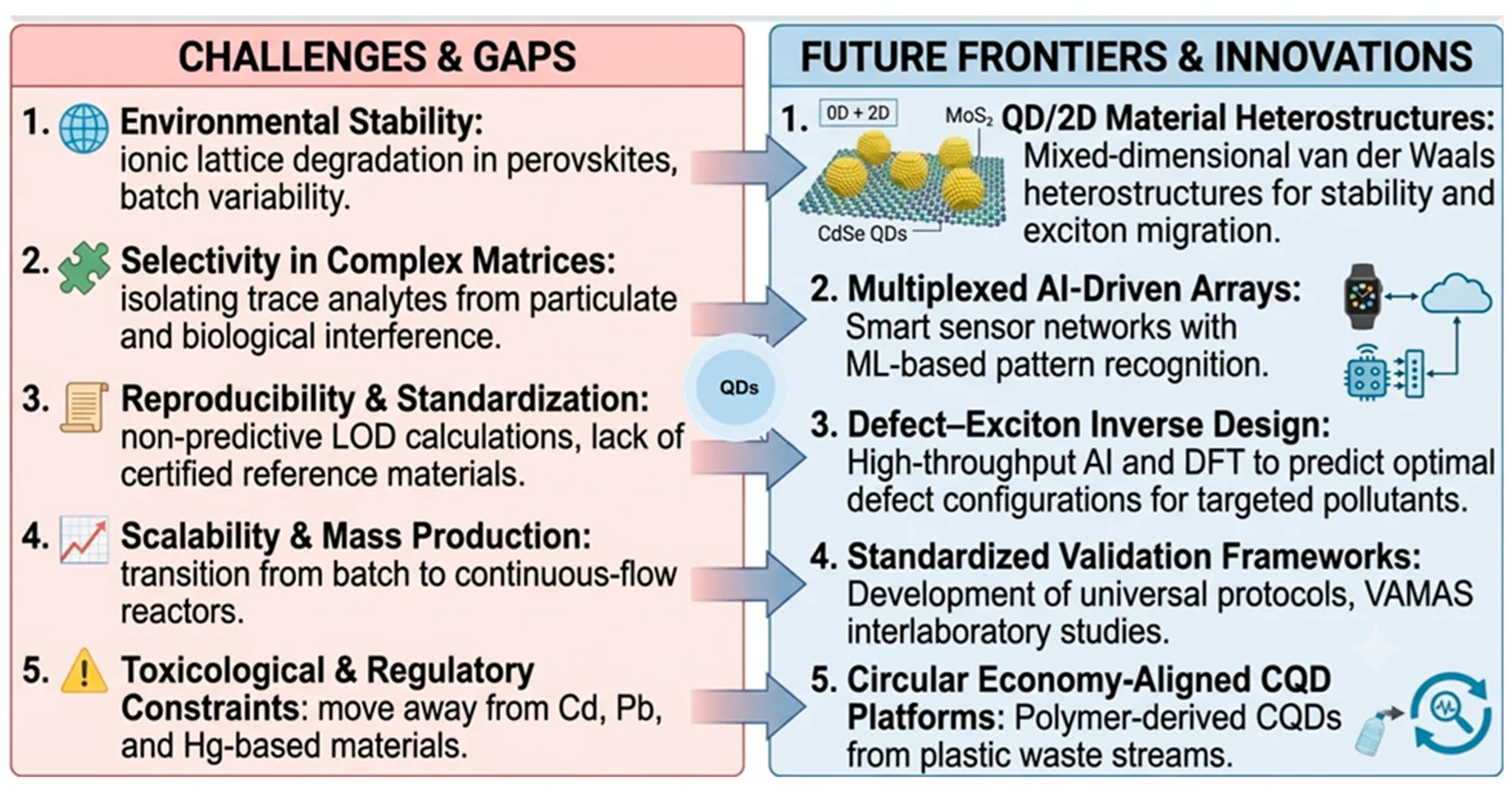
Strategic roadmap transitioning quantum dot (QD) excitonic sensors from laboratory research to environmental application. The left panel outlines the major technical and regulatory bottlenecks (1–5), including environmental degradation, matrix interference, and mass production constraints. The right panel displays the corresponding future frontiers and technological innovations (1–5) designed to address these gaps, highlighted by mixed-dimensional 0D/2D van der Waals heterostructures, artificial intelligence-driven predictive inverse design, and sustainable CQD loops aligned with a circular economy.

Excitonic transduction in QD-based sensors has progressed from empirical fluorescence-based observations toward mechanistic understanding and increasingly predictive design approaches across diverse QD families, pollutant classes, and sensing modalities. However, the transition from laboratory demonstrations to deployable, field-validated, and regulatory-ready environmental sensing technologies remains limited by challenges beyond material discovery. These include long-term environmental stability, selectivity in complex matrices, reproducibility, scalability, toxicological considerations, and integration into intelligent monitoring systems (Figure 10). Addressing these barriers will determine whether excitonic QD sensing evolves from an active research field into a practical environmental technology.

### 10.1. Environmental Stability

Environmental stability remains the most significant challenge preventing QD sensors from progressing beyond controlled laboratory conditions. Real environmental monitoring requires consistent excitonic behaviour under fluctuating temperature, humidity, ionic strength, pH, UV exposure, and biological interference. However, the stability limitations differ significantly among QD families and require material-specific solutions.

Perovskite QDs possess highly attractive excitonic properties but remain limited by their ionic lattice structure, which promotes degradation under moisture, heat, and illumination. These processes cause phase transformation, reduced PLQY, and irreversible structural changes, requiring strategies, including ligand engineering, surface passivation, and encapsulation within polymeric or porous matrices [21]. A key challenge in perovskite-based gas sensing is balancing ligand protection with analyte accessibility: long-chain ligands improve stability but restrict analyte interaction, whereas shorter or zwitterionic ligands enhance accessibility at the expense of environmental resistance [21]. CQDs generally exhibit superior aqueous stability but face challenges associated with prolonged UV exposure, extreme pH, and high ionic-strength environments where aggregation and surface-state reorganisation may alter excitonic behaviour. Additional limitations include quantum yield losses exceeding 35%, ambient stability concerns, and batch variability (~±15%), which directly affects calibration reliability between synthesis batches [75]. Future CQD deployment requires standardised synthesis protocols, improved purification, and quality-control parameters extending beyond quantum yield to include exciton lifetime distributions and surface-state characterisation. For electrochemical QD sensors operating in complex environmental matrices, achieving simultaneous sensitivity, selectivity, and long-term stability remains challenging due to interference from organic matter, ions, and biological components [201]. Future stability assessment should therefore move beyond ideal laboratory testing toward realistic ageing studies under environmental stress conditions, supported by standardised protocols comparable to those used for photovoltaic materials.

### 10.2. Selectivity in Complex Matrices

A persistent limitation in the QD sensing literature is the gap between excellent selectivity demonstrated in simplified laboratory solutions and performance under realistic environmental conditions. Many studies rely on selected interferents and artificially spiked samples at concentrations significantly higher than those encountered in natural systems. However, practical monitoring requires reliable detection at environmentally relevant levels, including nanomolar-to-picomolar concentrations for PFASs, trace heavy metals in background-contaminated waters, and low concentrations of microplastics within complex particulate matrices.

Selectivity is strongly dependent on the excitonic transduction mechanism. Sensors based on trap-state generation or inner filter effects (IFEs), such as those commonly used for metal ions and Cr^6+^ detection, may suffer from non-specific responses because multiple species can perturb surface states or optical absorption. In contrast, recognition-driven strategies such as aptamer-mediated FRET and MIP-based exclusion provide greater molecular specificity. Despite progress, improvements in recovery accuracy, stability, and performance in authentic samples remain key priorities for QD luminescent sensors [100]. Emerging solutions include QD sensor arrays combined with chemometric or machine-learning analysis, molecularly imprinted QD surfaces for selective enrichment, and CQD@MOF hybrids that integrate porous molecular recognition with excitonic signal generation. Recent advances in CQD@MOF design and surface functionalisation demonstrate their potential for improving both selectivity and sensitivity in environmental and food safety applications [201].

### 10.3. Reproducibility, Reporting, and Validation

Beyond materials optimisation, the most fundamental barrier limiting the translation of QD sensors into environmental practice is the absence of standardised approaches for fabrication, calibration, validation, and performance reporting. The current literature frequently reports exceptional sensing performance under optimised laboratory conditions, yet comparisons between studies remain difficult due to inconsistent definitions of selectivity, variable LOD calculations, different validation criteria, and limited evaluation under realistic environmental conditions. A comprehensive analysis of 124 peer-reviewed QD sensor studies revealed geometric mean detection limits of 38 nM for fluorescence-based sensors and 0.109 pM for chemiluminescence-based sensors. However, the same analysis showed no significant improvement in protein detection LOD values between 2015 and 2025, suggesting that continued advances in synthesis have not consistently translated into improved analytical performance in complex environments [100]. This highlights that the current limitation is not necessarily a lack of new QD materials, but rather insufficient standardisation and validation frameworks.

Future progress requires the adoption of consistent analytical protocols, including IUPAC-aligned LOD and LOQ calculations, mandatory testing in authentic environmental matrices, certified reference materials for priority pollutants, and interlaboratory comparison studies through organisations such as VAMAS or ASTM. Although less visible than new material discoveries, these developments are essential for establishing confidence in QD sensors and enabling their transition from academic demonstrations to regulatory and environmental monitoring applications.

### 10.4. Scalability, Cost, and Green Synthesis

Large-scale deployment of QD sensors requires addressing manufacturing reproducibility, economic feasibility, and environmental sustainability. A major challenge is batch variability, where eco-friendly QDs such as InP, graphene QDs, and CuInS_2_ may exhibit approximately ±15% variation, which is acceptable for laboratory studies but incompatible with commercial sensor production requiring consistent analytical performance [75]. Transitioning from conventional batch synthesis toward continuous-flow approaches, including microfluidic and tubular reactors, will be necessary to achieve controlled nucleation, growth, and reproducible excitonic properties.

CQDs represent one of the most promising routes toward sustainable QD sensor manufacturing because they can be produced from renewable resources and waste carbon feedstocks while avoiding toxic organometallic precursors and harsh solvents associated with many inorganic QDs. The development of environmentally compatible QDs, including InP, graphene QDs, and CuInS_2_ through hot injection, solvothermal, and thermal decomposition strategies, reflects the broader movement toward replacing cadmium-, lead-, and mercury-containing materials restricted by regulatory concerns [75].

Combining waste-derived CQDs with scalable fabrication approaches, including inkjet, screen, and aerosol jet printing onto flexible substrates, offers a pathway toward low-cost, environmentally responsible, and decentralised sensor technologies.

### 10.5. Toxicological and Regulatory Considerations

The environmental application of QD sensors presents a dual responsibility: the sensor must detect pollutants without introducing additional contamination, and its long-term environmental impact must be assessed. This issue is particularly relevant for Cd-based QDs, where potential heavy-metal release may create ecological and health risks. These concerns are amplified by the increasing global burden of electronic waste, which reached 62 million metric tonnes according to the UN Global E-waste Monitor 2024, with heavy metal emissions representing a significant component of industrial pollution [75]. Regulatory trends increasingly favour safer alternatives, including cadmium-free (InP, CuInS_2_, ZnSe), lead-free (bismuth halide and tin perovskite), and carbon-based QDs. Importantly, these restrictions are also driving innovation by encouraging the development of materials with improved safety profiles while maintaining competitive excitonic performance. CQDs and InP QDs, in particular, demonstrate that environmentally compatible materials can provide strong optical properties and sensing capabilities without the limitations associated with toxic predecessors [75]. Recent advances in lead-free perovskite systems, such as Cs_3_Bi_2_X_9_ and CsSnX_3_, provide improved aqueous stability, while PQD@MOF composites and ratiometric architectures enhance selectivity and reliability in complex matrices. These examples demonstrate that toxicological constraints can serve as a catalyst for the development of more advanced sensing platforms rather than simply restricting existing approaches [96].

### 10.6. Future Directions: The Next Frontier

Future progress in excitonic QD environmental sensing will likely emerge from the integration of advanced materials, computational design, artificial intelligence, and sustainable manufacturing strategies.

*QD/2D Material Excitonic Heterostructures.* Combining QDs with two-dimensional materials, including graphene, transition metal dichalcogenides (MoS_2_, WS_2_, WSe_2_), and hexagonal boron nitride, creates mixed-dimensional van der Waals heterostructures with interfacial excitonic interactions unavailable in individual materials. In these systems, QDs provide tunable optical properties, while 2D materials facilitate charge transport and exciton migration, enabling enhanced sensing performance [54,202]. The high surface area, tunable electronic structures, and versatile surface chemistry of graphene, TMDs, MXenes, and related heterostructures further expand opportunities for highly sensitive environmental sensing when integrated with QD excitonic transduction mechanisms [203].

*IoT-Integrated and Wearable Excitonic Sensors.* Integration with IoT platforms provides a realistic pathway toward distributed and real-time environmental monitoring. Recent demonstrations include GQD/polyimide humidity sensors showing a 96.36% sensitivity improvement and GQD-ZnO nanocomposites capable of detecting CO within 1–100 ppm with 90% accuracy. Combined with low-power communication technologies such as LoRaWAN, these systems enable remote monitoring networks with automated environmental alerts [204]. The compatibility of QDs with solution-based fabrication methods and flexible substrates, together with their tunable optical response across UV, visible, and infrared regions, makes them particularly attractive for wearable and portable sensing platforms [205].

*Multiplexed AI-Driven Excitonic Sensor Arrays.* Future environmental monitoring will likely move beyond single-analyte detection toward intelligent sensor networks. Arrays containing QDs with different compositions and surface chemistries can generate unique excitonic response patterns, which can be analysed using machine-learning algorithms to identify multiple pollutants simultaneously without separation steps. The integration of AI with 2D nanomaterial-based sensors and IoT systems offers a pathway toward autonomous environmental monitoring platforms capable of extracting complex chemical information from multidimensional sensor responses [203].

*Defect–Exciton Inverse Design for Targeted Pollutants.* The combination of high-throughput calculations, TD-DFT databases, machine-learning predictors, and experimental validation is enabling a shift from empirical sensor development toward predictive design. The Defect–Exciton Energy Map provides a conceptual framework for identifying defect configurations that maximise excitonic responses toward specific pollutants by considering interactions such as coordination chemistry, electron affinity, and dielectric effects [24]. This inverse-design strategy has the potential to significantly accelerate the development of QD sensors for emerging contaminants by predicting optimal structures before experimental synthesis.

*Circular Economy-Aligned CQD Platforms.* Waste-derived CQDs represent a unique convergence of sustainability and environmental sensing. The synthesis of CQDs from plastic waste streams demonstrates that these materials are not simply greener alternatives but possess distinct excitonic properties linked to their precursor chemistry. Polymer-derived CQDs, such as PA-based CQDs, exhibit selective responses toward Polyethylene terepthalate microplastics, suggesting that waste-derived CQDs can function as polymer-specific sensing platforms [62,102]. Developing libraries of CQDs derived from diverse environmental waste streams could establish a circular sensing framework in which waste materials are transformed into analytical tools for monitoring their own environmental impact.

## 11. Conclusions

This review has established a mechanistic framework for understanding excitonic transduction in quantum dot (QD)-based environmental sensing. Rather than being an empirical consequence of material selection, sensing performance defined by sensitivity, selectivity, dynamic range, and matrix tolerance is governed by excitonic parameters that can be understood, modelled, and rationally engineered. Across QD families, defect states, sensing mechanisms, computational approaches, and pollutant applications, the exciton emerges as the central link between molecular recognition events and measurable optical responses.

The fundamental principle is that the exciton is the universal signal carrier, and its modulation represents the core design challenge. Changes in photoluminescence intensity, spectral position, lifetime, energy transfer, or electrochemiluminescent output all originate from perturbations of excitonic properties, including binding energy, radiative dynamics, spatial localisation, and fine structure. The eight transduction pathways discussed in this review, including quenching, trap modulation, surface passivation, AIE, FRET, ratiometric sensing, charge-transfer excitons, lifetime-based detection, and ECL, should therefore be viewed as a mechanistic design map rather than isolated sensing strategies. The optimal sensing response depends on the interplay between analyte chemistry, QD electronic structure, and surface functionality. No single QD family provides a universal solution. Instead, carbon, chalcogenide, perovskite, and III–V QDs offer complementary excitonic advantages. CQDs provide sustainable synthesis routes, accessible surface states, biocompatibility, and defect-mediated tunability; chalcogenide QDs remain the most established platforms for controlled exciton engineering, FRET, and ratiometric sensing; perovskite QDs provide exceptional optical properties and strong sensitivity to environmental perturbations; while InP-based QDs offer a pathway toward less toxic, near-infrared-responsive sensing platforms. Future progress requires moving beyond material selection by convention toward mechanism-driven QD design. Defect engineering represents one of the most powerful yet underexploited strategies for controlling excitonic sensing behaviour. The Defect–Exciton Energy Map introduced in this review provides a framework for relating defect depth, density, location, and chemical character to specific excitonic responses and sensing outcomes. Combined with first-principles calculations, TD-DFT, and machine-learning-guided prediction, this approach enables a transition from empirical optimisation toward inverse design, where target sensing properties guide the selection of suitable QD structures and defect configurations.

Despite significant progress, real-world validation remains a major challenge. Many reported sensing systems rely on simplified laboratory conditions, including ultrapure water matrices, artificial spikes, and concentrations above environmentally relevant levels. Advancing QD sensors toward practical deployment requires standardised performance evaluation, authentic-matrix testing, and reporting practices aligned with environmental monitoring requirements. Addressing these challenges will be essential for translating excellent laboratory performance into reliable field technologies. The integration of computational design, experimental synthesis, and machine-learning-assisted analysis represents the next frontier of excitonic sensing. High-throughput simulations, structure–property databases, and intelligent signal processing are increasingly enabling predictive control over QD behaviour, demonstrating that sensing performance depends not only on material design but also on data-driven interpretation of excitonic responses. Ultimately, excitonic QD sensors provide a powerful platform for addressing emerging environmental challenges, including heavy metals, PFASs, microplastics, pesticides, pharmaceuticals, and reactive species. Their ability to translate nanoscale chemical interactions into tunable optical signals positions them as promising tools for real-time, sensitive, and decentralised environmental monitoring. The excitonic transduction framework developed in this review provides a roadmap for designing the next generation of QD sensors linking material structure, computational prediction, and sensing performance toward improved protection of environmental and human health.

## Figures and Tables

**Table 1:** Comparative excitonic properties of major QD families for environmental sensing.

Property	CQDs	CdSe/ZnS	CsPbBr_3_ PQDs	InP/ZnS	Key References
Exciton Bohr radius	~1–3 nm (sp^2^ domain)	~5.6 nm	~3–7 nm	~10–11.3 nm	[12,30,66]
Exciton binding energy	Variable; surface-state dependent	10–25 meV (enhanced by confinement)	40–150 meV	~20–60 meV	[21,67]
PLQY	10–80% (dopant-dependent)	50–95% (core–shell)	70–95%	60–90% (multishell)	[20,72,74]
Emission FWHM	60–100 nm (excitation-dependent)	25–40 nm	12–25 nm	35–50 nm	[20,53]
Spectral tunability	UV–NIR (via doping/surface chemistry)	UV–NIR (size-dependent)	420–700 nm (halide composition)	480–845 nm (size/shell-dependent)	[22,75]
Aqueous stability	High	Moderate (ligand-dependent)	Low (ionic lattice sensitive)	Moderate (shell-dependent)	[69,75,76,77]
Toxicity profile	Low (carbon-based)	High (Cd^2+^, RoHS-restricted)	Moderate (Pb^2+^)	Low (Cd/Pb-free)	[66,75]
Characteristic excitonic feature relevant to sensing	Surface-state accessibility; AIE-capable	Wide established surface chemistry library	Highest E_b_; narrowest emission linewidth	NIR-accessible; reduced blinking	[24,47]

**Table 2:** Study-level performance data for QD-based sensors across major environmental pollutant classes.

Pollutant	QD Material	Recognition Chemistry	Assay Format	Matrix	Linear Range	LOD (Method)	Recovery (%)	Real-Sample Validated	Primary Reference
Hg^2+^	ZnO/C dual-QD	Surface coordination (Hg^2+^–QD interaction)	Fluorescence quenching (turn-off)	Wastewater	1–25 μmol/L	0.347 μmol/L	99.6–100.4% (RSD < 5%)	Wastewater	[150]
Hg^2+^	N-GQDs	Surface thiol coordination	Fluorescence on-off-on	Environmental water	0–4.31 μM	23 nM	95–105.8%	River water	[151]
Hg^2+^	Si-QDs	Electron transfer at Si-QD surface	Fluorescence quenching (smartphone)	Environmental water	0.5–5.0 μmol/L	28 nmol/L	94.8–97.1%	Water	[152]
Hg^2+^/Fe^3+^	GQDs (NN-functionalised)	NN moiety coordination + ML	Fluorescence + ML discrimination	Natural/drinking water	NR	0.001 mg/L Hg^2+^; 0.003 mg/L Fe^3+^	Validated vs. CV-AFS	Natural and drinking water.	[153]
Pb^2+^/chlortetracycline	N/P-CQDs	Surface coordination	Dual fluorescence quenching	River water, tomato, milk	5–100 μM/0–100 μM	18 μM/30 nM	92–98%	River water, food	[154].
Cd^2+^	CQDs/CdTe QD ratiometric	Surface coordination + ratiometric	Ratiometric fluorescence (smartphone)	Rice samples	0.1–23 μM	0.018 μM	NR	Rice	[155]
Cr^6+^	N-CQDs	IFE (Cr^6+^ UV absorption)	Fluorescence quenching	Water samples	2–120 μM	2.07 μM	NR	Water	[97]
PFOA	Si-QD/MIP	Molecular imprinting (MIP)	Dual-mode fluorescence + colorimetric (smartphone)	Environmental water, food	NR	37.5 nmol/L (fluorescence); 73.9 nmol/L (colorimetric)	NR	Water and food	[156]
PFASs (PFCAs)	Dual fluorescent NPs	MIP + ratiometric	Ratiometric fluorescence (microfluidic)	Environmental water	NR	Sub-100 nM	NR	Water	[157]
Chlorpyrifos	CQD@MIP	Molecular imprinting (MIP) + PET	Fluorescence quenching	Water	NR	ng/mL range	NR	Water	[158]
Malathion	Sulphur-QD (AIE-ECL)	Aptamer + AIE-ECL	Electrochemiluminescence aptasensor	Not reported	1 × 10^−13^–1 × 10^−8^ mol/L	0.219 fM	NR	NR	[108]
Microplastics (PET)	PA-CQDs (~1.4 nm)	Surface-state modulation	EEM-PARAFAC fluorescence	Tap water	0.0005–10 mg/L	1.67 mg/L (PET, 10 μm)	Fingerprints preserved in tap water	Tap water	[34]
Tetracycline hydrochloride	Zn-doped CQDs	ICT + IFE + ratiometric	Ratiometric fluorescence	Pharmaceutical, water	0.1–50 μM	61.1 nM	NR	River water and milk	[159]
ONOO^−^ (ROS)	CDs (CD–N-I FRET probe)	Naphthalimide–isatin receptor + FRET	Ratiometric FRET fluorescence	Live cells/biological	0–25 μM	0.22 μM	NR	HepG2 cells, APAP model	[104]

LOD values are as reported in the cited primary study. LOD calculation method is given where stated in the source paper (3σ/slope is the most common). Recovery values are from spiked real-sample experiments. NR indicates data not reported in the cited study. Cross-row comparisons should be interpreted with caution given heterogeneous analyte concentrations, matrices, assay formats, recognition chemistries, and LOD calculation methods across studies.

**Table 3:** Indicative comparative performance of QD-based excitonic sensors and competing sensing platforms for environmental pollutant detection.

Platform	Indicative LOD Range	Typical Response Time	Primary Selectivity Basis	Photostability	Multiplexing Potential	Matrix Robustness	Toxicity/Regulatory Status	Field Deployability (Reported or Inferred)	Representative References
Excitonic QD sensors (CQDs)	fM–μM	Seconds–minutes	Surface chemistry; energy-level alignment; recognition element	High (weeks–months)	High (spectral encoding)	Moderate; IFE and scattering correction required	Low toxicity; no RoHS restriction	Moderate–high (smartphone formats demonstrated)	[34,39,100,108]
Excitonic QD sensors (CdSe/CdTe)	pM–μM	Seconds–minutes	Surface chemistry; recognition element	High	High	Moderate	High toxicity (Cd^2+^; RoHS-restricted)	Moderate (laboratory-centric; portability limited by toxicity concerns)	[100,172]
Excitonic QD sensors (PQDs)	pM–nM	Seconds	Surface chemistry; cation exchange; FRET	High (dry); Low (aqueous)	High	Low (aqueous instability limits real-sample use)	Moderate (Pb^2+^; regulatory scrutiny)	Low (aqueous instability); Moderate (encapsulated formats)	[96]
Organic fluorescent dyes	nM–μM	Seconds	Molecular structure	Low (photobleaching within hours–days)	Low (spectral overlap limits multiplexing)	Low–moderate	Low	Moderate	[173]
Upconversion nanoparticles (UCNPs)	nM–μM	Seconds–minutes	LRET with surface-bound receptor	Very high	Moderate	High (NIR excitation avoids autofluorescence)	Low	Low (high-power NIR laser required)	[174]
Plasmonic NPs (LSPR)	nM–μM	Minutes	Refractive index change	Very high	Low	Moderate	Low	Moderate	[175]
Plasmonic NPs (SERS)	fM–pM	Minutes–hours	Vibrational fingerprint	Very high	High	Low (hot-spot reproducibility limits quantification)	Low	Low (reproducibility challenge)	[175]
Aptasensors	pM–nM	Minutes–hours	3D molecular recognition (SELEX-derived)	High (DNA stable)	Moderate	Moderate	Low–moderate	Moderate	[145,176]
MIPs	nM–μM	Minutes	Shape- and functional group-complementary binding	Very high	Low	High	Low	High (demonstrated in field formats)	[156,177]
Enzyme-based sensors	nM–μM	Minutes	Catalytic specificity (substrate/inhibitor)	Low (enzyme instability in field conditions)	Very low	Low (enzyme inhibition by matrix components)	Moderate	Low	[178]

**Table 4:** Summary of computational approaches to excitonic sensing design.

Computational Method	Key Applications & Findings	Benefits & Implications for Sensing Design	Key References
Density Functional Theory (DFT)	- Ground-state properties (geometries, DOS, HOMO-LUMO gaps, charge distributions, defect states). - Hybrid functionals (HSE06, B3LYP, etc.) improve bandgap accuracy over GGA (e.g., PBE). - Defect/doping effects in CQDs/GQDs; halide alloying in PQDs; functionalised GQD-heavy metal interactions (thiol vs. amino groups). - Photodegradation mechanisms (e.g., sulphur vacancy pairs in core–shell QDs).	Rational design of defects, dopants, and surface groups; predicts selectivity (e.g., Pb/Cd affinity); guides stability via interfacial engineering; shifts sensor development from empirical screening to computational prediction.	[67,187,188,189,190]
Time-Dependent DFT (TD-DFT)	- Optical properties: absorption/emission spectra, oscillator strengths, excitonic character. - Impact of dopants, edge modifications, and functional groups (e.g., carbonyl for charge-transfer states in CQDs). - High-throughput screening of hundreds of GQDs (shape, size, B/N/O/S/P doping). - Inverse relationships (e.g., exciton binding energy vs. brightness); optical classification of defect configurations.	Identifies optimal configurations for specific wavelengths, charge-transfer sensing (e.g., PET), and reduced autofluorescence interference; high-throughput design maps replace extensive experiments; GW-BSE for more accurate binding energies.	[24,187,191,192,193]
Molecular Dynamics (MD) & AIMD	- Analyte binding dynamics, geometries, and equilibria in explicit solvent. - PFAS adsorption orientations and surface chemistry optimisation. - Heavy metal binding (charged vs. neutral species as acceptors/donors). - Charge redistribution and interface reorganisation upon binding.	Predicts real-world binding in solution; informs ligand design for selectivity and reduced non-specific binding; mechanistic insight into PET/CT processes; supports degradation studies and headgroup-selective sensing.	[194,195,196]
Machine Learning (ML)	- Property prediction (bandgaps, quantum yields, size, PL) from structural/synthesis descriptors using SVR, RF, GBM, DL, etc. - Signal amplification: CNN-LSTM, ATTBeadNet, SVM for spectral/temporal data analysis. - Heavy metal detection limits improved 50–100×; multi-contaminant classification.	Virtual screening and rapid library evaluation; automates optimisation; extracts subtle signals beyond traditional calibration; enables robust, generalisable real-world environmental sensors.	[161,197,198,199]

## Data Availability

No new data were created or analysed in this study. Data sharing is not applicable to this article.
